# Antifungal Activity of Select Essential Oils against *Candida auris* and Their Interactions with Antifungal Drugs

**DOI:** 10.3390/pathogens11080821

**Published:** 2022-07-22

**Authors:** Ryan A. Parker, Kyle T. Gabriel, Kayla D. Graham, Bethany K. Butts, Christopher T. Cornelison

**Affiliations:** BioInnovation Laboratory, Department of Molecular and Cellular Biology, Kennesaw State University, Kennesaw, GA 30144, USA; rparke67@kennesaw.edu (R.A.P.); kgabrie5@kennesaw.edu (K.T.G.); kgraham598@outlook.com (K.D.G.); bethanykbutts@gmail.com (B.K.B.)

**Keywords:** *Candida*, essential oils, antifungal, drug resistance

## Abstract

*Candida auris* is an emerging fungal pathogen that commonly causes nosocomial blood infections in the immunocompromised. Several factors make this pathogen a global threat, including its misidentification as closely related species, its ability to survive for weeks on fomites, and its resistance to commonly prescribed antifungal drugs, sometimes to all three classes of systemic antifungal drugs. These factors demonstrate a need for the development of novel therapeutic approaches to combat this pathogen. In the present study, the antifungal activities of 21 essential oils were tested against *C. auris*. Several essential oils were observed to inhibit the growth and kill *C. auris*, *Candida lusitaniae*, and *Saccharomyces cerevisiae* when in direct contact and at concentrations considered safe for topical use. The most effective essential oils were those extracted from lemongrass, clove bud, and cinnamon bark. These essential oils also elicited antifungal activity in gaseous form. The efficacies of formulations comprised of these three essential oils in combination with fluconazole, amphotericin B, flucytosine, and micafungin were explored. While synergism was neither observed with cinnamon bark oil nor any of the antifungal drugs, lemongrass oil displayed synergistic, additive, and indifferent interactions with select drugs. Formulations of clove bud oil with amphotericin B resulted in antagonistic interactions but displayed synergistic interactions with fluconazole and flucytosine. These essential oils and their combinations with antifungal drugs may provide useful options for surface disinfection, skin sanitization, and possibly even the treatment of *Candida* infections.

## 1. Introduction

Drug-resistant pathogens are becoming an increasing threat to human health [1]. Pathogens once easily treated are becoming increasingly more difficult to treat due to multi-drug resistance, leading to an increased incidence of debilitating and fatal infections. Adding to this problem, the rise in conditions that compromise the immune system is facilitating infection by opportunistic pathogens [2]. These conditions open the possibility of infection by organisms previously thought to be harmless, including many fungi. Low and Rotstein observed a threefold increase in deaths from invasive fungal infections between 1981 and 1996 [2], and current estimates place the global incidence of several invasive fungal infections at 1,000,000 cases or more each year (Table 1) [3].

Among these fungal opportunists are species such as *Candida albicans* and *Aspergillus fumigatus* [2]. Many of these organisms are ubiquitous in the environment and human commensals [4,5]. Individuals are often at risk of exposure to these organisms from the environment or when they come into contact with asymptomatic carriers, in addition to those showing clear signs of infection. While many of these infections are non-life-threating, some can escalate to systemic infections in the immunocompromised [6]. Many times, these infections occur in healthcare settings where both vulnerable people and potential pathogens are concentrated in otherwise unrealized high densities. Even with therapeutic intervention, these infections can be deadly. Multiple studies report mortality rates as high as 50% in patients treated for invasive fungal infections [7,8].

To date, only three classes of antifungals are widely used and considered safe to treat invasive fungal infections. Echinocandins, such as micafungin, inhibit fungal cell membrane formation by disrupting the synthesis of the structural component 1,3-β-d-glucan [9]. Azoles, such as fluconazole, inhibit the bioosynthesis of the cell membrane component ergosterol, while polyenes, such as amphotericin B, bind to membrane ergosterol to induce pores and cause cell death through the leakage of cytosolic components [9]. In addition to these, a nucleoside analog, flucytosine (5-fluorocytosine or 5-FC), is sometimes used in combination with other drugs as a treatment, and acts by inhibiting the synthesis of pyrimidine and its incorporation into larger nucleic acids [9,10]. Despite the presence of multiple treatment options, pathogen resistance to all three classes of these drugs has been on the rise [11,12]. In addition, multiple modes of resistance have been discovered for each class, thereby complicating the task of overcoming drug resistance [13]. This presents a need for effective therapeutic options for these deadly infections.

The development of antifungal drugs is often more complicated than developing antibiotics to treat bacterial infections, with much of this problem being rooted in the genetic and cellular homology between a fungal pathogen and its eukaryotic host, resulting in an increased risk of unintended systemic toxicity to the host [14]. As such, the drugs used to treat fungal infections are often harmful to the patient, especially at the elevated doses needed to treat tolerant and resistant strains [15]. This limits the availability of drug targets when developing new drugs, exacerbating the problem. Even though many antifungals are deemed safe to use, they often have significant harmful side-effects [15]. This not only leads to the slow development of new drug-based therapeutic options, but also places a large burden of cost on patient support systems. Not only are new and effective drugs required, but expedient, safe, and affordable solutions to address ongoing outbreaks are of urgent need.

One such organism that exemplifies the issue of emerging drug-resistant fungal pathogens is *Candida auris*. *C. auris* was originally isolated from the inner ear of a patient at a Japanese hospital in 2009 [16]. Since its initial isolation, it has garnered media attention as a new “super bug”, with several unique strains from four geographically distinct clades being identified from clinical cases worldwide (Table 2) [17,18]. These clades are purported to have emerged simultaneously, and their identity has been confirmed by whole-genome sequencing, which has identified thousands of single-nucleotide polymorphisms [17].

*C. auris* was originally described as being able to grow well in temperatures as high as 40 °C, while most other closely related species survived only at lower temperatures [16]. A recent report suggests that this tolerance may be related to increasing global temperatures [19]. Another report claims that *C. auris* could remain viable for as long as 14 days on soiled healthcare surfaces [20]. The same study reported enzymatic activity for *C. auris* persisted for as long as 28 days post-inoculation [20]. Persistence and reinfection by this organism are enhanced by its transmissibility between surfaces and hosts. Examinations of several early hospital outbreaks detected *C. auris* on a variety of surfaces in contact with patients, including medical instruments, bedsheets, and sinks, among others [21]. A critical step to preventing its spread is to follow strict disinfection protocols; however, these are still in development. Complicating the development of these protocols is its high tolerance to commonly used disinfectants, including ethanol [22]. The Environmental Protection Agency is continually updating its guidelines for testing potential disinfectants with *C. auris*, and the Centers for Disease Control and Prevention (CDC) have recommended the use of chemicals approved for use with the endospore-producing bacterium *Clostridium difficile* for eliminating *C. auris* from surfaces [23].

The most alarming trait of *C. auris* is its propensity for drug resistance. Most known strains are resistant to at least one antifungal drug, most commonly fluconazole [24]. Many strains exhibit multi-drug resistance, and some have been found to be resistant to all three major classes of antifungal drugs [24]. This trait is not specific to strains from any one geographic region, either. Isolates from each clade have been identified to be resistant to at least one class of antifungal [25].

Plants and their extracts have long been utilized to treat a variety of ailments, including infectious disease. As plant cells are fixed in place and do not have a circulating immune system, other defense mechanisms have been developed to combat pathogens [26]. One of these mechanisms is the production of antimicrobial compounds, many of which are of low molecular weight and volatile, often resulting in a pronounced odor [26]. The volatile nature and odor have drawn human interest in extracting these compounds for use as perfumes and other scented products. Essential oils are produced by separating, or extracting, these volatile compounds from plant matter by steam distillation or cold pressing, among other methods. Dhifi et al. provided a comprehensive review of the chemistry and biological activities of essential oils [27].

Many essential oils have antimicrobial properties that are known to inhibit viruses, bacteria, and fungi [28,29,30,31]. This makes essential oils an attractive reservoir for antimicrobial treatments. They are readily available and generally inexpensive, increasing their accessibility as treatment options. One issue with their use as therapeutic agents has been cytotoxicity, with elevated doses able to damage human cells [32]. Thus, many extracts must be diluted to avoid negative symptoms and cellular damage when used topically [32]. Another issue is variability among plant growth conditions and extraction methods that can alter the ratios of chemical compounds present in the extracted essential oils, making the identification of active ingredients and standardization challenging [27]. Interestingly, the volatile nature of essential oils has led to them being used in aromatherapy, with some reports claiming they retain their antimicrobial properties in a gaseous state [31,33].

Combination therapy, or the use of multiple medications in combination, has been used to treat difficult drug-resistant infections [34]. A classic example is co-amoxiclav (augmentin), which is a combination of amoxicillin and clavulanic acid used to treat penicillin-resistant bacteria [35]. Another example of combination therapy used against a fungal pathogen is the combination of amphotericin B and flucytosine to combat the fungal pathogen *Cryptococcus neoformans*, with one study observing enhanced killing of all isolates tested [36]. Mukherjee et al. have written a comprehensive review detailing guidelines for the validation and use of treatment combinations [34]. Recent evidence also suggests that essential oils can function in a synergistic association with antimicrobial drugs, including antifungals [30,37,38]. This is a desirable outcome because, as previously mentioned, antifungal drugs are expensive and can cause side effects that can be severe at the elevated doses needed to treat infections by resistant organisms. This phenomenon can restore the utility of antimicrobials that have mostly been abandoned due to widespread resistance. Given that antimicrobials are being rendered increasingly ineffective due to drug-resistant organisms, the benefits of exploring essential oils are at least twofold. They could elicit efficacious antimicrobial activity when used as topical antiseptics for skin decolonization and as disinfectants for contaminated surfaces. They could also provide treatment options for fungal infections, when used either alone or in combination with the antifungal drugs that exhibit synergism. Even in the absence of synergism, combination therapy can produce additive results. In this case, the components contribute to the overall effectiveness of the treatment, without necessarily enhancing the effects of the other component. Thus, the overall effective dose remains the same, but reduces the required amount of each component. In some cases, this could reduce the risk of undesirable side effects of the components when used in combination.

This study examined the antimicrobial effects of 21 essential oils, both in direct contact and in gaseous form. The interactions of the three most effective oils and four commonly used antifungal drugs were explored.

## 2. Results

### 2.1. Antifungal Activity

All examined essential oils except those from eucalyptus, lemon, grapefruit and bitter orange elicited inhibitory effects at the concentrations tested (Table 3 and Table 4). Essential oils of bergamot, cinnamon bark, cinnamon leaf, clove bud, geranium, lemongrass, lime peel, peppermint, spearmint, and tea tree all elicited inhibitory activity at concentrations considered safe for dermal use (Table S2). Essential oils of basil and lavender yielded minimum inhibitory concentration (MIC) values above the recommended safe concentrations, and the remainder had no maximum concentration listed (Table S2). Of the essential oils that were observed to elicit lethal activity against *C. auris*, only essential oils of bergamot, lavender, and basil were at safe concentration levels. Interestingly, the majority of MICs and MFCs of *C. auris* (AR0391 and AR0395) were lower than those of *C. lusitaniae* (AR0398) and *S. cerevisiae* (AR0399). The MIC and MFC values of all essential oils except those of manuka and basil, against both strains of *C. auris*, were observed within one microdilution of each other. Essential oils of cinnamon leaf, cinnamon bark, clove bud, and lemongrass elicited the most potent antifungal activities, with cinnamon bark oil yielding the lowest MIC and MFC values against all organisms. In some cases, the MIC was below the minimum tested concentrations.

### 2.2. Gaseous-Contact Testing

Only essential oils of lemongrass, clove bud, and cinnamon bark elicited inhibition in gaseous contact (Table 5). These essential oils were also the most effective in the direct contact assay. All three of these essential oils were lethal at 100 μL. At 10 μL, lethality was observed with cinnamon bark, complete inhibition was observed with clove bud, and lemongrass elicited no effect.

### 2.3. Synergism Testing

A range of interactions were observed between essential oils and antifungal drugs (Table 6, Table 7, Table 8 and Table 9). The fractional inhibitory concentration index (FICI) values ranged from 0.0625 to 5.0. This range included at least one instance of each interaction. Antagonism was only present with the combination of amphotericin B and the essential oil of clove bud. However, clove bud also displayed synergistic activity with fluconazole against all three organisms and an additive to synergistic association with flucytosine. The essential oil of lemongrass was generally additive with all of the drugs. The only exception was an indifferent association with amphotericin B against *C. lusitaniae* and a synergistic interaction when used with micafungin against *S. cerevisiae.* The essential oil of cinnamon bark was indifferent in most cases. All drugs except amphotericin B elicited synergism with at least one essential oil, while amphotericin B was the only one to elicit antagonism. All four drugs were observed to elicit indifferent and additive interactions.

## 3. Discussion

### 3.1. Antifungal Activity of Essential Oils

The antifungal activity elicited by the tested essential oils at levels considered safe for dermal use further strengthens their potential application in microbial control. First, the antifungal activities persisted against multiple species of yeast. Because this activity occurred at concentrations considered safe for dermal use, it is reasonable to conclude essential oils may be effective for use as topical remedies for fungal infections, meriting further investigation. The essential oils might also be effective in surface disinfection formulations [39]. Future work could examine if this antifungal activity is maintained against bloodstream infections. Since only a small number of essential oils were tested here, many more with undiscovered potency could already be widely available. Gas chromatography–mass spectrometry could be used to identify potentially active compounds. This may be beneficial because individual chemicals are easier to standardize by concentration than the essential oils themselves. The specific chemical composition of essential oils can vary based on source material, processing, and storage. In many cases the primary constituents are retained across all preparations, but may vary in final concentration. The major constituents of lemongrass essential oil are neral (31.5%), citral (26.1%), and geranyl acetate (2.27%) [40]. The major constituents of cinnamon bark essential oil were found to be (*E*)-cinnamaldehyde (71.50%), linalool (7.00%), *β*-caryophyllene (6.40%), eucalyptol (5.40%), and eugenol (4.60%) [41]. The main clove bud essential oil constituents are eugenol (70–95%), eugenol acetate (up to 20%) and β-caryophyllene (12–17%) [42].

### 3.2. Gaseous Contact Activity of Essential Oils

The essential oils of cinnamon bark, lemongrass, and clove bud retained their antimicrobial activity as a gas. The remaining essential oils did not perform well at the volumes tested. This may be due to a number of factors, including insufficient dosage, incomplete volatility, or environmental conditions. The easily diffused essential oils could have applications when surface disinfections are laborious or when such disinfections are required in areas that are difficult to access. More testing is needed under more controlled conditions to evaluate gaseous concentrations, dispersal characteristics, and contact times, in order to understand the activity of the essential oils while in a gaseous state. Additional essential oils or the active compounds of the essential oils could also be tested as they may elicit antimicrobial activity when in gaseous form [33].

### 3.3. Interaction of Essential Oils and Antifungal Drugs

Several additive and synergistic combinations of essential oils and antifungal drugs were discovered. While additive combinations indicated that the two components were substitutable and thus the overall effective doses were not reduced, there are important conclusions that can be drawn. Even though the formulation dose was the same as each component when tested alone, the component doses of the essential oil and antifungal were each reduced when combined in the formulation, which may potentially reduce the side effects of each. Additionally, while the checkerboard assay is a good option for the large-scale screening of formulation efficacy, other methods exist that are more sensitive [34]. Several things must be considered when evaluating the results. First, because microdilutions are utilized, a fairly large range of concentrations is represented over a small number of wells, and thus a low resolution is present. A difference of one microdilution can have a significant effect on the FICI. To validate the results produced by the checkerboard assay, smaller dilutions should be assessed. Additionally, the methods of analysis for this type of synergism assay are still not well-defined [43]. FICI cutoffs for the interaction categories are different depending on the publication, and some even combine multiple categories [43]. Thus, a more standardized method of analysis and better-defined categories of interaction can help the use of combination therapy by allowing results to be compared on the same scales and avoid confusion when interpreting the results of other investigators.

Cinnamon bark oil elicited mostly indifferent interactions with all antifungals tested. Previous studies have suggested this essential oil acts by disrupting membrane integrity [44]. The lack of synergism is unexpected in this case, as increased membrane permeability could be assumed to facilitate increased therapeutic cellular penetration to drug targets. A further examination of the mode of action and interaction of selected essential oils with antifungal drugs will improve our understanding of these results and potentially lead to improved predictive evaluation.

In all cases except with amphotericin B, clove bud oil elicited interactions that were additive or synergistic. The primary component of clove oil, eugenol, is thought to damage the yeast cell wall and decrease membrane integrity [45]. In the case of clove oil, the essential oil allows the enhanced access of flucytosine, fluconazole, and micafungin to their respective drug targets, as with combinations of amphotericin B and other antifungals [34]. Antagonism was observed between clove oil and amphotericin. However, because both clove bud oil and amphotericin B act on the cellular envelope, there may be interference between the two agents. Eugenol has been demonstrated to bind to steroid receptors [46,47]. Sterols and steroids have been demonstrated to inhibit the activity of amphotericin B [48,49]. It is possible that eugenol behaves similarly to a sterol or steroid and thus impedes the function of amphotericin B.

Lemongrass oil elicited additive or synergistic interactions with all of the tested drugs. While there have been attempts to elucidate the mode of action of lemongrass oil, several mechanisms are involved and are dose dependent [50]. Existing research indicates that cytoplasmic leakage due to lemongrass oil appeared to be low, and the majority of the effects seem to be targeted on intercellular components [50]. One suggestion was that the lemongrass oil causes the cell to swell [50]. The increased surface area could lead to an increase in permeability. However, the primary components of lemongrass oil are citral isomers (terpenoids) [51]. Citral has been demonstrated to decrease membrane fluidity [52]. Decreased membrane fluidity has been linked to increased susceptibility to drug-resistant bacteria and *Candida* spp. [53,54].

Several potentially therapeutic combinations of essential oils and antifungal drugs have beeb discovered. Further studies are warranted to validate the results in vivo and examine if toxicity to human cells is also increased for either the antifungal drugs or essential oils when used in combination. Many other future lines of inquiry are possible. First, additional combinations could be screened for synergism. Since synergism screening is labor-intensive, these results could be used to select new combinations for screening by considering essential oils similar to those tested here and testing them in appropriately matched pairs. The active chemical components of the oils could also be examined in place of the essential oils. The pool of isolates and species of organisms could also be expanded, to see if the interactions extend beyond the small group of organisms tested here. Other, more sensitive methods of synergism screening could be pursued with the additive combinations to detect any borderline synergism. Finally, the molecular modes of action of the various combinations could be examined. Understanding these could help researchers replicate the effects in future drug development.

With the growing problem of drug-resistant fungal pathogens, new disinfectants, antiseptics, and treatment options are in constant demand. The data here demonstrate the antimicrobial activity of several essential oils against *C. auris*, a multi-drug-resistant human pathogen. In addition to having antimicrobial properties alone, some of these essential oils displayed the ability to enhance the effectiveness of antifungal drugs that are being rendered ineffective due to the increasing prevalence of drug-resistance. The effective essential oils and their respective combinations with antifungal drugs could find use as surface disinfectants and skin antiseptics. They could also be validated for use as treatment options to battle *C. auris* infections and possibly other mycoses.

## 4. Materials and Methods

### 4.1. Culture Preparation and Maintenance

Two panels of drug-resistant *Candida* species were acquired from the CDC (Table S1). The first was the Drug Resistant *Candida* Panel (species other than *C. albicans*) and the second was the *C. auris* Panel, consisting of 32 and 20 strains, respectively. The isolates were received as glycerol stocks, which were inoculated to malt extract broth and malt extract agar (MEA, Thermo Fisher Scientific, Waltham, MA, USA) and incubated at 37 °C for two days. Subcultures from colonies grown on MEA, of each organism, were preserved in 30% glycerol for long-term storage at −80 °C. The MEA plates were retained for future use and preserved by wrapping in paraffin film and storing at 4 °C. New MEA plates were inoculated for at least once a month by subculturing from a colony of an active plate. After a fifth-generation plate was made, a new series was started from a glycerol stock, which was performed to maintain wild type characteristics.

### 4.2. Working Stock Preparation

To ensure standardized starting populations and reduce preparation time for all experiments, glycerol working stocks were prepared at standardized population densities. For each isolate used, an overnight culture was grown at 30 °C while shaking. The following day, the cell concentration was diluted with sterile deionized water to 1.0 to 2.0 × 10^6^ cells mL^−1^ based on previously determined standard curves (Table S2). Then, 1.0 mL of the culture was mixed with an equal volume of 60% glycerol to yield a 2.0 mL working stock in 30% glycerol. These were stored at −80 °C and were discarded if not used within a month, to ensure viability.

### 4.3. Antifungal Activity Testing

The antifungal activities of several essential oils were tested using microdilutions. The selected essential oils were obtained from Mountain Rose Herbs (Table S3). The manufacturer’s quality control data (File S4) are included as Supplemental Materials [32]. Two isolates of *C. auris* (AR0381 and AR0385) were used, as well as one isolate each of *C. lusitaniae* (AR0388) and *S. cerevisiae* (AR0399). The base media was yeast nitrogen base supplemented with 2% glucose (YNBG, Y0626, Sigma-Aldrich, St. Louis, MO, USA). A volume of 380 μL of YNBG supplemented with 1% of the tested essential oil was added to the first well of a column on a 96-well plate. The remaining wells of the column were filled with 190 μL of YNBG with 1% dimethyl sulfoxide (DMSO). Microdilutions were performed by transferring 190 μL from the first well to the second well of the column and mixing, then transferring 190 μL from the second well to the third well and mixing, and repeating this for the entire eight-well column, with 190 μL from the last well being discarded to maintain an equal volume in each well. After the microdilutions were prepared, each well was inoculated with 10 μL of a culture from a freshly thawed working stock, for a starting population of 1.0 to 2.0 × 10^4^ cells per well. This brought the final volume of each well to 200 μL. The 96-well plates were sealed using breathable cover films (Diversified BioTech BEM-1) and incubated for 72 h at 30 °C. Following incubation, the plates were agitated to homogenize the contents of each well and the OD600 was measured using a plate reader (Biotek Synergy H1). Each microdilution series was performed in triplicate and the mean was calculated for each concentration.

Two metrics that are frequently used to assess the effectiveness of antifungals are minimum inhibitory concentration (MIC) and minimum fungicidal concentration (MFC). The MIC is the minimum concentration required to completely inhibit the growth of an inoculum. The MFC is the minimum concentration required to kill the starting inoculum. Complete inhibition was interpreted if the mean OD600 value was less than 0.5, a value at which visible growth becomes apparent, and the MIC was assigned to the minimum concentration that completely inhibited the growth of the starting population. The contents of the inhibited wells were then transferred to 1.0 mL of Letheen broth (Remel, San Diego, CA, USA), which was used to both neutralize the antimicrobial activity of the essential oil and provide a growth medium for the culture. The broth cultures were incubated for 72 h at 30 °C while shaking, after neutralization. Following incubation, the media were observed visually for signs of growth. The MFC was determined as the minimum concentration that displayed no visible growth at this phase. Negative controls were included for the essential oil, DMSO, YNBG, and Letheen broth. A positive control containing DMSO and Letheen broth was also included in each assay.

### 4.4. Antifungal Activity of Essential Oils in a Gaseous Phase

Each essential oil was tested for antifungal activity as a gas in a sealed airspace. *C. auris* (AR0385) was diluted to a concentration of 0.5 to 1.0 × 10^4^ cells mL^−1^. Then, 10 μL aliquots were transferred and spread onto four 60 mm Petri dishes containing MEA. Three of these plates were then placed with the lids removed in an empty 150 mm Petri dish. An aluminum foil well was placed at the center of the 150 mm Petri dish and a 100 μL aliquot of the tested essential oil was dispensed into it. The 150 mm Petri dish was immediately sealed with paraffin film to create an enclosed airspace. The fourth 60 mm Petri dish was independently sealed with paraffin film and used as a positive control. All plates were incubated for 72 h at 30 °C. Following incubation, the 60 mm Petri dishes were observed for growth and inhibition was determined when no visible growth was observed. The lids were then replaced on the inhibited plates, sealed with paraffin film and returned to incubation for an additional 72 h with no exposure to the essential oil. Following the second incubation period, the plates were again checked for growth. Lethality was determined if no visible signs of growth were observed. This experiment was then repeated using 10 μL of the essential oils that displayed fungicidal activity at 100 μL and using 1 μL of the essential oil diluted in 99 μL of DMSO for those that displayed fungicidal activity at 10 μL. The 1 μL was diluted to minimize the risk of evaporation before the lid could be replaced.

### 4.5. Synergism Testing

A modified checkerboard method was used to examine the interaction between the essential oils displaying the lowest MICs and select antifungal drugs. The essential oils tested were lemongrass, clove bud, and cinnamon bark. The antifungals used were amphotericin B, flucytosine, fluconazole, and micafungin. One strain each of *C. auris* (AR0381), *C. lusitaniae* (AR0398), and *S. cerevisiae* (AR0399) were used as challenge organisms. Stock solutions of the antifungals were prepared by dissolving the antifungal in DMSO to yield concentrations not exceeding the manufacturer’s recommended solubility limit for DMSO (Table 10). Working solutions were prepared for each organism by diluting the stock solutions in sterile deionized water to produce concentrations equal to 320 times the published MIC for the *Candida auris* (AR0381). Then, YNBG with 1% of a combination of DMSO and the tested essential oil was prepared.

The volume of essential oil used was calculated to yield a final solution containing 16 times the previously determined MIC for the organism. A 360 μL aliquot of this solution was added to the first well of column 1 of the 96-well plate, and 190 μL aliquots were added to the remaining wells of the column and to the first well of column 9. The remaining solution containing YNBG, essential oil, and DMSO was then diluted with an equal volume of YNBG plus 1% DMSO to yield a new solution with half the concentration of essential oil. A 360 μL aliquot of this solution was added to the first well of column 2, and 190 μL aliquots were added to the remaining wells of the column and to the second well of column 9. The process of diluting the essential oil mixture and adding it to the plate was repeated for the next six columns of the 96-well plate. A 360 μL aliquot of the YNBG plus 1% DMSO was added to the top well of column 10 and 190 μL aliquots were added to the remaining wells of the column.

After the essential oil microdilutions were performed, 20 μL aliquots of the antifungal working solution were added to the top well of each column, except for columns 9, 11 and 12. Column 9 was reserved for using only the essential oil and columns 11 and 12 were reserved for controls. A 190 μL aliquot from the top well of the first column was transferred to the second well and mixed. A 190 μL aliquot from the second well was transferred to the third and mixed. This was continued for the remainder of the wells in the column, with 190 μL from the last well being discarded to ensure an equal volume in each well of the column. These microdilutions were repeated for columns 2 through 8, as well as column 10. The final result was a grid of every tested concentration of essential oil and antifungal, as well as each concentration of essential oil and antifungal in isolation in columns 9 and 10, respectively.

**Figure 1:**
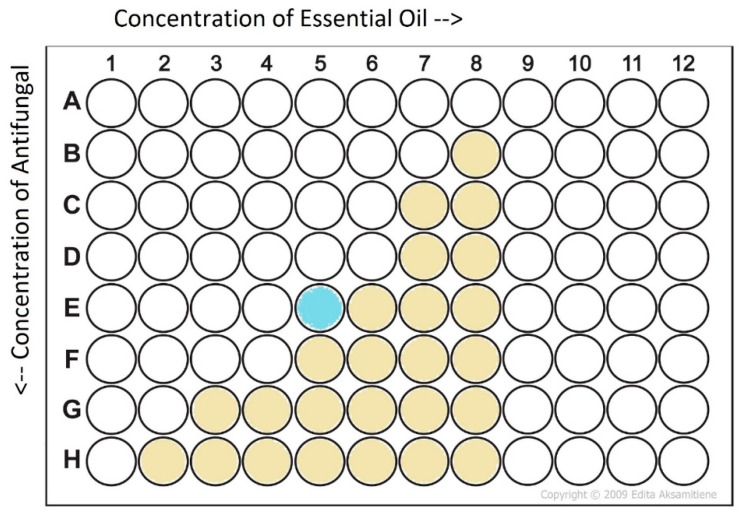
Selection of well for calculating FIC values of an example plate. Gold wells indicate growth. The well used for calculation is the inhibited well that is approximately midway along the inhibition interface, denoted as the light blue well in the example plate.

After the antifungal microdilutions were complete, 10 μL aliquots from a 1.0 to 2.0 × 10^7^ cells mL^−1^ solution of each organism were added to each test well, then plates were sealed with breathable cover films and incubated for 72 h at 30 °C. Each experiment was conducted in triplicate. Following incubation, the plates were agitated to homogenize the contents of each well and the OD600 was recorded for each well. Complete inhibition was determined when the mean OD600 for a particular combination was less than 0.5. The fractional inhibitory concentration index (FICI) of the checkerboard assays was calculated from the fraction inhibitory concentrations (FICs) as shown in Equation (1) [37]. The test well used to determine the MIC in combination was the one located most centrally along the inhibition interface (Figure 1). The interpretation of the interaction was also as previously described (Table 11) [55].

Calculation of fractional inhibitory concentration index (FICI).

(1) $$FIC\;of\;Oil=\frac{MIC\;of\;Oil\;in\;Combination}{MIC\;of\;Oil\;Alone}\;FIC\;of\;Antifungal=\frac{MIC\;of\;Antifungal\;in\;Combination}{MIC\;of\;Antifungal\;Alone}\;FICI=FIC\;of\;Oil+FIC\;of\;Antifungal$$

## Figures and Tables

**Table 1:** Global incidence of invasive fungal infections reported in 2017 [3].

Infection	Annual Incidence	Incidence per 100,000 People
Invasive Candidiasis	~750,000	10.00
Invasive Aspergillosis	~300,000	4.00
*Pneumocystis jirovecii* pneumonia	~500,000	6.67
Cryptococcosis in AIDS	~223,000	2.97
Mucormycosis	>10,000	0.13
Disseminated histoplasmosis	~100,000	1.33

**Table 2:** Centers for Disease Control and Prevention AR Bank isolates and corresponding origin and clade designations of *Candida auris*.

Isolate	Origin	Clade
0381	East Asia	II
0382	South Asia	I
0383	Africa	III
0384	Africa	III
0385	South America	IV
0386	South America	IV
0387	South Asia	I
0388	South Asia	I
0389	South Asia	I
0390	South Asia	I

**Table 3:** MIC by percentage (*v*/*v*) of select essential oils against *C. auris* (AR0381, AR0385), *C. lusitaniae* (AR0398), and *S. cerevisiae* (AR0399). Tests were conducted in triplicate. Coloration is based on inhibitory concentrations in *C. auris.* Green: MIC < 0.1%; yellow: MIC < 1.0%; red: MIC ≥ 1.0% or not detected.

Essential Oil	AR0381	AR0385	AR0398	AR0399
Tea Tree	0.25%	0.13%	0.50%	0.50%
Geranium	0.13%	0.06%	0.50%	0.25%
Lime Peel	0.25%	0.13%	1.0%	>1.0%
Eucalyptus	>1.0%	>1.0%	>1.0%	>1.0%
Peppermint	0.25%	0.25%	1.0%	1.0%
Manuka	0.25%	1%	>1.0%	1.0%
Clove Bud	0.01%	0.02%	0.06%	0.25%
Myrrh	0.13%	0.13%	1.0%	1.0%
Spearmint	0.13%	0.06%	0.50%	>1.0%
Cinnamon Leaf	<0.01%	<0.01%	0.13%	0.25%
Cinnamon Bark	<0.01%	<0.01%	<0.01%	<0.01%
Bergamot	0.25%	0.13%	>1.0%	>1.0%
Lemon	>1.0%	>1.0%	>1.0%	>1.0%
Frankincense	1.0%	1.0%	>1.0%	>1.0%
Coriander	0.50%	0.50%	1.0%	1.0%
Bitter Orange	>1.0%	>1.0%	>1.0%	>1.0%
Grapefruit	>1.0%	>1.0%	>1.0%	>1.0%
Lavender	1.0%	1.0%	1.0%	>1.0%
Ginger	1.0%	>1.0%	>1.0%	>1.0%
Basil	0.13%	0.50%	>1.0%	>1.0%
Lemongrass	0.02%	0.03%	0.13%	0.25%

**Table 4:** MFC of select essential oils against *C. auris* (AR0381, AR0385), *C. lusitaniae* (AR0398), and *S. cerevisiae* (AR0399). Coloration is based on fungicidal concentrations in *C. auris.* Green: MFC < 0.1%; yellow: MFC < 1.0%; red: MFC ≥ 1.0% or not detected (ND).

Essential Oil	AR0381	AR0385	AR0398	AR0399
Tea Tree	0.50%	1.0%	ND	1.0%
Geranium	0.50%	0.25%	1.0%	0.50%
Peppermint	1.0%	1.0%	ND	ND
Clove Bud	0.06%	0.13%	ND	ND
Cinnamon Leaf	0.50%	0.25%	0.50%	0.50%
Cinnamon Bark	0.02%	0.02%	0.01%	0.02%
Bergamot	0.50%	0.25%	ND	ND
Coriander	1.0%	1.0%	1.0%	ND
Lavender	1.0%	ND	1.0%	ND
Basil	1.0%	1.0%	ND	ND
Lemongrass	0.13%	0.06%	1.0%	0.50%

**Table 5:** Antifungal activity of essential oils in a gaseous phase against *C. auris* (AR0385). N = 3, volumes of essential oils are 100 μL unless stated otherwise.

Essential Oil	Result	Essential Oil	Result
Tea Tree	No inhibition	Cinnamon Bark (1.0 μL)	No inhibition
Geranium	No inhibition	Bergamot	No inhibition
Lime Peel	No inhibition	Lemon	No inhibition
Eucalyptus	No inhibition	Frankincense	No inhibition
Peppermint	No inhibition	Coriander	No inhibition
Manuka	No inhibition	Bitter Orange	No inhibition
Clove Bud (100 μL)	Fungicidal	Grapefruit	No inhibition
Clove Bud (10 μL)	Inhibitory	Lavender	No inhibition
Myrrh	No inhibition	Ginger	No inhibition
Spearmint	No inhibition	Basil	No inhibition
Cinnamon Leaf	No inhibition	Lemongrass (100 μL)	Fungicidal
Cinnamon Bark (100 μL)	Fungicidal	Lemongrass (10 μL)	No inhibition
Cinnamon Bark (10 μL)	Fungicidal		

**Table 6:** Interactions between select antifungal drugs and essential oils against *C. auris* (AR0381). Colors based on interpretation. Green: synergistic; yellow: additive or indifferent; red: antagonistic.

Antifungal	Essential Oil	FIC_AF_	FIC_EO_	FICI	Interpretation
Micafungin	Cinnamon Bark	1	1	2	Indifferent
Micafungin	Clove Bud	1	1	2	Indifferent
Micafungin	Lemongrass	0.5	0.125	0.625	Additive
Flucytosine	Cinnamon Bark	0.5	0.25	0.75	Additive
Flucytosine	Clove Bud	0.0625	0.125	0.1875	Synergistic
Flucytosine	Lemongrass	0.125	0.5	0.625	Additive
Amphotericin B	Cinnamon Bark	1	0.5	1.5	Indifferent
Amphotericin B	Clove Bud	4	0.5	4.5	Antagonistic
Amphotericin B	Lemongrass	0.25	0.5	0.75	Additive
Fluconazole	Cinnamon Bark	1	0.5	1.5	Indifferent
Fluconazole	Clove Bud	0.03125	0.25	0.28125	Synergistic
Fluconazole	Lemongrass	0.125	0.5	0.625	Additive

**Table 7:** Interactions between select antifungal drugs and essential oils against *C. lusitaniae* (AR0398). Colors based on interpretation. Green: synergistic; yellow: additive or indifferent; red: antagonistic.

Antifungal	Essential Oil	FIC_AF_	FIC_EO_	FICI	Interpretation
Micafungin	Cinnamon Bark	1	1	2	Indifferent
Micafungin	Clove Bud	0.5	1	1.5	Indifferent
Micafungin	Lemongrass	0.125	0.5	0.625	Additive
Flucytosine	Cinnamon Bark	1	0.5	1.5	Indifferent
Flucytosine	Clove Bud	0.25	0.5	0.75	Additive
Flucytosine	Lemongrass	0.5	0.5	1	Additive
Amphotericin B	Cinnamon Bark	1	0.5	1.5	Indifferent
Amphotericin B	Clove Bud	4	1	5	Antagonistic
Amphotericin B	Lemongrass	1	0.25	1.25	Indifferent
Fluconazole	Cinnamon Bark	2	0.5	2.5	Indifferent
Fluconazole	Clove Bud	0.03125	0.03125	0.0625	Synergistic
Fluconazole	Lemongrass	0.25	0.5	0.75	Additive

**Table 8:** Interactions between select antifungal drugs and essential oils against *S. cerevisiae* (AR0399). Colors based on interpretation. Green: synergistic; yellow: additive or indifferent; red: antagonistic.

Antifungal	Essential Oil	FIC_AF_	FIC_EO_	FICI	Interpretation
Micafungin	Cinnamon Bark	1	1	2	Indifferent
Micafungin	Clove Bud	0.5	0.5	1	Additive
Micafungin	Lemongrass	0.25	0.125	0.375	Synergistic
Flucytosine	Cinnamon Bark	1	0.75	1.75	Indifferent
Flucytosine	Clove Bud	0.25	0.5	0.75	Additive
Flucytosine	Lemongrass	0.5	0.5	1	Additive
Amphotericin B	Cinnamon Bark	1	0.5	1.5	Indifferent
Amphotericin B	Clove Bud	4	1	5	Antagonistic
Amphotericin B	Lemongrass	0.25	0.5	0.75	Additive
Fluconazole	Cinnamon Bark	1	0.5	1.5	Indifferent
Fluconazole	Clove Bud	0.25	0.25	0.5	Synergistic
Fluconazole	Lemongrass	0.125	0.5	0.625	Additive

**Table 9:** Summary of essential oil–antifungal drug interactions. Colors based on interpretation. Green: synergistic or additive–synergistic; yellow: additive or indifferent; red: antagonistic.

	Cinnamon Bark	Clove Bud	Lemongrass
**Micafungin**	Indifferent	Indifferent–Additive	Additive–Synergistic
**Flucytosine**	Indifferent–Additive	Additive–Synergistic	Additive
**Amphotericin B**	Indifferent	Antagonistic	Indifferent–Additive
**Fluconazole**	Indifferent	Synergistic	Additive

**Table 10:** Preparation of antifungal drugs and maximum concentrations of antifungal drugs and essential oils used in synergism testing. NA: Not applicable.

	Stock Solution Concentration (mg/mL)	Working Solution Concentration (μg/mL)	Maximum Concentration in Synergism Testing
**Antifungal Drug**	**------------------**	**------------------**	**(μg/mL)**
Micafungin	10.0	40.0	2.0
Amphotericin B	30.0	120.0	6.0
Flucytosine	0.2	40.0	2.0
Fluconazole	5.0	1200.0	60.0
**Essential Oil**	**------------------**	**------------------**	**%(*v*/*v*)**
Cinnamon Bark	NA	NA	0.04%
Lemongrass	NA	NA	0.32%
Clove	NA	NA	0.16%

**Table 11:** Interpretation of fractional inhibitory concentration index.

FICI Value	Interpretation
FICI ≤ 0.5	Synergistic
0.5 < FICI ≤ 1.0	Additive
1.0 < FICI ≤ 4.0	Indifferent
FICI > 4.0	Antagonistic

## Data Availability

Not applicable.

## Supplementary Materials

The following are available online at https://www.mdpi.com/article/10.3390/pathogens11080821/s1, Table S1: CDC AR Bank Isolate Identity and Minimum Inhibitory Concentrations of Isolates Used, Table S2: Essential oils, botanical source, and reported maximum safe dermal concentration, Table S3: Essential oils used in this study, File S4: EO Manufacturer QA reports.
