# Supplementary material for: Antifungal Activity of Select Essential Oils against Candida auris and Their Interactions with Antifungal Drugs
Source: Pathogens. 2022 Jul 22;11(8):821. doi: 10.3390/pathogens11080821 (PMC9331469; doi:10.3390/pathogens11080821)
Supplement: Supplementary file 1 [file pathogens-11-00821-s001.zip › pathogens-1799642-supplementary-tables.pdf]

.

Table S1 – CDC AR Bank Isolate Identity and Minimum Inhibitory Concentrations of Isolates Used.

AR Bank Reference Number.	Organism Name.	Amphotericin B.	Anidulafungin.	Caspofungin.	Fluconazole.	Flucytosine.	Isavuconazole.	Itraconazole.	Micafungin.	Posaconazole.	Voriconazole..
0381.	*Candida auris.*	0.38.	0.25.	0.125.	4.	2.	#N/A.	0.125.	0.125.	0.06.	0.03..
0382.	*Candida auris.*	0.38.	0.25.	0.5.	16.	0.125.	#N/A.	1.	0.25.	0.5.	0.5..
0383.	*Candida auris.*	0.38.	1.	0.25.	128.	0.5.	#N/A.	0.5.	1.	0.5.	4..
0384.	*Candida auris.*	0.5.	2.	16.	128.	0.5.	#N/A.	1.	2.	0.5.	1..
0385.	*Candida auris.*	0.5.	1.	0.5.	>256.	0.5.	#N/A.	1.	0.5.	1.	16..
0386.	*Candida auris.*	0.5.	1.	0.5.	>256.	0.5.	#N/A.	0.5.	0.25.	0.5.	16..
0387.	*Candida auris.*	0.75.	0.5.	0.25.	8.	8.	#N/A.	0.5.	0.5.	0.25.	0.06..
0388.	*Candida auris.*	1.5.	0.5.	1.	>256.	0.125.	#N/A.	0.5.	0.125.	0.25.	2..
0389.	*Candida auris.*	4.	1.	0.5.	256.	128.	#N/A.	0.25.	0.25.	0.125.	4..
0390.	*Candida auris.*	4.	1.	0.5.	>256.	128.	#N/A.	1.	0.25.	0.5.	8..
0391.	*Candida duobushaemulonii.*	#N/A.	0.06.	0.03.	8.	<0.125.	#N/A.	0.5.	0.125.	0.25.	0.25..
0392.	*Candida duobushaemulonii.*	#N/A.	0.06.	0.03.	8.	<0.125.	#N/A.	0.5.	0.125.	0.25.	0.06..
0393.	*Candida haemulonii.*	#N/A.	0.25.	0.125.	0.5.	<0.125.	#N/A.	0.125.	0.25.	0.125.	0.008..
0394.	*Candida duobushaemulonii.*	#N/A.	0.03.	0.008.	4.	<0.125.	#N/A.	0.06.	0.06.	0.016.	0.125..
0395.	*Candida haemulonii.*	#N/A.	#N/A.	#N/A.	#N/A.	#N/A.	#N/A.	#N/A.	#N/A.	#N/A.	#N/A..
0396.	*Kodamaea ohmeri.*	#N/A.	1.	0.25.	2.	0.5.	#N/A.	0.125.	0.5.	0.06.	0.03..
0397.	*Candida krusei.*	#N/A.	0.03.	0.125.	64.	2.	#N/A.	1.	0.125.	1.	1..
0398.	*Candida lusitaniae.*	0.38.	0.125.	0.125.	1.	<0.125.	#N/A.	0.125.	0.125.	0.5.	0.016..
0399.	*Saccharomyces cerevisiae.*	#N/A.	0.125.	0.06.	2.	<0.125.	#N/A.	0.06.	0.25.	0.5.	0.03..
0400.	*Saccharomyces cerevisiae.*	0.032.	0.5.	0.5.	1.	<0.125.	#N/A.	0.5.	0.5.	0.5.	0.06..
0314*.	*Candida glabrata.*	0.38.	1.	0.5.	64.	<0.12.	#N/A.	>16.	1.	16.	4..
0315*.	*Candida glabrata.*	0.38.	2.	16.	4.	>256.	#N/A.	1.	4.	1.	0.25..
0316*.	*Candida glabrata.*	0.05.	1.	1.	4.	<0.12.	#N/A.	0.25.	0.25.	0.25.	0.06..
0317*.	*Candida glabrata.*	0.19.	0.5.	1.	32.	<0.12.	#N/A.	1.	0.25.	1.	0.5..
0318*.	*Candida glabrata.*	0.19.	4.	16.	32.	<0.12.	#N/A.	1.	4.	1.	1..

**0319*.**	***Candida glabrata.***	**0.125.**	**0.5.**	**1.**	**4.**	**<0.12.**	**#N/A.**	**0.5.**	**2.**	**0.25.**	**0.12..**
**0320*.**	***Candida glabrata.***	**0.19.**	**0.5.**	**1.**	**4.**	**<0.12.**	**#N/A.**	**1.**	**0.25.**	**1.**	**0.12..**
**0321*.**	***Candida glabrata.***	**0.09.**	**2.**	**4.**	**64.**	**<0.12.**	**#N/A.**	**1.**	**1.**	**2.**	**2..**
**0322*.**	***Candida glabrata.***	**0.19.**	**2.**	**2.**	**8.**	**<0.12.**	**#N/A.**	**0.5.**	**0.25.**	**0.5.**	**0.12..**
**0323*.**	***Candida glabrata.***	**0.19.**	**4.**	**16.**	**4.**	**<0.12.**	**#N/A.**	**0.25.**	**4.**	**0.25.**	**0.06..**
**0324*.**	***Candida glabrata.***	**0.25.**	**4.**	**16.**	**8.**	**0.12.**	**#N/A.**	**0.5.**	**2.**	**0.5.**	**0.25..**
**0325.**	***Candida glabrata.***	**0.38.**	**4.**	**>16.**	**128.**	**<0.12.**	**#N/A.**	**16.**	**4.**	**8.**	**16..**
**0326*.**	***Candida glabrata.***	**0.016.**	**0.06.**	**0.06.**	**4.**	**<0.12.**	**#N/A.**	**1.**	**0.015.**	**1.**	**0.125..**
**0327*.**	***Candida glabrata.***	**0.25.**	**0.125.**	**0.125.**	**16.**	**0.25.**	**#N/A.**	**1.**	**0.015.**	**1.**	**0.25..**
**0328*.**	***Candida glabrata.***	**0.25.**	**0.03.**	**0.03.**	**8.**	**<0.12.**	**#N/A.**	**0.5.**	**0.015.**	**0.5.**	**0.25..**
**0329*.**	***Candida glabrata.***	**0.19.**	**0.06.**	**0.06.**	**8.**	**<0.12.**	**#N/A.**	**1.**	**0.03.**	**1.**	**0.25..**
**0330*.**	***Candida glabrata.***	**0.19.**	**0.03.**	**0.06.**	**8.**	**<0.12.**	**#N/A.**	**1.**	**0.015.**	**1.**	**0.25..**
**0331*.**	***Candida glabrata.***	**0.25.**	**0.03.**	**0.06.**	**64.**	**<0.12.**	**#N/A.**	**2.**	**0.015.**	**2.**	**1..**
**0332*.**	***Candida glabrata.***	**0.125.**	**0.06.**	**0.06.**	**128.**	**<0.12.**	**#N/A.**	**4.**	**0.015.**	**2.**	**4..**
**0333.**	***Candida glabrata.***	**0.125.**	**0.06.**	**0.06.**	**64.**	**<0.12.**	**#N/A.**	**1.**	**0.03.**	**2.**	**1..**
**0334*.**	***Candida glabrata.***	**0.125.**	**0.06.**	**0.06.**	**128.**	**<0.12.**	**#N/A.**	**>16.**	**0.03.**	**>16.**	**4..**
**0335.**	***Candida parapsilosis.***	**0.19.**	**4.**	**0.5.**	**16.**	**0.125.**	**#N/A.**	**0.5.**	**1.**	**0.25.**	**1..**
**0336*.**	***Candida parapsilosis.***	**0.047.**	**1.**	**0.25.**	**32.**	**<0.12.**	**#N/A.**	**0.125.**	**1.**	**0.125.**	**1..**
**0337*.**	***Candida parapsilosis.***	**0.094.**	**1.**	**0.25.**	**64.**	**<0.12.**	**#N/A.**	**0.125.**	**0.5.**	**0.125.**	**1..**
**0338*.**	***Candida parapsilosis.***	**0.125.**	**1.**	**1.**	**16.**	**<0.12.**	**#N/A.**	**0.5.**	**1.**	**0.25.**	**0.25..**
**0339*.**	***Candida parapsilosis.***	**0.047.**	**1.**	**0.25.**	**32.**	**0.25.**	**#N/A.**	**0.25.**	**1.**	**0.25.**	**0.5..**
**0340*.**	***Candida parapsilosis.***	**0.03.**	**2.**	**0.25.**	**0.05.**	**0.125.**	**#N/A.**	**0.125.**	**0.5.**	**0.125.**	**0.015..**
**0341*.**	***Candida parapsilosis.***	**0.023.**	**1.**	**0.5.**	**0.5.**	**0.125.**	**#N/A.**	**0.06.**	**1.**	**0.06.**	**0.015..**
**0342*.**	***Candida parapsilosis.***	**0.023.**	**2.**	**0.5.**	**0.5.**	**0.125.**	**#N/A.**	**0.06.**	**2.**	**0.06.**	**0.015..**
**0343*.**	***Candida parapsilosis.***	**0.06.**	**0.5.**	**0.25.**	**1.**	**0.125.**	**#N/A.**	**0.125.**	**1.**	**0.125.**	**0.06..**
**0344.**	***Candida parapsilosis.***	**0.06.**	**2.**	**0.25.**	**0.25.**	**<0.12.**	**#N/A.**	**0.03.**	**1.**	**0.06.**	**0.015..**
**0345.**	***Candida tropicalis.***	**0.38.**	**0.06.**	**0.06.**	**>256.**	**<0.12.**	**#N/A.**	**>16.**	**0.06.**	**>16.**	**16..**
**NRRL-Y12983.**	***Candida albicans.***	**#N/A.**	**#N/A.**	**#N/A.**	**#N/A.**	**#N/A.**	**#N/A.**	**#N/A.**	**#N/A.**	**#N/A.**	**#N/A..**
***Isolate #0381-0400 from Candida auris panel, Isolate # 0323-0345 from Drug Resistant Candida Species panel, adapted from* [28]..**											
****Not used in data presented, but included for completeness..***											

Table S2 – Essential oils, botanical source, and reported maximum safe dermal concentration.

Essential Oil	Species	MSDC(%)*
Basil	*Osimum basilicum*	0.10
Bergamot	*Citrus bergamium*	0.40
Bitter Orange	*Citrus aurantium*	1.25
Cinnamon Bark	*Cinnamomum zeylancium*	0.07
Cinnamon Leaf	*Cinnamomum zeylancium*	0.60
Clove Bud	*Syzygium aromaticum*	0.50
Coriander	*Coriandrum sativum*	NA
Eucalyptus	*Eucalyptus globulus*	20.00
Frankincense	*Boswellia carteri*	NA
Geranium	*Pelargonium graveolens*	17.50
Ginger	*Zingiber officinale*	NA
Grapefruit	*Citrus paradisi*	4.00
Lavender	*Lavandula angustifolia*	0.10
Lemon	*Citrus limon*	2.00
Lemongrass	*Cymbopogon flexuosus*	0.70
Lime Peel	*Citrus aurantifolia*	0.70
Manuka	*Leptospermum scoparium*	NA
Myrrh	*Commiphora myrrha*	NA
Peppermint	*Mentha piperita*	5.40
Spearmint	*Mentha spicata*	1.70
Tea Tree	*Melaleuca alterenifolia*	15.00

**MSDC: Maximum Safe Dermal Concentration, taken from* [34]

Table S3 – Essential Oils used in this study.

Essential Oil	Manufacturer	Item Number
Coriander Seed Essential Oil	Mountain Rose Herbs	eo_co1/2
Ginger Essential Oil	Mountain Rose Herbs	eo_gi1/2
Eucalyptus Essential Oil	Mountain Rose Herbs	eo_eu1/2
Geranium Essential Oil	Mountain Rose Herbs	eo_ge1/2
Tea Tree Essential Oil	Mountain Rose Herbs	eo_tto1/2
Peppermint Essential Oil	Mountain Rose Herbs	eo_pep1/2
Lemongrass Essential Oil	Mountain Rose Herbs	eo_lg1/2
Cinnamon Bark Essential Oil	Mountain Rose Herbs	eo_cb1/2
Orange, Bitter Essential Oil	Mountain Rose Herbs	eo_or_b1/2
Grapefruit Essential Oil	Mountain Rose Herbs	eo_gr1/2
Clove Bud Essential Oil	Mountain Rose Herbs	eo_clo1/2
Spearmint Essential Oil	Mountain Rose Herbs	eo_sp1/2
Basil Essential Oil	Mountain Rose Herbs	eo_ba1/2
Bergamot Essential Oil	Mountain Rose Herbs	eo_be1/2
Cinnamon Leaf Essential Oil	Mountain Rose Herbs	eo_cl1/2
Frankincense Essential Oil	Mountain Rose Herbs	eo_fr1/2
Lavender Essential Oil	Mountain Rose Herbs	eo_la_o1/2
Lemon Essential Oil	Mountain Rose Herbs	eo_le1/2
Lime Peel Essential Oil	Mountain Rose Herbs	eo_lp1/2

Manuka Essential Oil	Mountain Rose Herbs	eo_manu1/2
Myrrh Essential Oil	Mountain Rose Herbs	eo_mro1/2
