# Supplementary material for: Antifungal Activity of Select Essential Oils against Candida auris and Their Interactions with Antifungal Drugs
Source: Pathogens. 2022 Jul 22;11(8):821. doi: 10.3390/pathogens11080821 (PMC9331469; doi:10.3390/pathogens11080821)
Supplement: Supplementary file 1 [file pathogens-11-00821-s001.zip › S4/Basil EO- EO2487.pdf]

mountain rose herbs®
.com

A Herbs, Health and Harmony Company.
Since 1987

CERTIFICATE OF ANALYSIS

Mailing: PO Box 50220 / Eugene, Oregon 97405
Phone: 800-879-3337 / Fax 510-217-4012
E-mail: lab@mountainroseherbs.com
www.mountainroseherbs.com

Product Name: Basil Essential Oil
Botanical Name: *Ocimum basilicum*
Chemotype: *Methyl chavicol*
Origin: India
Manufacture Date: November 2017
Part Used: Flowering plant
Lot Number: EO2487
Extraction: Distillation
Grade: Certified Organic
Additives: None

Test	Results
Appearance	Translucent, almost colorless
Odor	Fresh, warm, spicy, herbaceous
Refractive Index @20°C	1.5055*
Specific Gravity @20°C	0.942*
Optical Rotation @20°C	-6.50°*

*By Vendor Report# 170760

3/14/18

Christine Rice / Steven Yeager | **Date**
Quality Control Department

This information is presented in good faith and was compiled through testing methods in our laboratory, and with the assistance of our suppliers, harvesters, and processors information. We make no warranty, either expressed or implied in the complete accuracy of the information listed herein. The data in this analysis is offered solely for your verification and consideration. It is the responsibility of the buyer to provide themselves with up to date analyses for any botanicals purchased through Mountain Rose Herbs.
