# Supplementary material for: Antifungal Activity of Select Essential Oils against Candida auris and Their Interactions with Antifungal Drugs
Source: Pathogens. 2022 Jul 22;11(8):821. doi: 10.3390/pathogens11080821 (PMC9331469; doi:10.3390/pathogens11080821)
Supplement: Supplementary file 1 [file pathogens-11-00821-s001.zip › S4/Clove Bud EO GCMS- EO2934.pdf]

Clove Bud Essential Oil- EO2934

alpha-Cubebene	0.09
Furfural	0.05
α- Copaene	0.20
β- Caryophyllene	7.96
α- Humulene	0.61
δ- Cadinene	0.22
Methyl Salicylate	0.13
trans-Calamenene	0.08
Caryophyllene Oxide	0.15
Methyl Eugenol ether	0.03
Caryophyllenyl Alcohol	0.06
Eugenol	82.17
Eugenol Acetate	7.37
Chavicol	0.19
