# Supplementary material for: Antifungal Activity of Select Essential Oils against Candida auris and Their Interactions with Antifungal Drugs
Source: Pathogens. 2022 Jul 22;11(8):821. doi: 10.3390/pathogens11080821 (PMC9331469; doi:10.3390/pathogens11080821)
Supplement: Supplementary file 1 [file pathogens-11-00821-s001.zip › S4/Coriander Seed EO- EO2851.pdf]

mountain rose herbs ®
.com

An Herbs, Health, and Harmony Company.
Since 1987

CERTIFICATE OF ANALYSIS

Mailing: PO Box 50220 / Eugene, Oregon 97405
Phone: 800-879-3337 / Fax 510-217-4012
E-mail: qc@mountainroseherbs.com
www.mountainroseherbs.com

Product Name: Coriander Seed Essential Oil
Botanical Name: *Coriandrum sativum*
Origin: Hungary
Manufacture Date: November 2019
Part Used: Seed
Lot Number: EO2851
Extraction: Distillation
Grade: Certified Organic
Additives: None
Notes: None

Test	Results
Appearance	Light yellow
Odor	Sweet, spicy, woody, slight fruity top note

Rachelle Theobald 6/12/20
Christine Rice / Rachelle Theobald — Date
Quality Control Department

This information is presented in good faith and was compiled through testing methods in our laboratory, and with the assistance of our suppliers, harvesters, and processors information. We make no warranty, either expressed or implied in the complete accuracy of the information listed herein. The data in this analysis is offered solely for your verification and consideration. It is the responsibility of the buyer to provide themselves with up to date analyses for any botanicals purchased through Mountain Rose Herbs.

rk
