# Supplementary material for: Antifungal Activity of Select Essential Oils against Candida auris and Their Interactions with Antifungal Drugs
Source: Pathogens. 2022 Jul 22;11(8):821. doi: 10.3390/pathogens11080821 (PMC9331469; doi:10.3390/pathogens11080821)
Supplement: Supplementary file 1 [file pathogens-11-00821-s001.zip › S4/Eucalyptus GCMS- EO2835.pdf]

Date : May 29, 2020

Certificate of Analysis – GC Profiling

Sample identification

Internal code : 20E19-MRH01
Customer identification : Eucalyptus - China - EO2835
Type : Essential oil
Source : *Eucalyptus globulus*
Customer : Mountain Rose Herbs

Analysis

Method: PC-MAT-007 - Analysis of the composition of an essential oil or other volatile liquide by FAST GC-FID (in French); identifications validated by GC-MS.
Analyst : Fanny Charlier, B. Sc.
Analysis date : May 20, 2020

Checked and approved by :

Alexis St-Gelais, M. Sc., chimiste 2013-174

Notes: This report may not be published, including online, without the written consent from Laboratoire PhytoChemia. This report is digitally signed, it is only considered valid if the digital signature is intact. The results only describe the samples that were submitted to the assays.

Laboratoire PhytoChemia
628 Boulevard du Saguenay Ouest, Saguenay (Qc) G7J 1H4 | www.phytochemia.com

Physicochemical data

Physical aspect: Clear liquid
Refractive index: 1.4605 ± 0.0003 (20 °C; method PC-MAT-016)

Conclusion

No adulterant, contaminant or diluent has been detected using this method.

ANALYSIS SUMMARY – CONSOLIDATED CONTENTS

New readers of similar reports are encouraged to read table footnotes at least once.

Identification	%	Class
Isovaleral	tr	Aliphatic aldehyde
Isoamyl alcohol	0.01	Aliphatic alcohol
Hashishene	0.01	Monoterpene
α-Thujene	0.03	Monoterpene
α-Pinene	4.80	Monoterpene
Camphene	0.04	Monoterpene
α-Fenchene	0.02	Monoterpene
Thuja-2,4(10)-diene	0.01	Monoterpene
β-Pinene	0.36	Monoterpene
trans-Dehydroxylinalool oxide	0.02	Monoterpenic ether
Myrcene	0.60	Monoterpene
α-Phellandrene	1.22	Monoterpene
Δ3-Carene	0.07	Monoterpene
α-Terpinene	0.30	Monoterpene
para-Cymene	0.75	Monoterpene
Limonene	2.94	Monoterpene
1,8-Cineole	82.88	Monoterpenic ether
β-Phellandrene	3.95	Monoterpene
(*Z*)-β-Ocimene	0.09	Monoterpene
(*E*)-β-Ocimene	0.06	Monoterpene
γ-Terpinene	1.62	Monoterpene
Unknown	0.01	Oxygenated monoterpene
meta-Mentha-4,6-dien-8-ol	0.01	Monoterpenic alcohol
Terpinen-4-ol	tr	Monoterpenic alcohol
α-Terpineol	0.01	Monoterpenic alcohol
Unknown	0.02	Unknown
α-Gurjunene	0.01	Sesquiterpene
(*trans*?)-6-Hydroxypiperitone	0.01	Monoterpenic alcohol
β-Caryophyllene	0.01	Sesquiterpene
γ-Cadinene	0.03	Sesquiterpene
δ-Cadinene	0.02	Sesquiterpene
trans-Cadina-1,4-diene	0.03	Sesquiterpene
Aromadendrene	0.01	Sesquiterpene
**Consolidated total**	**99.94%**	

tr: The compound has been detected below 0.005% of total signal.

Note: no correction factor was applied

About "consolidated" data: The table above presents the breakdown of the sample volatile constituents after applying an algorithm to collapse data acquired from the multi-columns system of PhytoChemia into a single set of consolidated contents. In case of discrepancies between columns, the algorithm is set to prioritize data from the most standard DB-5 column, and smallest values so as to avoid overestimating individual content. This process is semi-automatic. Advanced users are invited to consult the “Full analysis data” table after the chromatograms in this report to access the full untreated data and perform their own calculations if needed.

Unknowns: Unknown compounds’ mass spectral data is presented in the “Full analysis data” table. The occurrence of unknown compounds is to be expected in many samples, and does not denote particular problems unless noted otherwise in the conclusion.

This page was intentionally left blank. The following pages present the complete data of the analysis.

628 Boulevard du Saguenay, Saguenay (Qc) G7J 1H4 | www.phytochemia.com

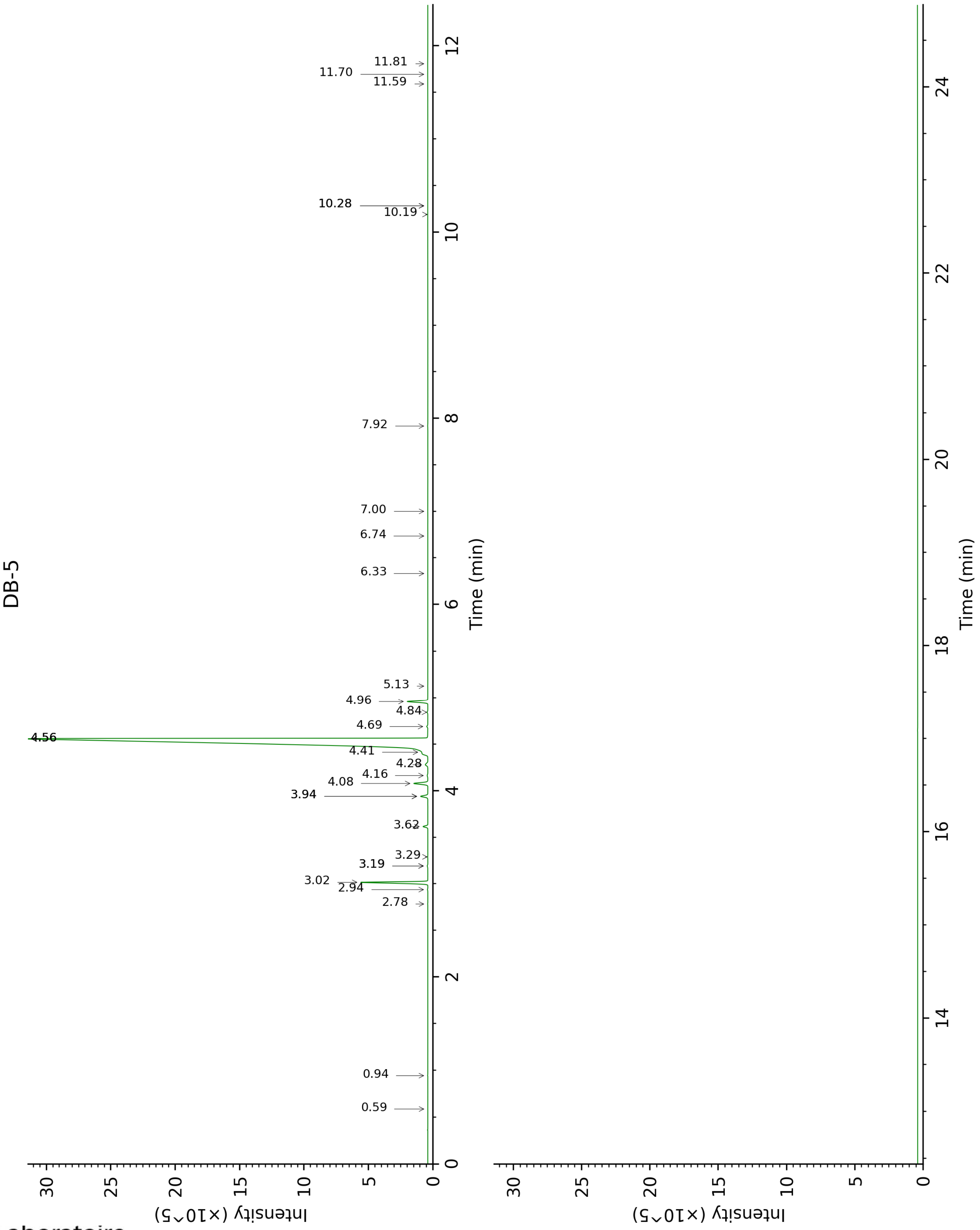

Laboratoire PhytoChemia

Plus que des analyses... des conseils

628 Boulevard du Saguenay, Saguenay (Qc) G7J 1H4 | www.phytochemia.com

DB-WAX

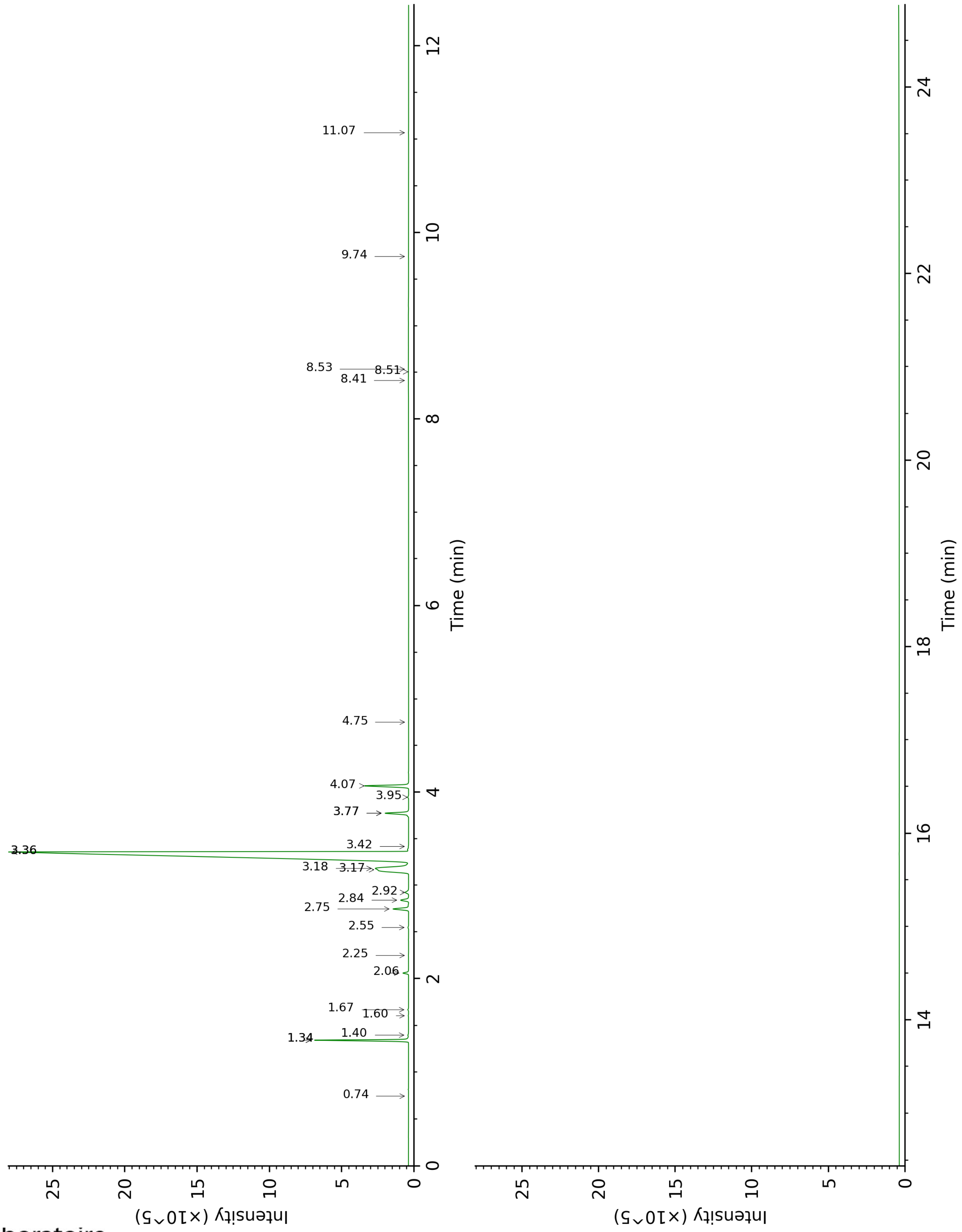
Time (min)
Intensity (×10^5)
0.74
1.34
1.40
1.67
1.60
2.06
2.25
2.55
2.75
2.84
2.92
3.18
3.17
3.36
3.42
3.77
3.95
4.07
4.75
8.53
8.51
8.41
9.74
11.07

FULL ANALYSIS DATA

Identification	Column DB-5			Column DB-WAX		
	R.T	R.I	%	R.T	R.I	%
Isovaleral	0.59	640	tr	0.74	885	tr
Isoamyl alcohol	0.94	737	0.01	3.42	1177	0.02
Hashishene	2.78	915	0.01	1.34*	990	4.74
α-Thujene	2.94	925	0.03	1.40	998	0.04
α-Pinene	3.02	930	4.80	1.34*	990	[4.74]
Camphene	3.20*	942	0.05	1.67	1025	0.04
α-Fenchene	3.20*	942	[0.05]	1.60	1018	0.02
Thuja-2,4(10)-diene	3.29	948	0.01	2.25	1082	0.02
β-Pinene	3.62	970	0.36	2.06	1064	0.36
trans-Dehydroxylinalool oxide	3.94*	991	0.62	3.36*	1172	80.23
Myrcene	3.94*	991	[0.62]	2.84	1131	0.60
α-Phellandrene	4.08	1001	1.22	2.75	1124	1.20
Δ3-Carene	4.16	1006	0.07	2.55	1108	0.04
α-Terpinene	4.28	1013	0.30	2.92	1138	0.31
para-Cymene	4.41	1022	0.75	4.07	1226	3.16
Limonene	4.56*	1031	89.77	3.17	1157	2.94
1,8-Cineole	4.56*	1031	[89.77]	3.36*	1172	[80.23]
β-Phellandrene	4.56*	1031	[89.77]	3.18	1158	3.95
(*Z*)-β-Ocimene	4.69	1039	0.09	3.78*	1204	1.77
(*E*)-β-Ocimene	4.84	1049	0.06	3.95	1217	0.06
γ-Terpinene	4.96	1056	1.62	3.78*	1204	[1.77]
Unknown [m/z 79, 93 (60), 43 (40), 94 (35), 137 (33), 77 (26), 91 (20), 152 (18)]	5.13	1067	0.01	4.75	1276	0.01
meta-Mentha-4,6-dien-8-ol	6.33	1144	0.01			
Terpinen-4-ol	6.74	1170	tr	8.53	1552	0.01
α-Terpineol	7.00	1188	0.01	9.74	1648	0.01
Unknown [m/z 43, 97 (69), 107 (46), 41 (28), 55 (21), 109 (20)...]	7.92	1250	0.02	11.07	1758	0.02
α-Gurjunene	10.19	1404	0.01			
(*trans*?)-6-Hydroxypiperitone	10.28*	1411	0.02			
β-Caryophyllene	10.28*	1411	[0.02]	8.41	1543	0.01
γ-Cadinene	11.59	1509	0.03			
δ-Cadinene	11.70	1517	0.02			
trans-Cadina-1,4-diene	11.81	1526	0.03			
Aromadendrene				8.51	1550	0.01
**Total identified**		**99.90%**			**99.54%**	
**Total reported**		**99.92%**			**99.56%**	

*: Two or more compounds are coeluting on this column
[xx]: Duplicate percentage due to coelutions, not taken into account in the consolidated total
†: Peaks apexes were resolved, but peaks overlapped and were summed for analysis
tr: The compound has been detected below 0.005% of total signal.

Note: no correction factor was applied
R.T.: Retention time (minutes)
R.I.: Retention index

628 Boulevard du Saguenay, Saguenay (Qc) G7J 1H4 | www.phytochemia.com
