# Supplementary material for: Antifungal Activity of Select Essential Oils against Candida auris and Their Interactions with Antifungal Drugs
Source: Pathogens. 2022 Jul 22;11(8):821. doi: 10.3390/pathogens11080821 (PMC9331469; doi:10.3390/pathogens11080821)
Supplement: Supplementary file 1 [file pathogens-11-00821-s001.zip › S4/Geranium EO GCMS- EO2950.pdf]

Geranium EO- EO2950

			normes/norms	résultat/results
ASPECT / APPEARANCE<br>*CQ-ME-01-16*	LIQUIDE LIMPIDE / LIMPID LIQUID		LIQUIDE LIMPIDE	Conforme / Conform
COULEUR / COLOR<br>*CQ-ME-01-16*	JAUNE PALE / PALE YELLOW		JAUNE PALE A JAUNE FONCE A VERDATRE PARFOIS	Conforme / Conform
ODEUR / ODOR<br>*CQ-ME-01-16*	CONFORME / CONFORM		ROSEE, HERBACEE	Conforme / Conform
DENSITE (20°) / SPECIFIC GRAVITY 20°C<br>*CQ-ME-01-23*	0.897	G/CM3	0,885 - 0,905	Conforme / Conform
INDICE REFRACTION (20°) / REFRACTIVE INDEX 20°C<br>*CQ-ME-01-08*	1.466		1,461 - 1,475	Conforme / Conform
POUVOIR ROTATOIRE / SPECIFIC OPTICAL ROTATION<br>*CQ-ME-01-05*	-11.4	DEG	-14 - -8	Conforme / Conform
SOLUBILITE ALCOOL 70° / ALCOHOL SOLUBILITY 70°<br>*CQ-ME-01-27*	2	VOL	<= 3	Conforme / Conform
10 EPI GAMMA EUDESMOL<br>*CQ-ME-01-64*	4.1	%	3 - 6	Conforme / Conform
ALPHA TERPINEOL<br>*CQ-ME-01-64*	0.3	%	0,3 - 0,6	Conforme / Conform
CIS ROSE OXYDE<br>*CQ-ME-01-64*	1.1	%	0,7 - 1,5	Conforme / Conform
CITRONNELLOL<br>*CQ-ME-01-64*	32.7	%	25 - 36	Conforme / Conform
FORMIATE DE CITRONELLYLE<br>*CQ-ME-01-64*	7.1	%	5 - 8	Conforme / Conform
LINALOL<br>*CQ-ME-01-64*	4.8	%	4 - 8,5	Conforme / Conform
PHENYL ETHYL TIGLATE<br>*CQ-ME-01-64*	0.7	%	0,5 - 1,2	Conforme / Conform
TRANS ROSE OXYDE<br>*CQ-ME-01-64*	0.4	%	0,3 - 0,6	Conforme / Conform

Geranium EO- EO2950

GERANIOL	11.6	%	10 - 18	Conforme / Conform
CQ-ME-01-64				
GERANYLE BUTYRATE	1	%	0,7 - 2	Conforme / Conform
CQ-ME-01-64				
GERANYLE FORMIATE	2.7	%	2 - 7	Conforme / Conform
CQ-ME-01-64				
GERANYLE TIGLATE	1	%	0,9 - 2	Conforme / Conform
CQ-ME-01-64				
GUAIA-6-9-DIENE	0.3	%	<= 0,5	Conforme / Conform
CQ-ME-01-64				
ISOMENTHONE	6.3	%	4 - 8	Conforme / Conform
CQ-ME-01-64				
ANALYSE CPG / GC ANALYSIS	CONFORME / CONFORM			Conforme / Conform
CQ-ME-01-64				

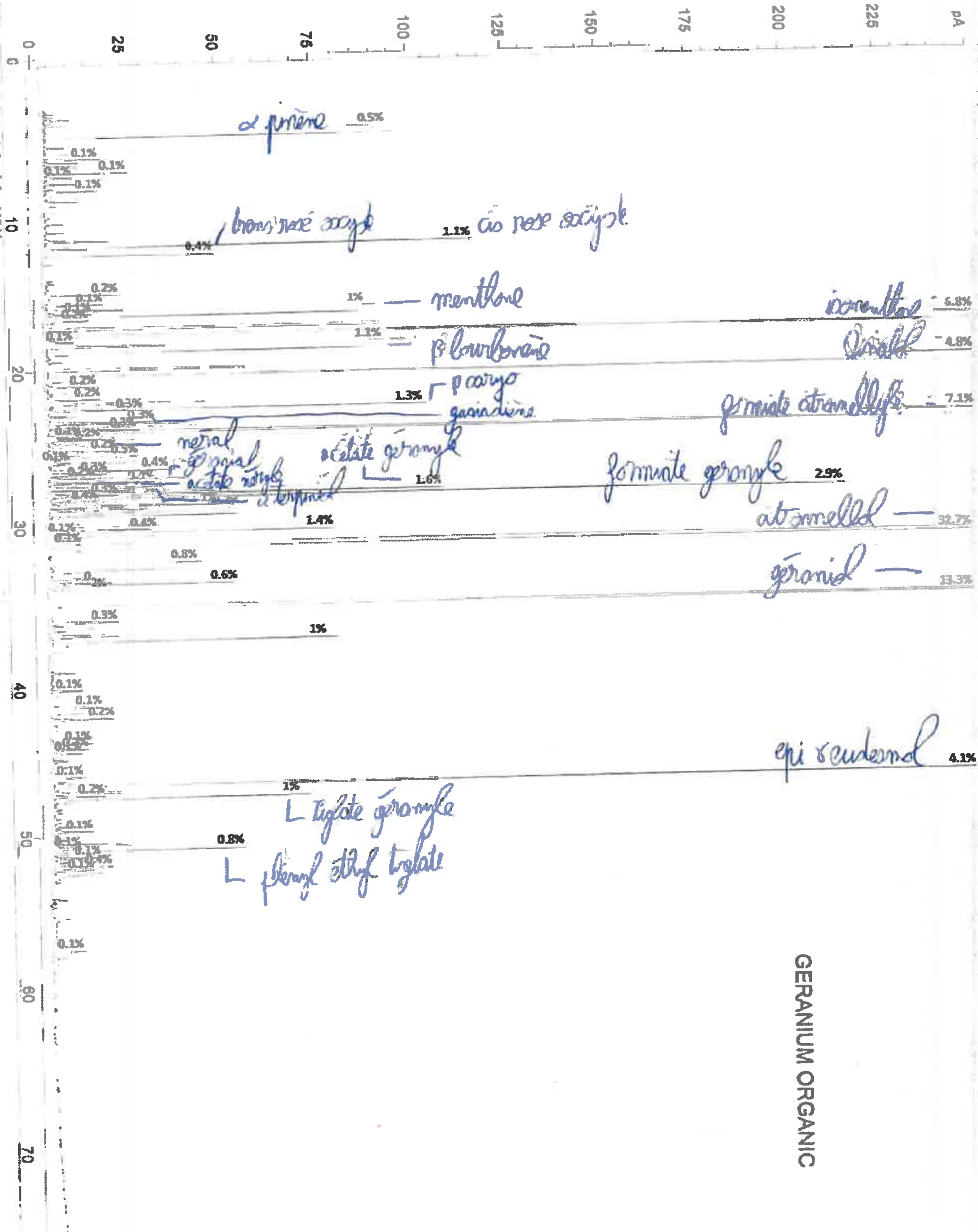

GERANIUM ORGANIC
α pinène 0.5%
cis rose oxyde 1.1%
menthone 1%
isomenthone 6.8%
β bourbonène 1.1%
β caryo
géranial
formiate géranyle 2.9%
citronellol 32.7%
géraniol 13.3%
epi γ eudesmol 4.1%
tiglate géranyle 1%
phényl éthyl tiglate 0.8%

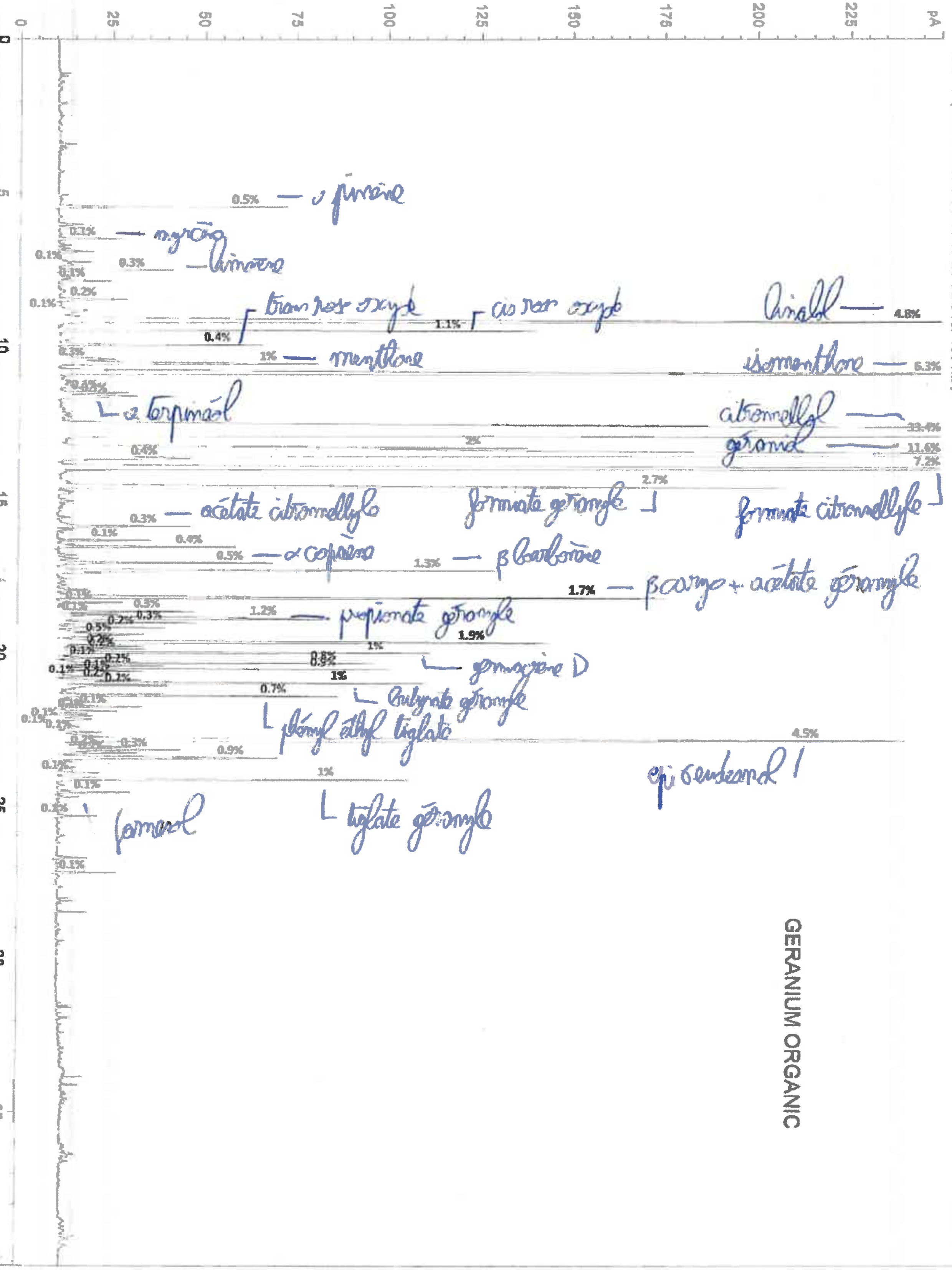
GERANIUM ORGANIC
α pinène
myrcène
limonène
trans rose oxyde
cis rose oxyde
linalol
menthone
isomenthone
α terpinéol
citronellol
géraniol
acétate citronellyle
formiate géranyle
formiate citronellyle
α copaène
β bourbonène
β caryo + acétate géranyle
propionate géranyle
germacrène D
butyrate géranyle
phényl éthyl tiglate
épi eudesmol !
farnesol
tiglate géranyle
