# Supplementary material for: Antifungal Activity of Select Essential Oils against Candida auris and Their Interactions with Antifungal Drugs
Source: Pathogens. 2022 Jul 22;11(8):821. doi: 10.3390/pathogens11080821 (PMC9331469; doi:10.3390/pathogens11080821)
Supplement: Supplementary file 1 [file pathogens-11-00821-s001.zip › S4/Lavender EO- EO2918.pdf]

mountain rose herbs.com

CERTIFICATE OF ANALYSIS

Mailing: PO Box 50220 / Eugene, Oregon 97405
Phone: 800-879-3337 / Fax 510-217-4012
E-mail: qc@mountainroseherbs.com
www.mountainroseherbs.com

Product Name: Lavender Essential Oil
Botanical Name: *Lavandula angustifolia*
Origin: Bulgaria
Production Date: December 2017
Part Used: Flowering Top
Lot Number: EO2918
Extraction: Distillation
Grade: Certified Organic
Additives: N/A

Test	Specifications	Results	Method
Identity	Passed	Passed	Organoleptic

Rachelle Theobald   11/20/20

Steven Yeager / Rachelle Theobald / Geri Green   **Date**
Quality Control Department

This information is presented in good faith and was compiled through testing methods in our laboratory, contracted laboratories, and with the assistance of our suppliers, harvesters, and processors information. We make no warranty, either expressed or implied in the complete accuracy of the information listed herein. The data in this analysis is offered solely for your verification and consideration. It is the responsibility of the buyer to provide themselves with up to date analyses for any botanicals purchased through Mountain Rose Herbs.

smt
