# Supplementary material for: Antifungal Activity of Select Essential Oils against Candida auris and Their Interactions with Antifungal Drugs
Source: Pathogens. 2022 Jul 22;11(8):821. doi: 10.3390/pathogens11080821 (PMC9331469; doi:10.3390/pathogens11080821)
Supplement: Supplementary file 1 [file pathogens-11-00821-s001.zip › S4/Lemon EO GCMS- EO2879.pdf]

Lemon Essential Oil
lot #Eo2879

α- Pinene	2.49
Camphene	0.06
β-Pinene	12.65
Sabinene	2.02
β-Myrcene	1.53
α- Phellandrene	0.03
α-Terpinene	0.18
Limonene	66.56
β-Phellandrene	0.36
cis-beta-Ocimene	0.04
γ- Terpinene	9.41
para-Cymene	0.25
Terpinolene	0.38
6-methyl-5-hepten-2-one	0.05
Nonanal	0.07
Citronellal	0.07
Decanal	0.02
Linalool	0.09
cis-α- Bergamotene	0.20
β-Caryophyllene	0.11
Neral	0.55
α-Terpineol	0.11
Neryl Acetate	0.47
β- Bisabolene	0.48
Geranial	1.26
Geranyl Acetate	0.37
Geraniol	0.02
