# Supplementary material for: Antifungal Activity of Select Essential Oils against Candida auris and Their Interactions with Antifungal Drugs
Source: Pathogens. 2022 Jul 22;11(8):821. doi: 10.3390/pathogens11080821 (PMC9331469; doi:10.3390/pathogens11080821)
Supplement: Supplementary file 1 [file pathogens-11-00821-s001.zip › S4/Lemongrass EO GCMS- EO2823.pdf]

Date : April 22, 2020

Certificate of Analysis – GC Profiling

Sample identification

Internal code : 20D15-MRH01
Customer identification : Lemongrass - Sri Lanka - EO2823
Type : Essential oil
Source : *Cymbopogon flexuosus*
Customer : Mountain Rose Herbs

Analysis

Method: PC-MAT-007 - Analysis of the composition of an essential oil or other volatile liquide by FAST GC-FID (in French); identifications validated by GC-MS.
Analyst : Fanny Charlier, B. Sc.
Analysis date : April 21, 2020

Checked and approved by :

Alexis St-Gelais, M. Sc., chimiste 2013-174

Notes: This report may not be published, including online, without the written consent from Laboratoire PhytoChemia. This report is digitally signed, it is only considered valid if the digital signature is intact. The results only describe the samples that were submitted to the assays.

Laboratoire PhytoChemia
628 Boulevard du Saguenay Ouest, Saguenay (Qc) G7J 1H4 | www.phytochemia.com

Physicochemical data

Physical aspect: Yellow liquid
Refractive index: 1.4853 ± 0.0003 (20 °C; method PC-MAT-016)

Conclusion

No adulterant, contaminant or diluent has been detected using this method.

628 Boulevard du Saguenay, Saguenay (Qc) G7J 1H4 | www.phytochemia.com

ANALYSIS SUMMARY – CONSOLIDATED CONTENTS

New readers of similar reports are encouraged to read table footnotes at least once.

Identification	%	Classe
Ethanol	tr	Aliphatic alcohol
Acetone	0.02	Aliphatic ketone
Isovaleral	tr	Aliphatic aldehyde
2-Methylbutyral	tr	Aliphatic aldehyde
2-Ethylfuran	tr	Furan
Isoamyl alcohol	tr	Aliphatic alcohol
(3*Z*)-Hexenol	0.01	Aliphatic alcohol
Tricyclene	0.12	Monoterpene
α-Pinene	0.16	Monoterpene
Camphene	0.94	Monoterpene
α-Fenchene	0.02	Monoterpene
Thuja-2,4(10)-diene	0.01	Monoterpene
β-Pinene	tr	Monoterpene
Sabinene	0.02	Monoterpene
6-Methyl-5-hepten-2-one	0.88	Aliphatic ketone
Myrcene	0.06	Monoterpene
6-Methyl-5-hepten-2-ol	0.06	Aliphatic alcohol
Octanal	0.10	Aliphatic aldehyde
α-Terpinene	0.01	Monoterpene
para-Cymene	0.01	Monoterpene
Limonene	0.24	Monoterpene
1,8-Cineole	0.03	Monoterpenic ether
Benzeneacetaldehyde	0.01	Simple phenolic
(*Z*)-β-Ocimene	0.27	Monoterpene
(*E*)-β-Ocimene	0.16	Monoterpene
2,6-Dimethyl-5-heptenal (melonal)	0.02	Aliphatic aldehyde
γ-Terpinene	0.01	Monoterpene
cis-Linalool oxide (fur.)	0.01	Monoterpenic alcohol
4-Nonanone	1.10	Aliphatic ketone
Camphenilone	0.02	Normonoterpenic ketone
Terpinolene	0.04	Monoterpene
trans-Linalool oxide (fur.)	0.01	Monoterpenic alcohol
4-Nonanol	0.02	Aliphatic alcohol
Rosefuran	0.12	Monoterpenic ether
Perillene	0.10	Monoterpenic ether
Linalool	0.98	Monoterpenic alcohol
cis-Chrysanthemal?	0.03	Monoterpenic aldehyde
(*Z*)-6-Methyl-3,5-heptadien-2-one	0.03	Aliphatic ketone
trans-para-Mentha-2,8-dien-1-ol	0.03	Monoterpenic alcohol
Unknown	0.16	Unknown
exo-Isocitral	0.01	Monoterpenic aldehyde
trans-Chrysanthemal	0.37	Monoterpenic aldehyde
Citronellal	1.48	Monoterpenic aldehyde
Borneol	0.17	Monoterpenic alcohol
Isoneral	0.73	Monoterpenic aldehyde
α-Phellandren-8-ol	0.17	Monoterpenic alcohol
Rosefuran oxide	0.06	Monoterpenic ether

628 Boulevard du Saguenay, Saguenay (Qc) G7J 1H4 | www.phytochemia.com

Terpinen-4-ol	0.14	Monoterpenic alcohol
Unknown	0.09	Oxygenated monoterpene
Isogeranial	1.16	Monoterpenic aldehyde
Unknown	0.17	Unknown
α-Terpineol	0.20	Monoterpenic alcohol
Myrtenal	0.01	Monoterpenic aldehyde
trans-Isopiperitenol	0.02	Monoterpenic alcohol
Unknown	0.04	Oxygenated monoterpene
Decanal	0.19	Aliphatic aldehyde
cis-Isopiperitenol	0.02	Monoterpenic alcohol
2,3-Epoxyneral?	0.05	Monoterpenic aldehyde
Nerol	0.03	Monoterpenic alcohol
Citronellol	0.19	Monoterpenic alcohol
Neral	31.47	Monoterpenic aldehyde
Geraniol	5.91	Monoterpenic alcohol
Geranial	41.34	Monoterpenic aldehyde
Unknown	0.08	Oxygenated monoterpene
Geranyl formate	0.07	Monoterpenic ester
Unknown	0.03	Unknown
Neric acid	0.09	Monoterpenic acid
α-Cubebene	0.01	Sesquiterpene
Citronellyl acetate	0.06	Monoterpenic ester
Cyclosativene I	0.07	Sesquiterpene
Cyclosativene II	0.09	Sesquiterpene
Neryl acetate	0.09	Monoterpenic ester
Geranic acid	0.18	Aliphatic acid
α-Copaene	0.09	Sesquiterpene
β-Bourbonene	0.03	Sesquiterpene
Geranyl acetate	2.67	Monoterpenic ester
β-Cubebene	0.04	Sesquiterpene
β-Elemene	0.07	Sesquiterpene
Longifolene	0.03	Sesquiterpene
β-Caryophyllene	1.19	Sesquiterpene
β-Copaene	0.03	Sesquiterpene
trans-α-Bergamotene	0.01	Sesquiterpene
α-Humulene	0.14	Sesquiterpene
(*E*)-Isoeugenol	0.27	Phenylpropanoid
cis-Muurola-4(15),5-diene	0.04	Sesquiterpene
trans-Cadina-1(6),4-diene	0.05	Sesquiterpene
Germacrene D	0.15	Sesquiterpene
γ-Amorphene	0.02	Sesquiterpene
epi-Cubebol	0.10	Sesquiterpenic alcohol
α-Muurolene	0.04	Sesquiterpene
δ-Amorphene	0.02	Sesquiterpene
γ-Cadinene	1.06	Sesquiterpene
Cubebol	0.21	Sesquiterpenic alcohol
δ-Cadinene	0.24	Sesquiterpene
10-epi-Cubebol?	0.03	Sesquiterpenic alcohol
(*E*)-γ-Bisabolene	0.15	Sesquiterpene
Neryl butyrate	0.04	Monoterpenic ester
α-Elemol	0.04	Sesquiterpenic alcohol
Germacrene B	0.03	Sesquiterpene

628 Boulevard du Saguenay, Saguenay (Qc) G7J 1H4 | www.phytochemia.com

Geranyl butyrate	0.11	Monoterpenic ester
Caryophyllene oxide	0.46	Sesquiterpenic ether
Humulene epoxide II	0.04	Sesquiterpenic ether
Selin-6-en-4α-ol isomer	0.02	Sesquiterpenic alcohol
1-epi-Cubenol	0.02	Sesquiterpenic alcohol
Cubenol	0.03	Sesquiterpenic alcohol
β-Eudesmol	0.01	Sesquiterpenic alcohol
α-Eudesmol	0.01	Sesquiterpenic alcohol
(2*Z*,6*Z*)-Farnesol	0.02	Sesquiterpenic alcohol
Farnesal isomer	0.03	Sesquiterpenic aldehyde
(2*E*,6*E*)-Farnesal	0.01	Sesquiterpenic aldehyde
Neophytadiene	0.01	Diterpene
meta-Camphorene	0.01	Diterpene
Unknown	0.04	Unknown
Dicitral	0.03	Diterpenic aldehyde
Phytol isomer	0.05	Diterpenic alcohol
Unknown	0.02	Unknown
Unknown	tr	Unknown
Unknown	0.01	Unknown
Unknown	0.01	Unknown
**Consolidated total**	**98.36%**	

tr: The compound has been detected below 0.005% of total signal.

Note: no correction factor was applied

About "consolidated" data: The table above presents the breakdown of the sample volatile constituents after applying an algorithm to collapse data acquired from the multi-columns system of PhytoChemia into a single set of consolidated contents. In case of discrepancies between columns, the algorithm is set to prioritize data from the most standard DB-5 column, and smallest values so as to avoid overestimating individual content. This process is semi-automatic. Advanced users are invited to consult the "Full analysis data" table after the chromatograms in this report to access the full untreated data and perform their own calculations if needed.

Unknowns: Unknown compounds' mass spectral data is presented in the "Full analysis data" table. The occurrence of unknown compounds is to be expected in many samples, and does not denote particular problems unless noted otherwise in the conclusion.

628 Boulevard du Saguenay, Saguenay (Qc) G7J 1H4 | www.phytochemia.com

This page was intentionally left blank. The following pages present the complete data of the analysis.

628 Boulevard du Saguenay, Saguenay (Qc) G7J 1H4 | www.phytochemia.com

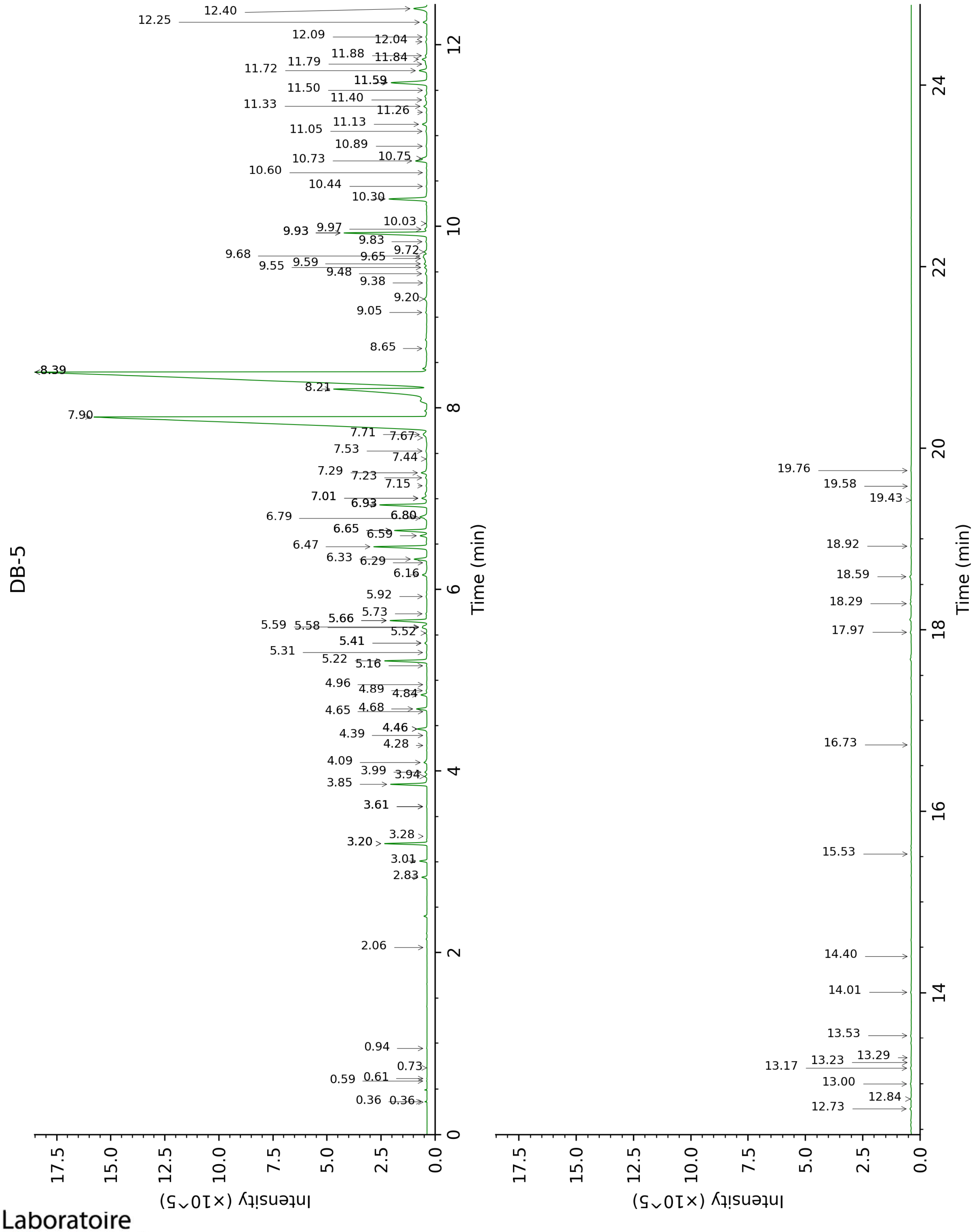
DB-5
Time (min)
Intensity (×10^5)
Time (min)
Intensity (×10^5)

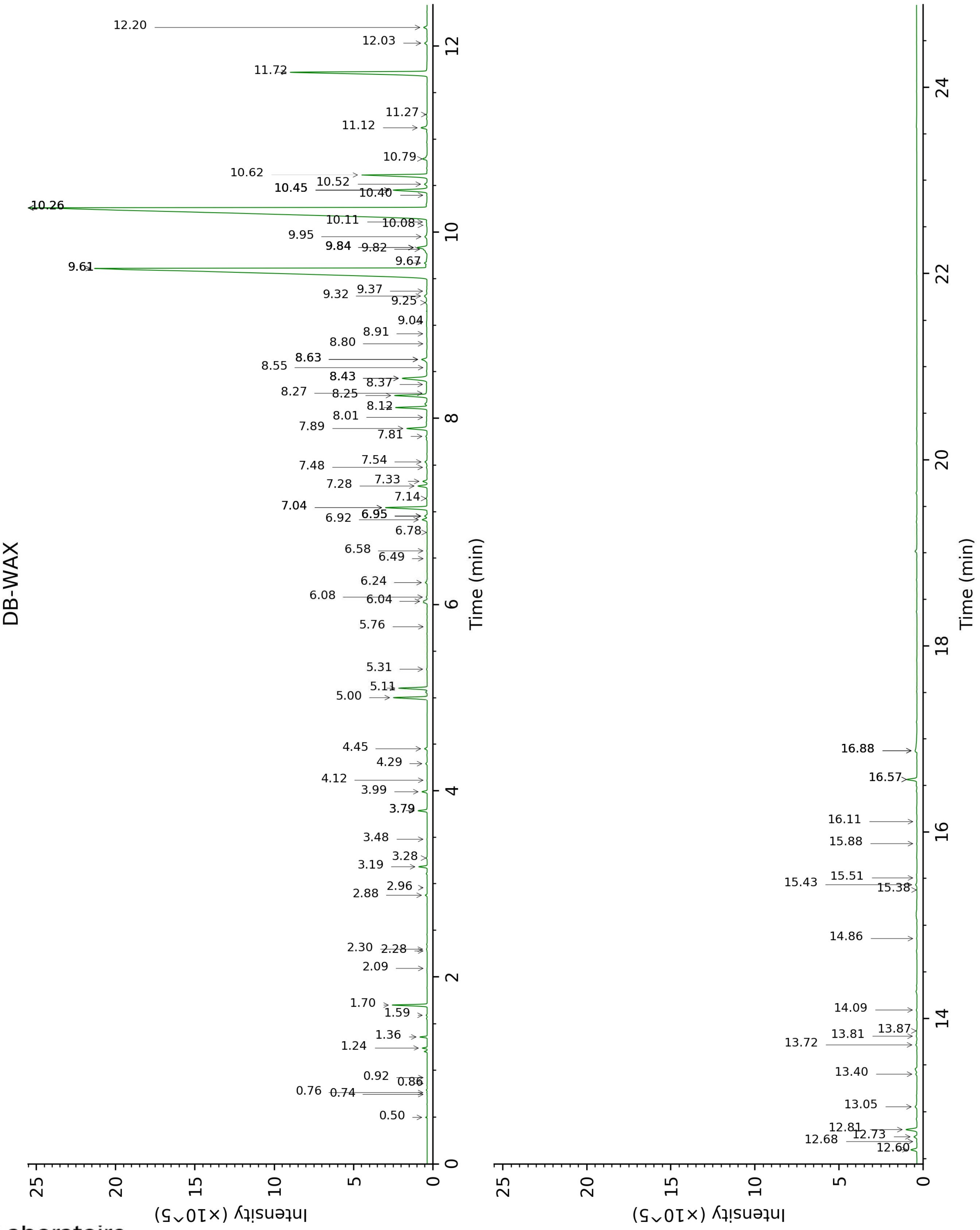

Laboratoire PhytoChemia

Plus que des analyses... des conseils

628 Boulevard du Saguenay, Saguenay (Qc) G7J 1H4 | www.phytochemia.com

FULL ANALYSIS DATA

Identification	Column DB-5			Column DB-WAX		
	**R.T**	**R.I**	**%**	**R.T**	**R.I**	**%**
Ethanol	0.36	522	tr	0.86	909	tr
Acetone	0.36	522	0.02	0.50	786	0.02
Isovaleral	0.59	640	tr	0.76	890	0.01
2-Methylbutyral	0.62	652	tr	0.74	882	tr
2-Ethylfuran	0.73	700	tr	0.92	919	tr
Isoamyl alcohol	0.94	737	tr	3.48	1179	0.01
(3*Z*)-Hexenol	2.06	857	0.01	5.76	1343	0.01
Tricyclene	2.83	918	0.12	1.24	972	0.11
α-Pinene	3.01	930	0.16	1.36	991	0.16
Camphene	3.20*	942	0.94	1.70	1026	0.94
α-Fenchene	3.20*	942	[0.94]	1.59	1016	0.02
Thuja-2,4(10)-diene	3.28	948	0.01	2.28	1084	tr
β-Pinene	3.61*	969	0.02	2.09	1065	tr
Sabinene	3.61*	969	[0.02]	2.30	1086	0.02
6-Methyl-5-hepten-2-one	3.86	986	0.88	5.11	1296	0.87
Myrcene	3.94	991	0.06	2.88	1132	0.05
6-Methyl-5-hepten-2-ol	3.99	994	0.06	6.95*	1430	0.09
Octanal	4.10	1002	0.10	4.45	1249	0.08
α-Terpinene	4.28	1013	0.01	2.96	1138	0.01
para-Cymene	4.39	1020	0.01	4.12	1225	0.01
Limonene	4.46*	1025	0.27	3.19	1156	0.24
1,8-Cineole	4.46*	1025	[0.27]	3.28	1163	0.03
Benzeneacetaldehyde	4.65	1037	0.01	8.91	1578	0.01
(*Z*)-β-Ocimene	4.68	1039	0.27	3.79*	1202	0.27
(*E*)-β-Ocimene	4.84	1048	0.16	3.99	1216	0.15
2,6-Dimethyl-5-heptenal (melonal)	4.89	1052	0.02	5.31	1311	0.02
γ-Terpinene	4.96	1056	0.01	3.79*	1202	[0.27]
cis-Linalool oxide (fur.)	5.16	1069	0.01	6.58	1402	0.01
4-Nonanone	5.22	1072	1.10	5.00	1289	1.11
Camphenilone	5.31	1078	0.02	6.49	1396	0.01
Terpinolene	5.41*	1085	0.06	4.29	1238	0.04
trans-Linalool oxide (fur.)	5.41*	1085	[0.06]	6.95*	1430	[0.09]
4-Nonanol	5.52	1092	0.02			
Rosefuran	5.58	1096	0.12	6.04	1363	0.10
Perillene	5.59	1096	0.10	6.24	1377	0.06
Linalool	5.66*	1101	1.02	8.12	1516	0.98
cis-Chrysanthemal?	5.66*	1101	[1.02]	6.08	1366	0.03
(*Z*)-6-Methyl-3,5-heptadien-2-one	5.73	1106	0.03	8.27	1528	0.06
trans-para-Mentha-2,8-dien-1-ol	5.92	1118	0.03	9.04	1587	0.03
Unknown [m/z 81, 70 (98), 67 (63), 82 (53),	6.16	1133	0.16	6.92	1427	0.19

41 (46), 69 (46), 109 (43)...]						
exo-Isocitral	6.30	1142	0.01	7.54	1472	0.08
trans-Chrysanthemal	6.34	1145	0.37	7.28	1453	0.31
Citronellal	6.47	1154	1.48	7.04*	1436	1.47
Borneol	6.59	1162	0.17	9.84*	1652	0.39
Isoneral	6.65*	1165	0.90	7.89	1499	0.73
α-Phellandren-8-ol	6.65*	1165	[0.90]	10.26*	1686	41.30
Rosefuran oxide	6.79	1174	0.06	8.63*†	1556	0.24
Terpinen-4-ol	6.80*	1175	0.24	8.63*†	1556	[0.24]
Unknown [m/z 84, 83 (74), 137 (56), 41 (47), 93 (43), 108 (40)... 152 (2)]	6.80*	1175	[0.24]	9.67	1638	0.09
Isogeranial	6.94*	1184	1.33	8.25	1526	1.16
Unknown [m/z 69, 41 (65), 109 (36), 67 (16), 84 (11), 43 (10), 55 (9)...]	6.94*	1184	[1.33]			
α-Terpineol	7.01*†	1189	0.22	9.84*	1652	[0.39]
Myrtenal	7.01*†	1189	[0.22]	8.80	1569	0.01
trans-Isopiperitenol	7.15	1198	0.02	10.45*	1702	1.35
Unknown [m/z 84, 41 (83), 83 (79), 91 (76), 93 (67), 119 (64), 137 (63), 109 (54), 108 (54)... 152 (4)]	7.23	1204	0.04			
Decanal	7.29	1207	0.19	7.33	1457	0.14
cis-Isopiperitenol	7.44	1218	0.02	10.40	1697	0.01
2,3-Epoxyneral?	7.53	1224	0.05			
Nerol	7.67	1234	0.03	11.12	1758	0.23
Citronellol	7.71	1236	0.19	10.79	1730	0.16
Neral	7.90	1250	31.47	9.61*	1633	31.26
Geraniol	8.21	1271	5.91	11.72	1809	6.00
Geranial	8.40*	1284	41.42	10.26*	1686	[41.30]
Unknown [m/z 43, 69 (77), 41 (70), 109 (54)... 152 (6)]	8.40*	1284	[41.42]	13.05	1928	0.08
Geranyl formate	8.66	1302	0.07	9.95	1661	0.12
Unknown [m/z 82, 59 (44), 41 (43), 95 (31), 43 (29), 81 (24)...]	9.06	1332	0.03	12.74	1899	0.11
Neric acid	9.20	1336	0.09	16.57*	2272	0.36
α-Cubebene	9.38	1348	0.01	6.78	1416	0.01
Citronellyl acetate	9.48	1355	0.06	9.61*	1633	[31.26]
Cyclosativene I	9.55	1360	0.07	6.95*	1430	[0.09]
Cyclosativene II	9.59	1363	0.09	7.04*	1436	[1.47]
Neryl acetate	9.65	1367	0.09	10.26*	1686	[41.30]
Geranic acid	9.68	1369	0.18	16.88*	2305	0.23
α-Copaene	9.72	1372	0.09	7.14	1444	0.04
β-Bourbonene	9.83	1380	0.03	7.48	1468	0.03
Geranyl acetate	9.93*	1387	2.66	10.62	1715	2.67

β-Cubebene	9.93*	1387	[2.66]	7.81	1493	0.04
β-Elemene	9.97	1390	0.07	8.43*	1540	1.19
Longifolene	10.03	1394	0.03	8.01	1508	0.03
β-Caryophyllene	10.30	1414	1.19	8.43*	1540	[1.19]
β-Copaene	10.44	1424	0.03	8.37	1536	0.04
trans-α-Bergamotene	10.60	1435	0.01	8.55	1549	0.01
α-Humulene	10.73†	1445	0.47	9.32	1610	0.14
(*E*)-Isoeugenol	10.75†	1447	[0.47]	16.57*	2272	[0.36]
cis-Muurola-4(15),5-diene	10.89	1457	0.04	9.37	1614	0.02
trans-Cadina-1(6),4-diene	11.05	1469	0.05	9.25	1604	0.06
Germacrene D	11.13	1475	0.15	9.82	1650	0.10
γ-Amorphene	11.26	1485	0.02	9.84*	1652	[0.39]
epi-Cubebol	11.33	1490	0.10	12.03	1837	0.10
α-Muurolene	11.40	1495	0.04	10.11	1674	0.04
δ-Amorphene	11.50	1503	0.02	10.08	1671	0.02
γ-Cadinene	11.59*	1509	1.26	10.45*	1702	[1.35]
Cubebol	11.59*	1509	[1.26]	12.60	1886	0.21
δ-Cadinene	11.72	1520	0.24	10.45*	1702	[1.35]
10-epi-Cubebol?	11.79	1526	0.03	13.81	1998	0.04
(*E*)-γ-Bisabolene	11.84	1530	0.15	10.52	1707	0.10
Neryl butyrate	11.88	1533	0.04			
α-Elemol	12.04	1545	0.04	14.09	2025	0.03
Germacrene B	12.09	1549	0.03	11.27	1770	0.03
Geranyl butyrate	12.25	1562	0.11	12.20	1851	0.13
Caryophyllene oxide	12.40	1574	0.46	12.81	1906	0.41
Humulene epoxide II	12.73	1599	0.04	13.40	1960	0.04
Selin-6-en-4α-ol isomer	12.84	1608	0.02	14.86	2099	0.01
1-epi-Cubenol	13.00	1621	0.02	13.87	2004	0.02
Cubenol	13.17	1636	0.03	13.72	1989	0.04
β-Eudesmol	13.23	1641	0.01	15.51	2164	0.02
α-Eudesmol	13.29	1645	0.01	15.38	2151	0.01
(2*Z*,6*Z*)-Farnesol	13.53	1665	0.02	16.12	2226	0.01
Farnesal isomer	14.01	1705	0.03			
(2*E*,6*E*)-Farnesal	14.40	1739	0.01	15.88	2201	0.01
Neophytadiene	15.53	1839	0.01	12.68	1894	0.01
meta-Camphorene	16.73	1950	0.01	15.43	2156	0.06
Unknown [m/z 93, 69 (95), 135 (76), 107 (53), 41 (53), 109 (50)... 235 (10)...]	17.97	2072	0.04			
Dicitral	18.29	2104	0.03	16.88*	2305	[0.23]
Phytol isomer	18.59	2134	0.05			
Unknown [m/z 94, 43 (85), 93 (81), 69 (76), 137 (76), 95 (60), 134 (51)...]	18.92	2169	0.02			
Unknown [m/z 94, 43 (56), 123 (55), 69 (53), 95 (42), 79 (39)...]	19.43	2223	tr			

Unknown [m/z 93, 69 (79), 43 (70), 137 (53), 41 (41), 119 (37)... 289 (33)...]	19.58	2240	0.01	
Unknown [m/z 123, 94 (100), 43 (86), 69 (75), 95 (47), 41 (47), 93 (45)...]	19.76	2258	0.01	
**Total identified**	**98.06%**			**96.83%**
**Total reported**	**98.37%**			**97.30%**

*: Two or more compounds are coeluting on this column
[xx]: Duplicate percentage due to coelutions, not taken into account in the consolidated total
†: Peaks apexes were resolved, but peaks overlapped and were summed for analysis
tr: The compound has been detected below 0.005% of total signal.

Note: no correction factor was applied
R.T.: Retention time (minutes)
R.I.: Retention index
