# Supplementary material for: Antifungal Activity of Select Essential Oils against Candida auris and Their Interactions with Antifungal Drugs
Source: Pathogens. 2022 Jul 22;11(8):821. doi: 10.3390/pathogens11080821 (PMC9331469; doi:10.3390/pathogens11080821)
Supplement: Supplementary file 1 [file pathogens-11-00821-s001.zip › S4/Lemongrass EO- EO2823.pdf]

mountain rose herbs®
.com

An Herbs, Health, and Harmony Company.
Since 1987

CERTIFICATE OF ANALYSIS

Mailing: PO Box 50220 / Eugene, Oregon 97405
Phone: 800-879-3337 / Fax 510-217-4012
E-mail: qc@mountainroseherbs.com
www.mountainroseherbs.com

Product Name: Lemongrass Essential Oil
Botanical Name: *Cymbopogon flexuosus*
Origin: Sri Lanka
Manufacture Date: February 2020
Part Used: Grass
Lot Number: EO2823
Extraction: Distillation
Grade: Certified Organic
Additives: None
Notes: None

Test	Results
Appearance	Pale yellow
Odor	Heavy, lemony, green
Specific Gravity @20°C	0.8946*
Refractive Index @20°C	1.4839*
Optical Rotation @20°C	-2°03*

*By Vendor Report #OLG3190004

Rachelle Theobald   4/23/20

Christine Rice / Rachelle Theobald
Quality Control Department

Date

This information is presented in good faith and was compiled through testing methods in our laboratory, and with the assistance of our suppliers, harvesters, and processors information. We make no warranty, either expressed or implied in the complete accuracy of the information listed herein. The data in this analysis is offered solely for your verification and consideration. It is the responsibility of the buyer to provide themselves with up to date analyses for any botanicals purchased through Mountain Rose Herbs.

RK
