# Supplementary material for: Antifungal Activity of Select Essential Oils against Candida auris and Their Interactions with Antifungal Drugs
Source: Pathogens. 2022 Jul 22;11(8):821. doi: 10.3390/pathogens11080821 (PMC9331469; doi:10.3390/pathogens11080821)
Supplement: Supplementary file 1 [file pathogens-11-00821-s001.zip › S4/Lime Peel EO- EO2827.pdf]

mountain rose herbs ®
.com

An Herbs, Health, and Harmony Company.
Since 1987

# CERTIFICATE OF ANALYSIS

Mailing: PO Box 50220 / Eugene, Oregon 97405
Phone: 800-879-3337 / Fax 510-217-4012
E-mail: qc@mountainroseherbs.com
www.mountainroseherbs.com

**Product Name:** Lime Peel Essential Oil
**Botanical Name:** *Citrus aurantifolia*
**Origin:** Mexico
**Manufacture Date:** March 2020
**Part Used:** Fruit Peel
**Lot Number:** EO2827
**Extraction:** Cold pressed
**Grade:** Certified Organic
**Additives:** None
**Notes:** None

| Test | Results |
|---|---|
| Appearance | Greenish brown |
| Odor | Tart, sweet citrus |
| Density @20°C | 0.858* |
| Refractive Index @20°C | 39.9* |
| Optical Rotation @20°C | 1.475 |

*By Vendor Report #200309

Rachelle Theobald (signature) 4/20/20

Christine Rice / Rachelle Theobald
Date
Quality Control Department

This information is presented in good faith and was compiled through testing methods in our laboratory, and with the assistance of our suppliers, harvesters, and processors information. We make no warranty, either expressed or implied in the complete accuracy of the information listed herein. The data in this analysis is offered solely for your verification and consideration. It is the responsibility of the buyer to provide themselves with up to date analyses for any botanicals purchased through Mountain Rose Herbs.

RK
