# Supplementary material for: Antifungal Activity of Select Essential Oils against Candida auris and Their Interactions with Antifungal Drugs
Source: Pathogens. 2022 Jul 22;11(8):821. doi: 10.3390/pathogens11080821 (PMC9331469; doi:10.3390/pathogens11080821)
Supplement: Supplementary file 1 [file pathogens-11-00821-s001.zip › S4/MRH_EO_QC.pdf]

mountain rose herbs®
.com

A Herbs, Health and Harmony Company.
Since 1987

CERTIFICATE OF ANALYSIS

Mailing: PO Box 50220 / Eugene, Oregon 97405
Phone: 800-879-3337 / Fax 510-217-4012
E-mail: lab@mountainroseherbs.com
www.mountainroseherbs.com

Product Name: Basil Essential Oil
Botanical Name: *Ocimum basilicum*
Chemotype: *Methyl chavicol*
Origin: India
Manufacture Date: November 2017
Part Used: Flowering plant
Lot Number: EO2487
Extraction: Distillation
Grade: Certified Organic
Additives: None

Test	Results
Appearance	Translucent, almost colorless
Odor	Fresh, warm, spicy, herbaceous
Refractive Index @20°C	1.5055*
Specific Gravity @20°C	0.942*
Optical Rotation @20°C	-6.50°*

*By Vendor Report# 170760

3/14/18

Christine Rice / Steven Yeager **Date**
Quality Control Department

This information is presented in good faith and was compiled through testing methods in our laboratory, and with the assistance of our suppliers, harvesters, and processors information. We make no warranty, either expressed or implied in the complete accuracy of the information listed herein. The data in this analysis is offered solely for your verification and consideration. It is the responsibility of the buyer to provide themselves with up to date analyses for any botanicals purchased through Mountain Rose Herbs.

mountain rose herbs®
.com

An Herbs, Health, and Harmony Company.
Since 1987

CERTIFICATE OF ANALYSIS

Mailing: PO Box 50220 / Eugene, Oregon 97405
Phone: 800-879-3337 / Fax 510-217-4012
E-mail: qc@mountainroseherbs.com
www.mountainroseherbs.com

Product Name: Bergamot Essential Oil
Botanical Name: *Citrus bergamia*
Origin: Italy
Manufacture Date: March 2020
Part Used: Fruit Peel (Bergaptene Free)
Lot Number: EO2842
Extraction: Cold pressed
Grade: Certified Organic
Additives: None
Notes: None

Test	Results
Appearance	Translucent, almost colorless
Odor	Fresh, spicy, floral, citrus
Relative density (d20/20)	0.860*
Refractive Index @ 20°C	1.466*
Angular Rotation	+42.0°*

*By Vendor Report #99BGS1208420B

Rachelle Theobald (signature) 6/11/20

Christine Rice / Rachelle Theobald **Date**
Quality Control Department

This information is presented in good faith and was compiled through testing methods in our laboratory, and with the assistance of our suppliers, harvesters, and processors information. We make no warranty, either expressed or implied in the complete accuracy of the information listed herein. The data in this analysis is offered solely for your verification and consideration. It is the responsibility of the buyer to provide themselves with up to date analyses for any botanicals purchased through Mountain Rose Herbs.

rk

mountain rose herbs.com

CERTIFICATE OF ANALYSIS

Mailing: PO Box 50220 / Eugene, Oregon 97405
Phone: 800-879-3337 / Fax 510-217-4012
E-mail: qc@mountainroseherbs.com
www.mountainroseherbs.com

Product Name: Bitter Orange Essential Oil
Botanical Name: *Citrus aurantium*
Origin: Egypt
Production Date: December 2019
Part Used: Fruit Peel
Lot Number: EO2911
Extraction: Cold Pressed
Grade: Certified Organic
Additives: N/A

Test	Specifications	Results	Method
Identity	Passed	Passed	Organoleptic

Rachelle Theobald 11/20/20

Steven Yeager / Rachelle Theobald / Geri Green **Date**
Quality Control Department

This information is presented in good faith and was compiled through testing methods in our laboratory, contracted laboratories, and with the assistance of our suppliers, harvesters, and processors information. We make no warranty, either expressed or implied in the complete accuracy of the information listed herein. The data in this analysis is offered solely for your verification and consideration. It is the responsibility of the buyer to provide themselves with up to date analyses for any botanicals purchased through Mountain Rose Herbs.

smt

mountain rose herbs.com

CERTIFICATE OF ANALYSIS

Mailing: PO Box 50220 / Eugene, Oregon 97405
Phone: 800-879-3337 / Fax 510-217-4012
E-mail: qc@mountainroseherbs.com
www.mountainroseherbs.com

Product Name: Cinnamon Bark Essential Oil
Botanical Name: *Cinnamomum zeylanicum*
Origin: Sri Lanka
Production Date: August 2020
Part Used: Bark
Lot Number: FTEO2908
Extraction: Distillation
Grade: Certified Organic and Fair Trade Certified
Additives: None

Test	Specifications	Results	Method
Identity	Passed	Passed	Organoleptic

Rachelle Theobald (signature) 11/11/20

Steven Yeager / Rachelle Theobald / Geri Green
Quality Control Department
Date

This information is presented in good faith and was compiled through testing methods in our laboratory, contracted laboratories, and with the assistance of our suppliers, harvesters, and processors information. We make no warranty, either expressed or implied in the complete accuracy of the information listed herein. The data in this analysis is offered solely for your verification and consideration. It is the responsibility of the buyer to provide themselves with up to date analyses for any botanicals purchased through Mountain Rose Herbs.

smt

mountain rose herbs.com

CERTIFICATE OF ANALYSIS

Mailing: PO Box 50220 / Eugene, Oregon 97405
Phone: 800-879-3337 / Fax 510-217-4012
E-mail: qc@mountainroseherbs.com
www.mountainroseherbs.com

Product Name: Cinnamon Leaf Essential Oil
Botanical Name: *Cinnamomum zeylanicum*
Origin: Sri Lanka
Production Date: March 2020
Part Used: Leaf
Lot Number: EO2910
Extraction: Distillation
Grade: Certified Organic
Additives: N/A

Test	Specifications	Results	Method
Identity	Passed	Passed	Organoleptic

Rachelle Theobald 11/20/20
Steven Yeager / Rachelle Theobald / Geri Green **Date**
Quality Control Department

This information is presented in good faith and was compiled through testing methods in our laboratory, contracted laboratories, and with the assistance of our suppliers, harvesters, and processors information. We make no warranty, either expressed or implied in the complete accuracy of the information listed herein. The data in this analysis is offered solely for your verification and consideration. It is the responsibility of the buyer to provide themselves with up to date analyses for any botanicals purchased through Mountain Rose Herbs.

smt

Clove Bud Essential Oil- EO2934

alpha-Cubebene	0.09
Furfural	0.05
α- Copaene	0.20
β- Caryophyllene	7.96
α- Humulene	0.61
δ- Cadinene	0.22
Methyl Salicylate	0.13
trans-Calamenene	0.08
Caryophyllene Oxide	0.15
Methyl Eugenol ether	0.03
Caryophyllenyl Alcohol	0.06
Eugenol	82.17
Eugenol Acetate	7.37
Chavicol	0.19

mountain rose herbs®
.com

An Herbs, Health, and Harmony Company.
Since 1987

CERTIFICATE OF ANALYSIS

Mailing: PO Box 50220 / Eugene, Oregon 97405
Phone: 800-879-3337 / Fax 510-217-4012
E-mail: qc@mountainroseherbs.com
www.mountainroseherbs.com

Product Name: Coriander Seed Essential Oil
Botanical Name: *Coriandrum sativum*
Origin: Hungary
Manufacture Date: November 2019
Part Used: Seed
Lot Number: EO2851
Extraction: Distillation
Grade: Certified Organic
Additives: None
Notes: None

Test	Results
Appearance	Light yellow
Odor	Sweet, spicy, woody, slight fruity top note

Rachelle Theobald 6/12/20
Christine Rice / Rachelle Theobald — Date
Quality Control Department

This information is presented in good faith and was compiled through testing methods in our laboratory, and with the assistance of our suppliers, harvesters, and processors information. We make no warranty, either expressed or implied in the complete accuracy of the information listed herein. The data in this analysis is offered solely for your verification and consideration. It is the responsibility of the buyer to provide themselves with up to date analyses for any botanicals purchased through Mountain Rose Herbs.

rk

mountain rose herbs ®
.com

An Herbs, Health, and Harmony Company.
Since 1987

CERTIFICATE OF ANALYSIS

Mailing: PO Box 50220 / Eugene, Oregon 97405
Phone: 800-879-3337 / Fax 510-217-4012
E-mail: qc@mountainroseherbs.com
www.mountainroseherbs.com

Product Name: Eucalyptus Essential Oil
Botanical Name: *Eucalyptus globulus*
Origin: China
Manufacture Date: April 2020
Part Used: Leaf and Twig
Lot Number: EO2835
Extraction: Distillation
Grade: Certified Organic
Additives: None
Notes: None

Test	Results
Appearance	Translucent, almost colorless
Odor	Fresh, woody, camphoraceous
Specific Gravity @25°C	0.9086*
Refractive Index @20°C	1.460*

*By Vendor Report #2004000582

Rachelle Theobald (signature) 6/5/20

Christine Rice / Rachelle Theobald **Date**
Quality Control Department

This information is presented in good faith and was compiled through testing methods in our laboratory, and with the assistance of our suppliers, harvesters, and processors information. We make no warranty, either expressed or implied in the complete accuracy of the information listed herein. The data in this analysis is offered solely for your verification and consideration. It is the responsibility of the buyer to provide themselves with up to date analyses for any botanicals purchased through Mountain Rose Herbs.

rk

Date : May 29, 2020

Certificate of Analysis – GC Profiling

Sample identification

Internal code : 20E19-MRH01
Customer identification : Eucalyptus - China - EO2835
Type : Essential oil
Source : *Eucalyptus globulus*
Customer : Mountain Rose Herbs

Analysis

Method: PC-MAT-007 - Analysis of the composition of an essential oil or other volatile liquide by FAST GC-FID (in French); identifications validated by GC-MS.
Analyst : Fanny Charlier, B. Sc.
Analysis date : May 20, 2020

Checked and approved by :

Alexis St-Gelais, M. Sc., chimiste 2013-174

Notes: This report may not be published, including online, without the written consent from Laboratoire PhytoChemia. This report is digitally signed, it is only considered valid if the digital signature is intact. The results only describe the samples that were submitted to the assays.

Laboratoire PhytoChemia

628 Boulevard du Saguenay Ouest, Saguenay (Qc) G7J 1H4 | www.phytochemia.com

PHYSICOCHEMICAL DATA

Physical aspect: Clear liquid
Refractive index: 1.4605 ± 0.0003 (20 °C; method PC-MAT-016)

CONCLUSION

No adulterant, contaminant or diluent has been detected using this method.

628 Boulevard du Saguenay, Saguenay (Qc) G7J 1H4 | www.phytochemia.com

ANALYSIS SUMMARY – CONSOLIDATED CONTENTS

New readers of similar reports are encouraged to read table footnotes at least once.

Identification	%	Class
Isovaleral	tr	Aliphatic aldehyde
Isoamyl alcohol	0.01	Aliphatic alcohol
Hashishene	0.01	Monoterpene
α-Thujene	0.03	Monoterpene
α-Pinene	4.80	Monoterpene
Camphene	0.04	Monoterpene
α-Fenchene	0.02	Monoterpene
Thuja-2,4(10)-diene	0.01	Monoterpene
β-Pinene	0.36	Monoterpene
trans-Dehydroxylinalool oxide	0.02	Monoterpenic ether
Myrcene	0.60	Monoterpene
α-Phellandrene	1.22	Monoterpene
Δ3-Carene	0.07	Monoterpene
α-Terpinene	0.30	Monoterpene
para-Cymene	0.75	Monoterpene
Limonene	2.94	Monoterpene
1,8-Cineole	82.88	Monoterpenic ether
β-Phellandrene	3.95	Monoterpene
(*Z*)-β-Ocimene	0.09	Monoterpene
(*E*)-β-Ocimene	0.06	Monoterpene
γ-Terpinene	1.62	Monoterpene
Unknown	0.01	Oxygenated monoterpene
meta-Mentha-4,6-dien-8-ol	0.01	Monoterpenic alcohol
Terpinen-4-ol	tr	Monoterpenic alcohol
α-Terpineol	0.01	Monoterpenic alcohol
Unknown	0.02	Unknown
α-Gurjunene	0.01	Sesquiterpene
(*trans*?)-6-Hydroxypiperitone	0.01	Monoterpenic alcohol
β-Caryophyllene	0.01	Sesquiterpene
γ-Cadinene	0.03	Sesquiterpene
δ-Cadinene	0.02	Sesquiterpene
trans-Cadina-1,4-diene	0.03	Sesquiterpene
Aromadendrene	0.01	Sesquiterpene
**Consolidated total**	**99.94%**	

tr: The compound has been detected below 0.005% of total signal.

Note: no correction factor was applied

About "consolidated" data: The table above presents the breakdown of the sample volatile constituents after applying an algorithm to collapse data acquired from the multi-columns system of PhytoChemia into a single set of consolidated contents. In case of discrepancies between columns, the algorithm is set to prioritize data from the most standard DB-5 column, and smallest values so as to avoid overestimating individual content. This process is semi-automatic. Advanced users are invited to consult the "Full analysis data" table after the chromatograms in this report to access the full untreated data and perform their own calculations if needed.

Unknowns: Unknown compounds' mass spectral data is presented in the "Full analysis data" table. The occurrence of unknown compounds is to be expected in many samples, and does not denote particular problems unless noted otherwise in the conclusion.

This page was intentionally left blank. The following pages present the complete data of the analysis.

628 Boulevard du Saguenay, Saguenay (Qc) G7J 1H4 | www.phytochemia.com

DB-5

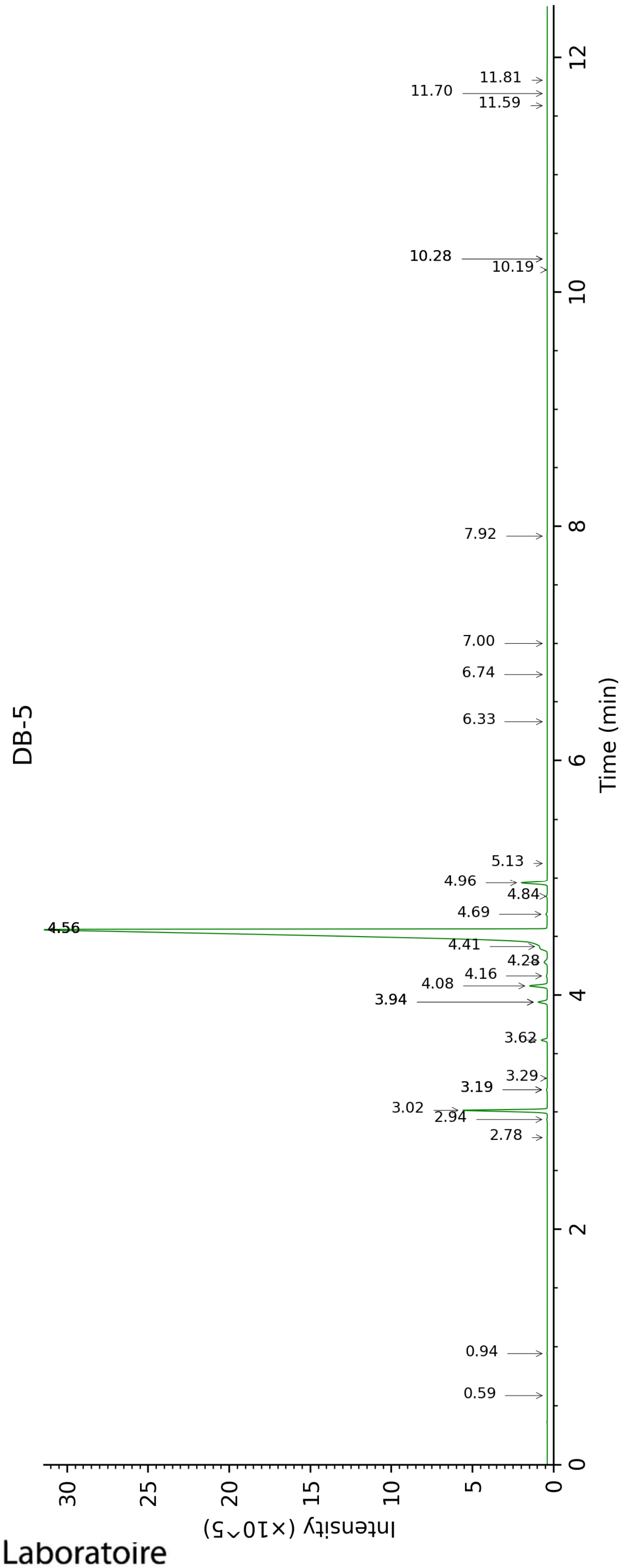
11.81
11.70
11.59
10.28
10.19
7.92
7.00
6.74
6.33
5.13
4.96
4.84
4.69
4.56
4.41
4.28
4.16
4.08
3.94
3.62
3.29
3.19
3.02
2.94
2.78
0.94
0.59
0
2
4
6
8
10
12
Time (min)
0
5
10
15
20
25
30
Intensity (×10^5)

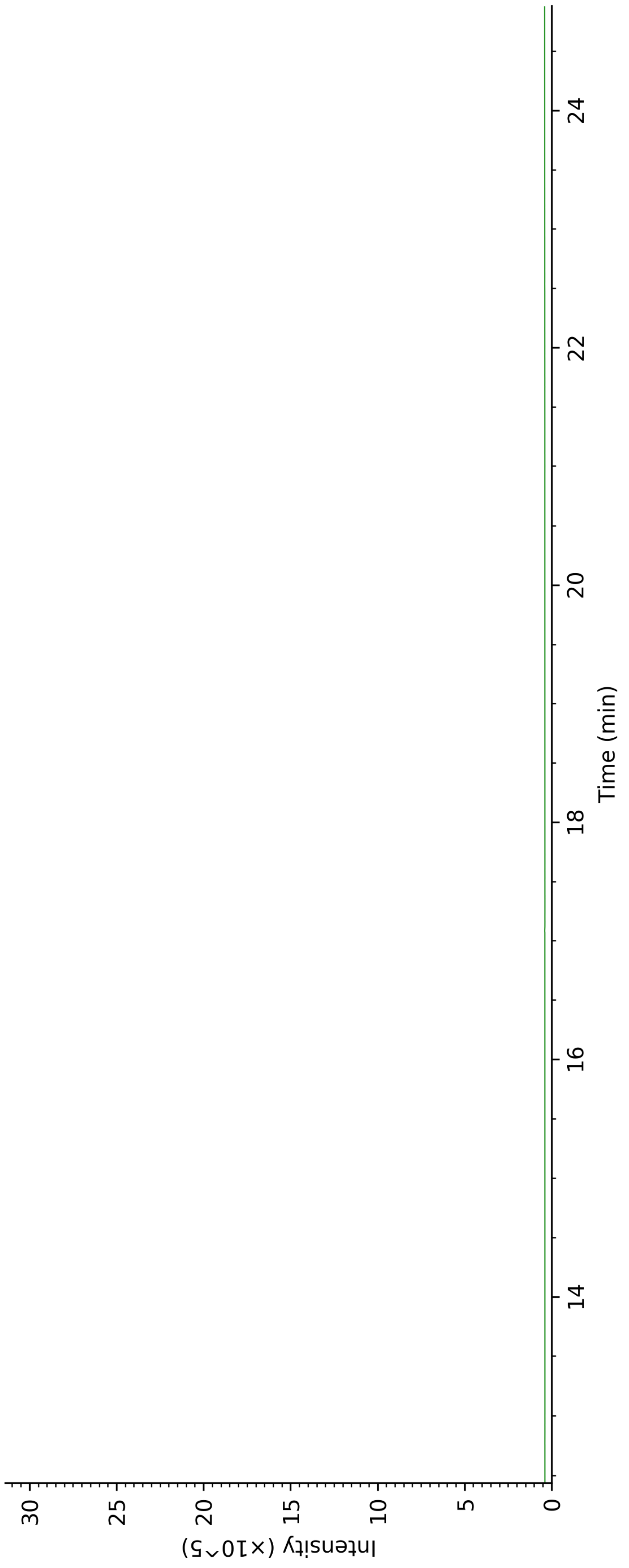
14
16
18
20
22
24
Time (min)
0
5
10
15
20
25
30
Intensity (×10^5)

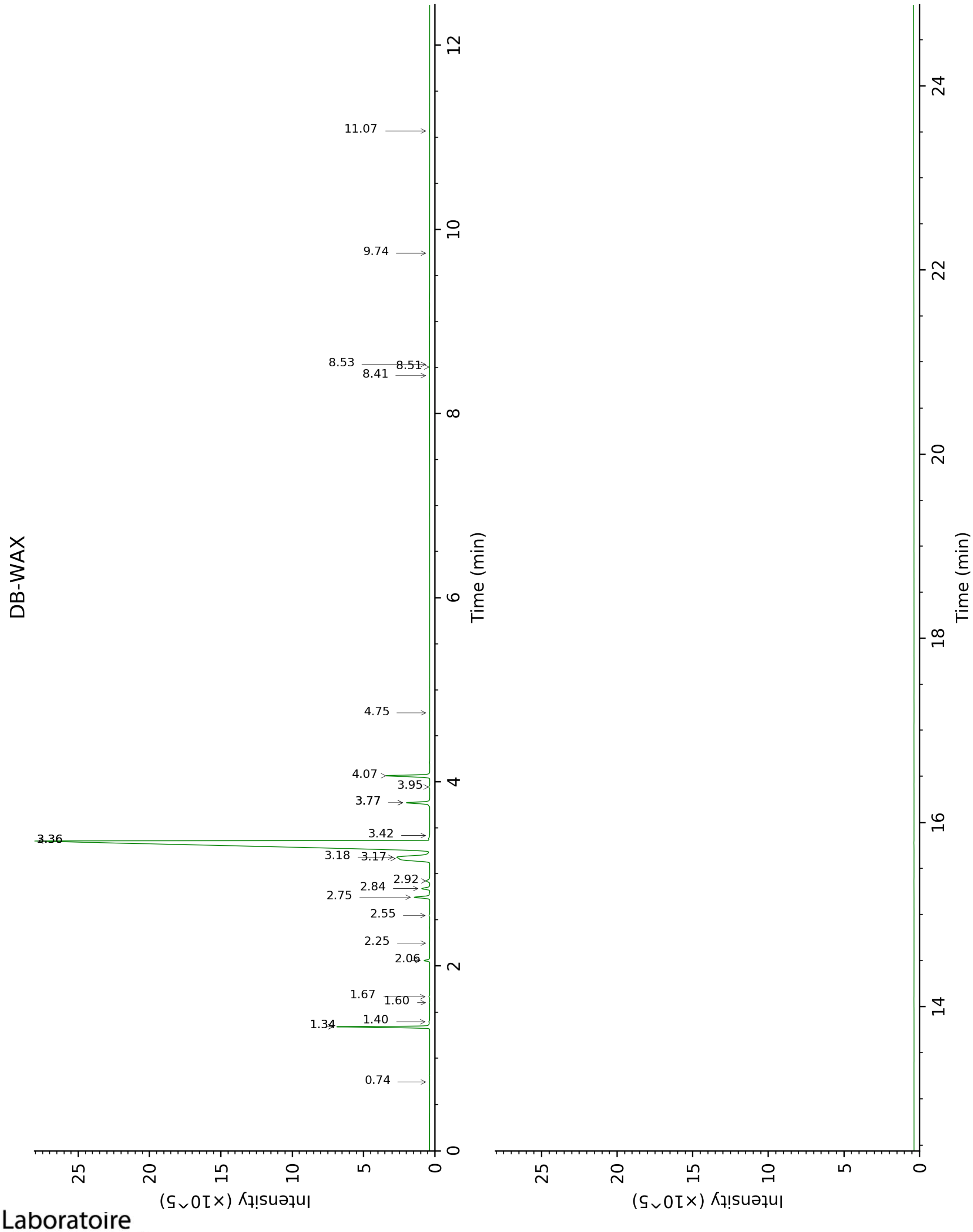
DB-WAX
Time (min)
Intensity (×10^5)
0.74
1.34
1.40
1.60
1.67
2.06
2.25
2.55
2.75
2.84
2.92
3.17
3.18
3.36
3.42
3.77
3.95
4.07
4.75
8.41
8.51
8.53
9.74
11.07

FULL ANALYSIS DATA

Identification	Column DB-5			Column DB-WAX		
	R.T	R.I	%	R.T	R.I	%
Isovaleral	0.59	640	tr	0.74	885	tr
Isoamyl alcohol	0.94	737	0.01	3.42	1177	0.02
Hashishene	2.78	915	0.01	1.34*	990	4.74
α-Thujene	2.94	925	0.03	1.40	998	0.04
α-Pinene	3.02	930	4.80	1.34*	990	[4.74]
Camphene	3.20*	942	0.05	1.67	1025	0.04
α-Fenchene	3.20*	942	[0.05]	1.60	1018	0.02
Thuja-2,4(10)-diene	3.29	948	0.01	2.25	1082	0.02
β-Pinene	3.62	970	0.36	2.06	1064	0.36
trans-Dehydroxylinalool oxide	3.94*	991	0.62	3.36*	1172	80.23
Myrcene	3.94*	991	[0.62]	2.84	1131	0.60
α-Phellandrene	4.08	1001	1.22	2.75	1124	1.20
Δ3-Carene	4.16	1006	0.07	2.55	1108	0.04
α-Terpinene	4.28	1013	0.30	2.92	1138	0.31
para-Cymene	4.41	1022	0.75	4.07	1226	3.16
Limonene	4.56*	1031	89.77	3.17	1157	2.94
1,8-Cineole	4.56*	1031	[89.77]	3.36*	1172	[80.23]
β-Phellandrene	4.56*	1031	[89.77]	3.18	1158	3.95
(*Z*)-β-Ocimene	4.69	1039	0.09	3.78*	1204	1.77
(*E*)-β-Ocimene	4.84	1049	0.06	3.95	1217	0.06
γ-Terpinene	4.96	1056	1.62	3.78*	1204	[1.77]
Unknown [m/z 79, 93 (60), 43 (40), 94 (35), 137 (33), 77 (26), 91 (20), 152 (18)]	5.13	1067	0.01	4.75	1276	0.01
meta-Mentha-4,6-dien-8-ol	6.33	1144	0.01			
Terpinen-4-ol	6.74	1170	tr	8.53	1552	0.01
α-Terpineol	7.00	1188	0.01	9.74	1648	0.01
Unknown [m/z 43, 97 (69), 107 (46), 41 (28), 55 (21), 109 (20)...]	7.92	1250	0.02	11.07	1758	0.02
α-Gurjunene	10.19	1404	0.01			
(*trans*?)-6-Hydroxypiperitone	10.28*	1411	0.02			
β-Caryophyllene	10.28*	1411	[0.02]	8.41	1543	0.01
γ-Cadinene	11.59	1509	0.03			
δ-Cadinene	11.70	1517	0.02			
trans-Cadina-1,4-diene	11.81	1526	0.03			
Aromadendrene				8.51	1550	0.01
**Total identified**		**99.90%**			**99.54%**	
**Total reported**		**99.92%**			**99.56%**	

*: Two or more compounds are coeluting on this column
[xx]: Duplicate percentage due to coelutions, not taken into account in the consolidated total
†: Peaks apexes were resolved, but peaks overlapped and were summed for analysis
tr: The compound has been detected below 0.005% of total signal.

Note: no correction factor was applied
R.T.: Retention time (minutes)
R.I.: Retention index

628 Boulevard du Saguenay, Saguenay (Qc) G7J 1H4 | www.phytochemia.com

mountain rose herbs.com

CERTIFICATE OF ANALYSIS

Mailing: PO Box 50220 / Eugene, Oregon 97405
Phone: 800-879-3337 / Fax 510-217-4012
E-mail: qc@mountainroseherbs.com
www.mountainroseherbs.com

Product Name: Frankincense Essential Oil
Botanical Name: *Boswellia carteri*
Origin: France
Production Date: November 2019
Part Used: Resin
Lot Number: EO2886
Extraction: Distillation
Grade: Certified Organic
Additives: None

Test	Specifications	Results	Method
Identity	Passed	Passed	Organoleptic

Rachelle Theobald — 9/10/20

Steven Yeager / Rachelle Theobald / Geri Green — **Date**
Quality Control Department

This information is presented in good faith and was compiled through testing methods in our laboratory, contracted laboratories, and with the assistance of our suppliers, harvesters, and processors information. We make no warranty, either expressed or implied in the complete accuracy of the information listed herein. The data in this analysis is offered solely for your verification and consideration. It is the responsibility of the buyer to provide themselves with up to date analyses for any botanicals purchased through Mountain Rose Herbs.

rk

mountain rose herbs .com

CERTIFICATE OF ANALYSIS

Mailing: PO Box 50220 / Eugene, Oregon 97405
Phone: 800-879-3337 / Fax 510-217-4012
E-mail: qc@mountainroseherbs.com
www.mountainroseherbs.com

Product Name: Geranium Essential Oil
Botanical Name: *Pelargonium graveolens*
Origin: Egypt
Production Date: July 2020
Part Used: Aerial Portion
Lot Number: EO2950
Extraction: Distillation
Grade: Certified Organic
Additives: None

Test	Specifications	Results	Method
Identity	Passed	Passed	Organoleptic

Rachelle Theobald 2/2/21

Steven Yeager / Rachelle Theobald / Geri Green **Date**
Quality Control Department

This information is presented in good faith and was compiled through testing methods in our laboratory, contracted laboratories, and with the assistance of our suppliers, harvesters, and processors information. We make no warranty, either expressed or implied in the complete accuracy of the information listed herein. The data in this analysis is offered solely for your verification and consideration. It is the responsibility of the buyer to provide themselves with up to date analyses for any botanicals purchased through Mountain Rose Herbs.

smt

Geranium EO- EO2950

			normes/norms	résultat/results
ASPECT / APPEARANCE<br>*CQ-ME-01-16*	LIQUIDE LIMPIDE / LIMPID LIQUID		LIQUIDE LIMPIDE	Conforme / Conform
COULEUR / COLOR<br>*CQ-ME-01-16*	JAUNE PALE / PALE YELLOW		JAUNE PALE A JAUNE FONCE A VERDATRE PARFOIS	Conforme / Conform
ODEUR / ODOR<br>*CQ-ME-01-16*	CONFORME / CONFORM		ROSEE, HERBACEE	Conforme / Conform
DENSITE (20°) / SPECIFIC GRAVITY 20°C<br>*CQ-ME-01-23*	0.897	G/CM3	0,885 - 0,905	Conforme / Conform
INDICE REFRACTION (20°) / REFRACTIVE INDEX 20°C<br>*CQ-ME-01-08*	1.466		1,461 - 1,475	Conforme / Conform
POUVOIR ROTATOIRE / SPECIFIC OPTICAL ROTATION<br>*CQ-ME-01-05*	-11.4	DEG	-14 - -8	Conforme / Conform
SOLUBILITE ALCOOL 70° / ALCOHOL SOLUBILITY 70°<br>*CQ-ME-01-27*	2	VOL	<= 3	Conforme / Conform
10 EPI GAMMA EUDESMOL<br>*CQ-ME-01-64*	4.1	%	3 - 6	Conforme / Conform
ALPHA TERPINEOL<br>*CQ-ME-01-64*	0.3	%	0,3 - 0,6	Conforme / Conform
CIS ROSE OXYDE<br>*CQ-ME-01-64*	1.1	%	0,7 - 1,5	Conforme / Conform
CITRONNELLOL<br>*CQ-ME-01-64*	32.7	%	25 - 36	Conforme / Conform
FORMIATE DE CITRONELLYLE<br>*CQ-ME-01-64*	7.1	%	5 - 8	Conforme / Conform
LINALOL<br>*CQ-ME-01-64*	4.8	%	4 - 8,5	Conforme / Conform
PHENYL ETHYL TIGLATE<br>*CQ-ME-01-64*	0.7	%	0,5 - 1,2	Conforme / Conform
TRANS ROSE OXYDE<br>*CQ-ME-01-64*	0.4	%	0,3 - 0,6	Conforme / Conform

GERANIOL<br>*CQ-ME-01-64*	11.6	%	10 - 18	Conforme / Conform
GERANYLE BUTYRATE<br>*CQ-ME-01-64*	1	%	0,7 - 2	Conforme / Conform
GERANYLE FORMIATE<br>*CQ-ME-01-64*	2.7	%	2 - 7	Conforme / Conform
GERANYLE TIGLATE<br>*CQ-ME-01-64*	1	%	0,9 - 2	Conforme / Conform
GUAIA-6-9-DIENE<br>*CQ-ME-01-64*	0.3	%	<= 0,5	Conforme / Conform
ISOMENTHONE<br>*CQ-ME-01-64*	6.3	%	4 - 8	Conforme / Conform
ANALYSE CPG / GC ANALYSIS<br>*CQ-ME-01-64*	CONFORME / CONFORM			Conforme / Conform

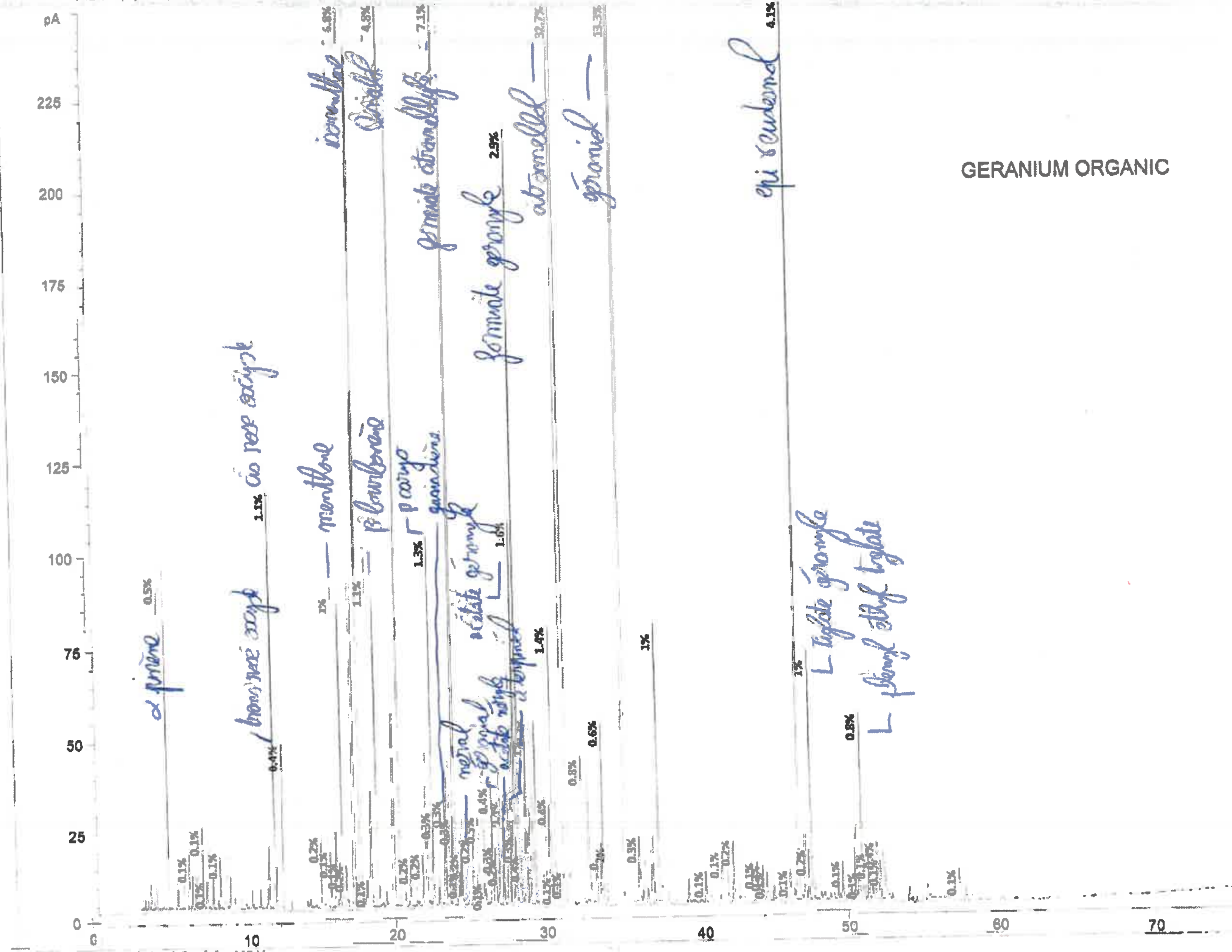
GERANIUM ORGANIC
pA
α pinène
trans rose oxyde
cis rose oxyde
menthone
isomenthone
β bourbonène
β caryo
guaiadiène
formiate citronellyle
néral
géranial
acétate néryle
α terpinéol
acétate géranyle
formiate géranyle
citronellol
géraniol
épi γ eudesmol
tiglate géranyle
phényl éthyl tiglate

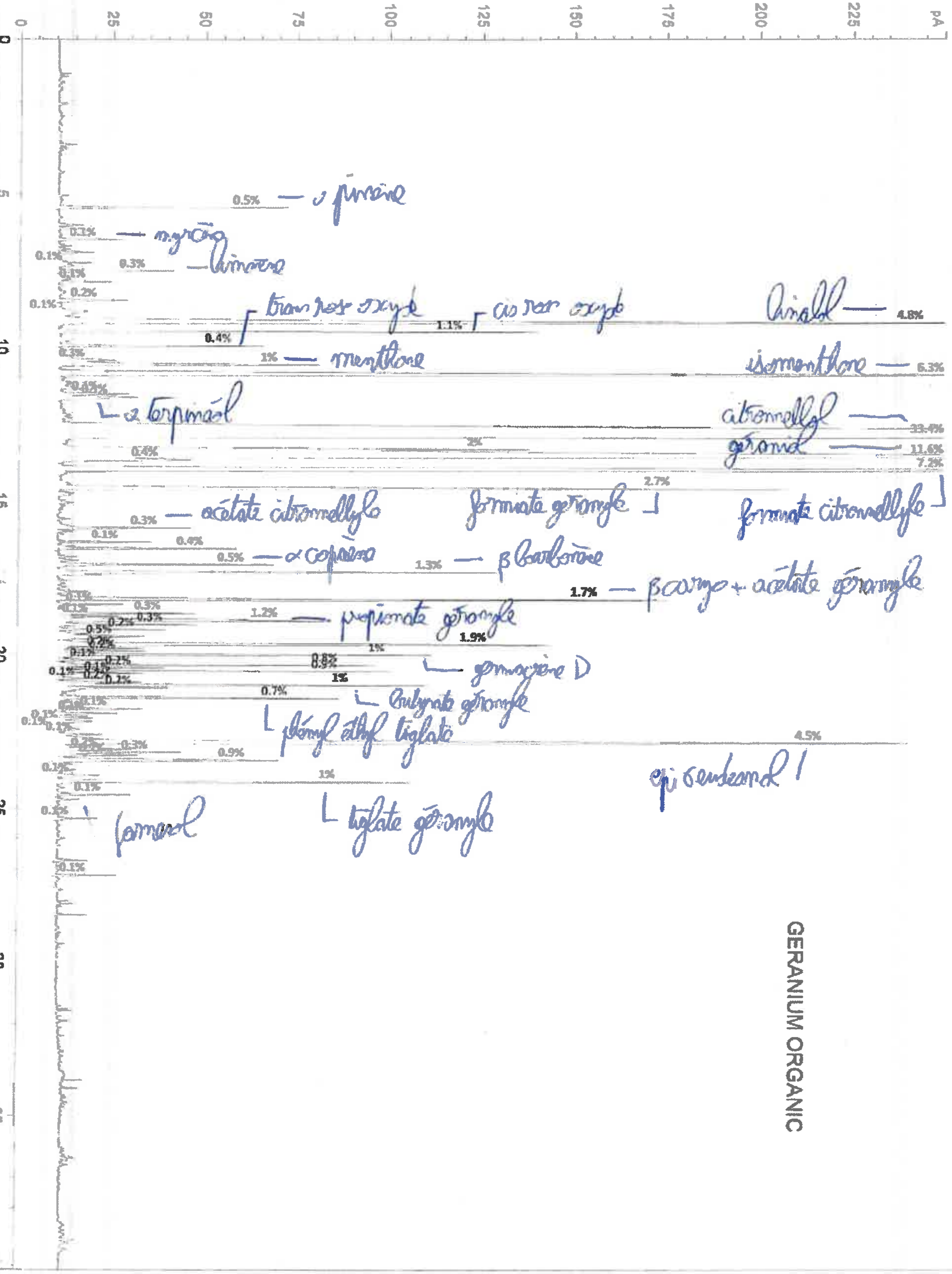

GERANIUM ORGANIC
α pinène
myrcène
limonène
trans rose oxyde
cis rose oxyde
linalol
menthone
isomenthone
α terpinéol
citronellol
géraniol
acétate citronellyle
formiate géranyle
formiate citronellyle
α copaène
β bourbonène
β caryo + acétate géranyle
propionate géranyle
germacrène D
butyrate géranyle
phényl éthyl tiglate
épi γ eudesmol
farnesol
tiglate géranyle

mountain rose herbs .com

CERTIFICATE OF ANALYSIS

Mailing: PO Box 50220 / Eugene, Oregon 97405
Phone: 800-879-3337 / Fax 510-217-4012
E-mail: qc@mountainroseherbs.com
www.mountainroseherbs.com

Product Name: Grapefruit Essential Oil
Botanical Name: *Citrus paradisi*
Origin: Paraguay
Production Date: October 2020
Part Used: Peel
Lot Number: EO2914
Extraction: Cold Pressed
Grade: Certified Organic
Additives: N/A

Test	Specifications	Results	Method
Identity	Passed	Passed	Organoleptic

Rachelle Theobald   11/30/20

Steven Yeager / Rachelle Theobald / Geri Green   **Date**
Quality Control Department

This information is presented in good faith and was compiled through testing methods in our laboratory, contracted laboratories, and with the assistance of our suppliers, harvesters, and processors information. We make no warranty, either expressed or implied in the complete accuracy of the information listed herein. The data in this analysis is offered solely for your verification and consideration. It is the responsibility of the buyer to provide themselves with up to date analyses for any botanicals purchased through Mountain Rose Herbs.

smt

mountain rose herbs .com

CERTIFICATE OF ANALYSIS

Mailing: PO Box 50220 / Eugene, Oregon 97405
Phone: 800-879-3337 / Fax 510-217-4012
E-mail: qc@mountainroseherbs.com
www.mountainroseherbs.com

Product Name: Lavender Essential Oil
Botanical Name: *Lavandula angustifolia*
Origin: Bulgaria
Production Date: December 2017
Part Used: Flowering Top
Lot Number: EO2918
Extraction: Distillation
Grade: Certified Organic
Additives: N/A

Test	Specifications	Results	Method
Identity	Passed	Passed	Organoleptic

Rachelle Theobald 11/20/20

Steven Yeager / Rachelle Theobald / Geri Green **Date**
Quality Control Department

This information is presented in good faith and was compiled through testing methods in our laboratory, contracted laboratories, and with the assistance of our suppliers, harvesters, and processors information. We make no warranty, either expressed or implied in the complete accuracy of the information listed herein. The data in this analysis is offered solely for your verification and consideration. It is the responsibility of the buyer to provide themselves with up to date analyses for any botanicals purchased through Mountain Rose Herbs.

smt

mountain rose herbs.com

CERTIFICATE OF ANALYSIS

Mailing: PO Box 50220 / Eugene, Oregon 97405
Phone: 800-879-3337 / Fax 510-217-4012
E-mail: qc@mountainroseherbs.com
www.mountainroseherbs.com

Product Name: Lemon Essential Oil
Botanical Name: *Citrus x limon*
Origin: Italy
Production Date: 2019
Part Used: Fruit Peel
Lot Number: EO2879
Extraction: Cold Pressed
Grade: Certified Organic
Additives: None

Test	Specifications	Results	Method
Identity	Passed	Passed	Organoleptic
Identity	Passed	Passed*	Fast GC-FID / GC-MS

*By Report #20H26-MRG01

Rachelle Theobald — 9/10/20

Steven Yeager / Rachelle Theobald / Geri Green — **Date**
Quality Control Department

This information is presented in good faith and was compiled through testing methods in our laboratory, contracted laboratories, and with the assistance of our suppliers, harvesters, and processors information. We make no warranty, either expressed or implied in the complete accuracy of the information listed herein. The data in this analysis is offered solely for your verification and consideration. It is the responsibility of the buyer to provide themselves with up to date analyses for any botanicals purchased through Mountain Rose Herbs.

rk

Lemon Essential Oil
lot #Eo2879

α- Pinene	2.49
Camphene	0.06
β-Pinene	12.65
Sabinene	2.02
β-Myrcene	1.53
α- Phellandrene	0.03
α-Terpinene	0.18
Limonene	66.56
β-Phellandrene	0.36
cis-beta-Ocimene	0.04
γ- Terpinene	9.41
para-Cymene	0.25
Terpinolene	0.38
6-methyl-5-hepten-2-one	0.05
Nonanal	0.07
Citronellal	0.07
Decanal	0.02
Linalool	0.09
cis-α- Bergamotene	0.20
β-Caryophyllene	0.11
Neral	0.55
α-Terpineol	0.11
Neryl Acetate	0.47
β- Bisabolene	0.48
Geranial	1.26
Geranyl Acetate	0.37
Geraniol	0.02

mountain rose herbs®
.com

An Herbs, Health, and Harmony Company.
Since 1987

CERTIFICATE OF ANALYSIS

Mailing: PO Box 50220 / Eugene, Oregon 97405
Phone: 800-879-3337 / Fax 510-217-4012
E-mail: qc@mountainroseherbs.com
www.mountainroseherbs.com

Product Name: Lemongrass Essential Oil
Botanical Name: *Cymbopogon flexuosus*
Origin: Sri Lanka
Manufacture Date: February 2020
Part Used: Grass
Lot Number: EO2823
Extraction: Distillation
Grade: Certified Organic
Additives: None
Notes: None

Test	Results
Appearance	Pale yellow
Odor	Heavy, lemony, green
Specific Gravity @20°C	0.8946*
Refractive Index @20°C	1.4839*
Optical Rotation @20°C	-2°03*

*By Vendor Report #OLG3190004

Rachelle Theobald
Christine Rice / Rachelle Theobald
Quality Control Department

4/23/20
Date

This information is presented in good faith and was compiled through testing methods in our laboratory, and with the assistance of our suppliers, harvesters, and processors information. We make no warranty, either expressed or implied in the complete accuracy of the information listed herein. The data in this analysis is offered solely for your verification and consideration. It is the responsibility of the buyer to provide themselves with up to date analyses for any botanicals purchased through Mountain Rose Herbs.

RK

Date : April 22, 2020

Certificate of Analysis – GC Profiling

Sample identification

Internal code : 20D15-MRH01
Customer identification : Lemongrass - Sri Lanka - EO2823
Type : Essential oil
Source : *Cymbopogon flexuosus*
Customer : Mountain Rose Herbs

Analysis

Method: PC-MAT-007 - Analysis of the composition of an essential oil or other volatile liquide by FAST GC-FID (in French); identifications validated by GC-MS.
Analyst : Fanny Charlier, B. Sc.
Analysis date : April 21, 2020

Checked and approved by :

Alexis St-Gelais, M. Sc., chimiste 2013-174

Notes: This report may not be published, including online, without the written consent from Laboratoire PhytoChemia. This report is digitally signed, it is only considered valid if the digital signature is intact. The results only describe the samples that were submitted to the assays.

Physicochemical data

Physical aspect: Yellow liquid
Refractive index: 1.4853 ± 0.0003 (20 °C; method PC-MAT-016)

Conclusion

No adulterant, contaminant or diluent has been detected using this method.

628 Boulevard du Saguenay, Saguenay (Qc) G7J 1H4 | www.phytochemia.com

ANALYSIS SUMMARY – CONSOLIDATED CONTENTS

New readers of similar reports are encouraged to read table footnotes at least once.

Identification	%	Classe
Ethanol	tr	Aliphatic alcohol
Acetone	0.02	Aliphatic ketone
Isovaleral	tr	Aliphatic aldehyde
2-Methylbutyral	tr	Aliphatic aldehyde
2-Ethylfuran	tr	Furan
Isoamyl alcohol	tr	Aliphatic alcohol
(3*Z*)-Hexenol	0.01	Aliphatic alcohol
Tricyclene	0.12	Monoterpene
α-Pinene	0.16	Monoterpene
Camphene	0.94	Monoterpene
α-Fenchene	0.02	Monoterpene
Thuja-2,4(10)-diene	0.01	Monoterpene
β-Pinene	tr	Monoterpene
Sabinene	0.02	Monoterpene
6-Methyl-5-hepten-2-one	0.88	Aliphatic ketone
Myrcene	0.06	Monoterpene
6-Methyl-5-hepten-2-ol	0.06	Aliphatic alcohol
Octanal	0.10	Aliphatic aldehyde
α-Terpinene	0.01	Monoterpene
para-Cymene	0.01	Monoterpene
Limonene	0.24	Monoterpene
1,8-Cineole	0.03	Monoterpenic ether
Benzeneacetaldehyde	0.01	Simple phenolic
(*Z*)-β-Ocimene	0.27	Monoterpene
(*E*)-β-Ocimene	0.16	Monoterpene
2,6-Dimethyl-5-heptenal (melonal)	0.02	Aliphatic aldehyde
γ-Terpinene	0.01	Monoterpene
cis-Linalool oxide (fur.)	0.01	Monoterpenic alcohol
4-Nonanone	1.10	Aliphatic ketone
Camphenilone	0.02	Normonoterpenic ketone
Terpinolene	0.04	Monoterpene
trans-Linalool oxide (fur.)	0.01	Monoterpenic alcohol
4-Nonanol	0.02	Aliphatic alcohol
Rosefuran	0.12	Monoterpenic ether
Perillene	0.10	Monoterpenic ether
Linalool	0.98	Monoterpenic alcohol
cis-Chrysanthemal?	0.03	Monoterpenic aldehyde
(*Z*)-6-Methyl-3,5-heptadien-2-one	0.03	Aliphatic ketone
trans-para-Mentha-2,8-dien-1-ol	0.03	Monoterpenic alcohol
Unknown	0.16	Unknown
exo-Isocitral	0.01	Monoterpenic aldehyde
trans-Chrysanthemal	0.37	Monoterpenic aldehyde
Citronellal	1.48	Monoterpenic aldehyde
Borneol	0.17	Monoterpenic alcohol
Isoneral	0.73	Monoterpenic aldehyde
α-Phellandren-8-ol	0.17	Monoterpenic alcohol
Rosefuran oxide	0.06	Monoterpenic ether

628 Boulevard du Saguenay, Saguenay (Qc) G7J 1H4 | www.phytochemia.com

Terpinen-4-ol	0.14	Monoterpenic alcohol
Unknown	0.09	Oxygenated monoterpene
Isogeranial	1.16	Monoterpenic aldehyde
Unknown	0.17	Unknown
α-Terpineol	0.20	Monoterpenic alcohol
Myrtenal	0.01	Monoterpenic aldehyde
trans-Isopiperitenol	0.02	Monoterpenic alcohol
Unknown	0.04	Oxygenated monoterpene
Decanal	0.19	Aliphatic aldehyde
cis-Isopiperitenol	0.02	Monoterpenic alcohol
2,3-Epoxyneral?	0.05	Monoterpenic aldehyde
Nerol	0.03	Monoterpenic alcohol
Citronellol	0.19	Monoterpenic alcohol
Neral	31.47	Monoterpenic aldehyde
Geraniol	5.91	Monoterpenic alcohol
Geranial	41.34	Monoterpenic aldehyde
Unknown	0.08	Oxygenated monoterpene
Geranyl formate	0.07	Monoterpenic ester
Unknown	0.03	Unknown
Neric acid	0.09	Monoterpenic acid
α-Cubebene	0.01	Sesquiterpene
Citronellyl acetate	0.06	Monoterpenic ester
Cyclosativene I	0.07	Sesquiterpene
Cyclosativene II	0.09	Sesquiterpene
Neryl acetate	0.09	Monoterpenic ester
Geranic acid	0.18	Aliphatic acid
α-Copaene	0.09	Sesquiterpene
β-Bourbonene	0.03	Sesquiterpene
Geranyl acetate	2.67	Monoterpenic ester
β-Cubebene	0.04	Sesquiterpene
β-Elemene	0.07	Sesquiterpene
Longifolene	0.03	Sesquiterpene
β-Caryophyllene	1.19	Sesquiterpene
β-Copaene	0.03	Sesquiterpene
trans-α-Bergamotene	0.01	Sesquiterpene
α-Humulene	0.14	Sesquiterpene
(*E*)-Isoeugenol	0.27	Phenylpropanoid
cis-Muurola-4(15),5-diene	0.04	Sesquiterpene
trans-Cadina-1(6),4-diene	0.05	Sesquiterpene
Germacrene D	0.15	Sesquiterpene
γ-Amorphene	0.02	Sesquiterpene
epi-Cubebol	0.10	Sesquiterpenic alcohol
α-Muurolene	0.04	Sesquiterpene
δ-Amorphene	0.02	Sesquiterpene
γ-Cadinene	1.06	Sesquiterpene
Cubebol	0.21	Sesquiterpenic alcohol
δ-Cadinene	0.24	Sesquiterpene
10-epi-Cubebol?	0.03	Sesquiterpenic alcohol
(*E*)-γ-Bisabolene	0.15	Sesquiterpene
Neryl butyrate	0.04	Monoterpenic ester
α-Elemol	0.04	Sesquiterpenic alcohol
Germacrene B	0.03	Sesquiterpene

Geranyl butyrate	0.11	Monoterpenic ester
Caryophyllene oxide	0.46	Sesquiterpenic ether
Humulene epoxide II	0.04	Sesquiterpenic ether
Selin-6-en-4α-ol isomer	0.02	Sesquiterpenic alcohol
1-epi-Cubenol	0.02	Sesquiterpenic alcohol
Cubenol	0.03	Sesquiterpenic alcohol
β-Eudesmol	0.01	Sesquiterpenic alcohol
α-Eudesmol	0.01	Sesquiterpenic alcohol
(2*Z*,6*Z*)-Farnesol	0.02	Sesquiterpenic alcohol
Farnesal isomer	0.03	Sesquiterpenic aldehyde
(2*E*,6*E*)-Farnesal	0.01	Sesquiterpenic aldehyde
Neophytadiene	0.01	Diterpene
meta-Camphorene	0.01	Diterpene
Unknown	0.04	Unknown
Dicitral	0.03	Diterpenic aldehyde
Phytol isomer	0.05	Diterpenic alcohol
Unknown	0.02	Unknown
Unknown	tr	Unknown
Unknown	0.01	Unknown
Unknown	0.01	Unknown
**Consolidated total**	**98.36%**	

tr: The compound has been detected below 0.005% of total signal.

Note: no correction factor was applied

About "consolidated" data: The table above presents the breakdown of the sample volatile constituents after applying an algorithm to collapse data acquired from the multi-columns system of PhytoChemia into a single set of consolidated contents. In case of discrepancies between columns, the algorithm is set to prioritize data from the most standard DB-5 column, and smallest values so as to avoid overestimating individual content. This process is semi-automatic. Advanced users are invited to consult the "Full analysis data" table after the chromatograms in this report to access the full untreated data and perform their own calculations if needed.

Unknowns: Unknown compounds' mass spectral data is presented in the "Full analysis data" table. The occurrence of unknown compounds is to be expected in many samples, and does not denote particular problems unless noted otherwise in the conclusion.

This page was intentionally left blank. The following pages present the complete data of the analysis.

628 Boulevard du Saguenay, Saguenay (Qc) G7J 1H4 | www.phytochemia.com

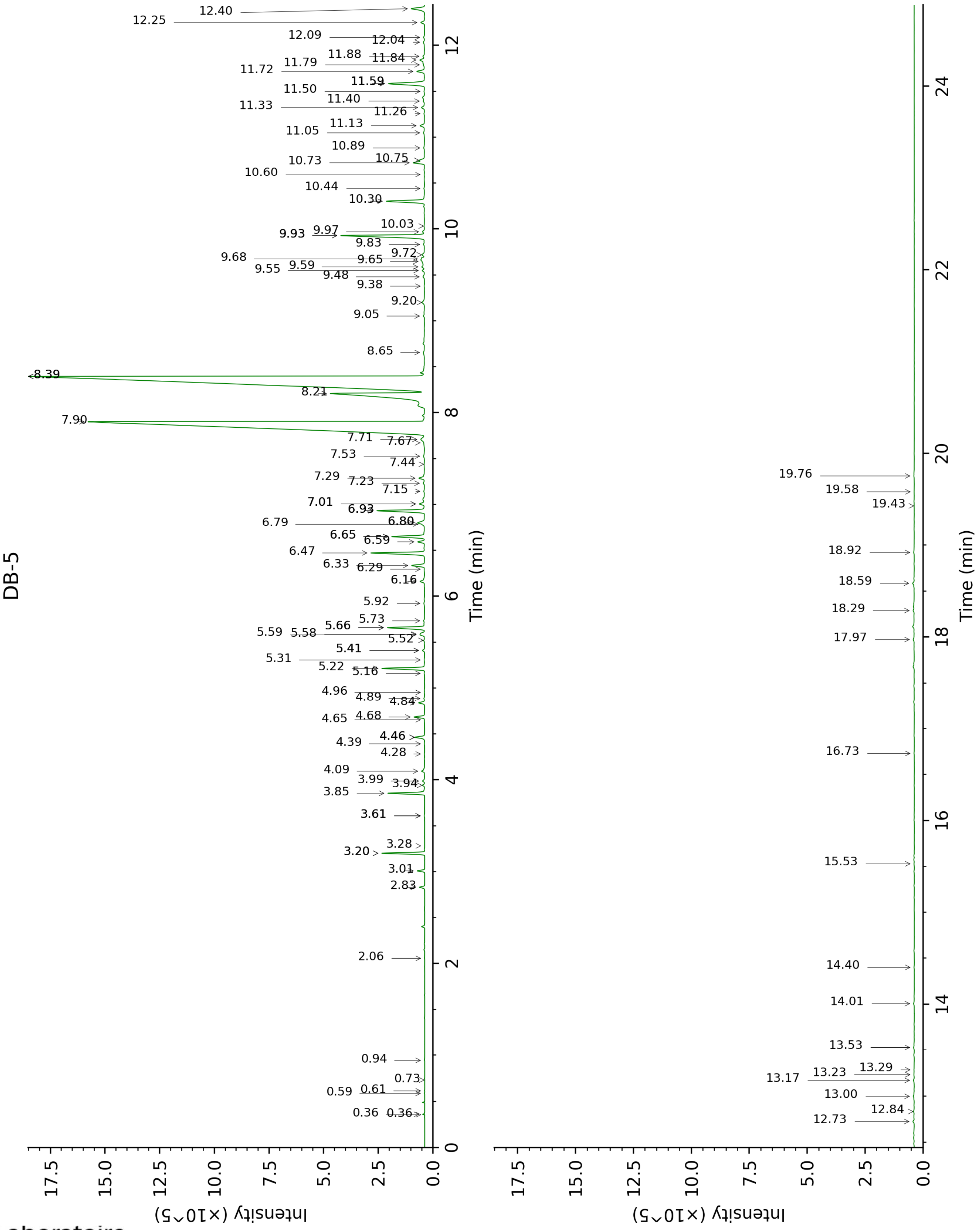
DB-5
Time (min)
Intensity (×10^5)
0.36 0.36 0.59 0.61 0.73 0.94 2.06 2.83 3.01 3.20 3.28 3.61 3.85 3.94 3.99 4.09 4.28 4.39 4.46 4.65 4.68 4.84 4.89 4.96 5.16 5.22 5.31 5.41 5.52 5.58 5.59 5.66 5.73 5.92 6.16 6.29 6.33 6.47 6.59 6.65 6.79 6.80 6.93 7.01 7.15 7.23 7.29 7.44 7.53 7.67 7.71 7.90 8.21 8.39 8.65 9.05 9.20 9.38 9.48 9.55 9.59 9.65 9.68 9.72 9.83 9.93 9.97 10.03 10.30 10.44 10.60 10.73 10.75 10.89 11.05 11.13 11.26 11.33 11.40 11.50 11.59 11.72 11.79 11.84 11.88 12.04 12.09 12.25 12.40
12.73 12.84 13.00 13.17 13.23 13.29 13.53 14.01 14.40 15.53 16.73 17.97 18.29 18.59 18.92 19.43 19.58 19.76

DB-WAX

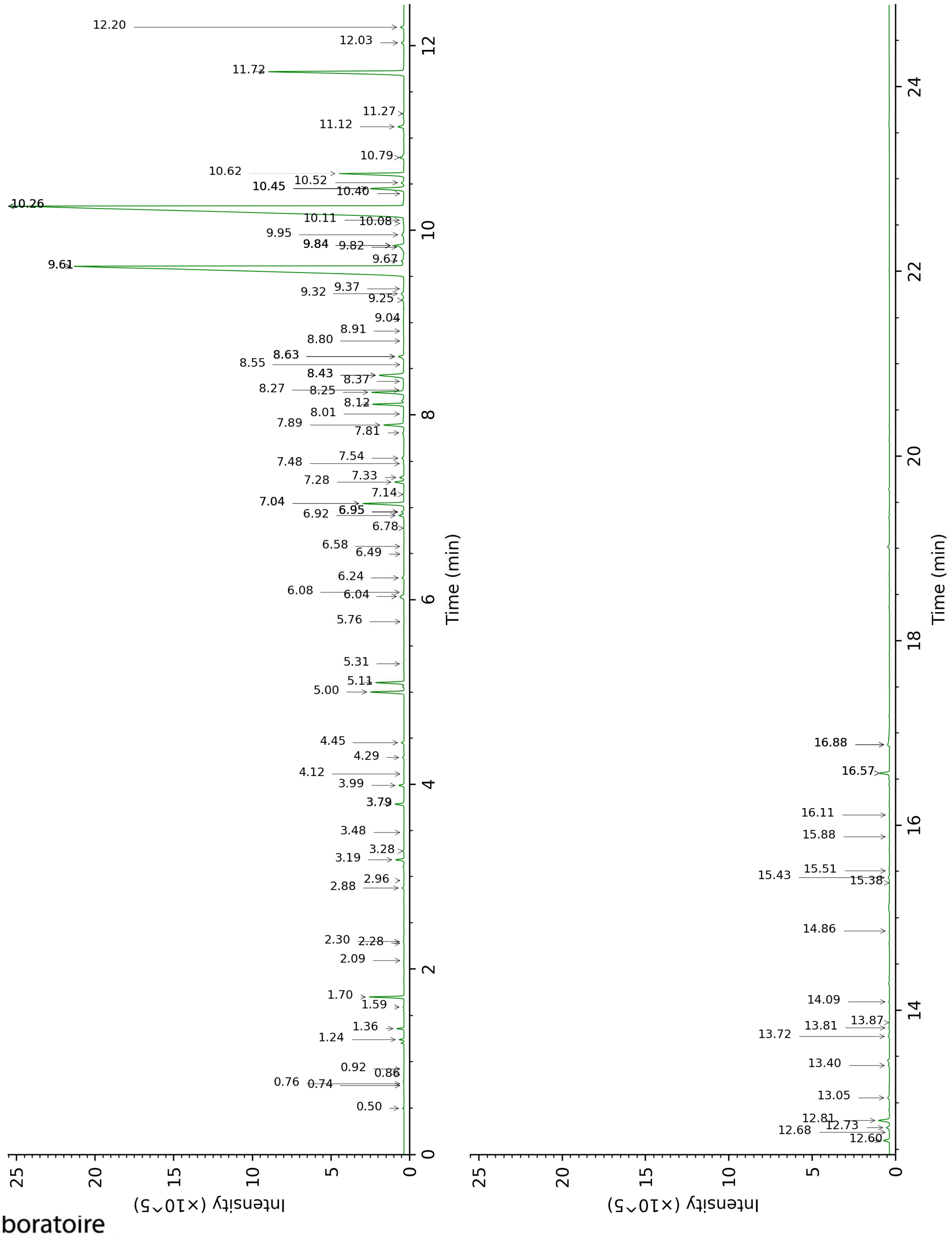
Time (min)
Intensity (×10^5)
0
5
10
15
20
25
0
2
4
6
8
10
12
12.20
12.03
11.72
11.27
11.12
10.79
10.62
10.52
10.45
10.40
10.26
10.11
10.08
9.95
9.84
9.82
9.67
9.61
9.37
9.32
9.25
9.04
8.91
8.80
8.63
8.55
8.43
8.37
8.27
8.25
8.12
8.01
7.89
7.81
7.54
7.48
7.33
7.28
7.14
7.04
6.95
6.92
6.78
6.58
6.49
6.24
6.08
6.04
5.76
5.31
5.11
5.00
4.45
4.29
4.12
3.99
3.79
3.48
3.28
3.19
2.96
2.88
2.30
2.28
2.09
1.70
1.59
1.36
1.24
0.92
0.86
0.76
0.74
0.50
Time (min)
Intensity (×10^5)
14
16
18
20
22
24
16.88
16.57
16.11
15.88
15.51
15.43
15.38
14.86
14.09
13.87
13.81
13.72
13.40
13.05
12.81
12.73
12.68
12.60

FULL ANALYSIS DATA

Identification	Column DB-5			Column DB-WAX		
	**R.T**	**R.I**	**%**	**R.T**	**R.I**	**%**
Ethanol	0.36	522	tr	0.86	909	tr
Acetone	0.36	522	0.02	0.50	786	0.02
Isovaleral	0.59	640	tr	0.76	890	0.01
2-Methylbutyral	0.62	652	tr	0.74	882	tr
2-Ethylfuran	0.73	700	tr	0.92	919	tr
Isoamyl alcohol	0.94	737	tr	3.48	1179	0.01
(3*Z*)-Hexenol	2.06	857	0.01	5.76	1343	0.01
Tricyclene	2.83	918	0.12	1.24	972	0.11
α-Pinene	3.01	930	0.16	1.36	991	0.16
Camphene	3.20*	942	0.94	1.70	1026	0.94
α-Fenchene	3.20*	942	[0.94]	1.59	1016	0.02
Thuja-2,4(10)-diene	3.28	948	0.01	2.28	1084	tr
β-Pinene	3.61*	969	0.02	2.09	1065	tr
Sabinene	3.61*	969	[0.02]	2.30	1086	0.02
6-Methyl-5-hepten-2-one	3.86	986	0.88	5.11	1296	0.87
Myrcene	3.94	991	0.06	2.88	1132	0.05
6-Methyl-5-hepten-2-ol	3.99	994	0.06	6.95*	1430	0.09
Octanal	4.10	1002	0.10	4.45	1249	0.08
α-Terpinene	4.28	1013	0.01	2.96	1138	0.01
para-Cymene	4.39	1020	0.01	4.12	1225	0.01
Limonene	4.46*	1025	0.27	3.19	1156	0.24
1,8-Cineole	4.46*	1025	[0.27]	3.28	1163	0.03
Benzeneacetaldehyde	4.65	1037	0.01	8.91	1578	0.01
(*Z*)-β-Ocimene	4.68	1039	0.27	3.79*	1202	0.27
(*E*)-β-Ocimene	4.84	1048	0.16	3.99	1216	0.15
2,6-Dimethyl-5-heptenal (melonal)	4.89	1052	0.02	5.31	1311	0.02
γ-Terpinene	4.96	1056	0.01	3.79*	1202	[0.27]
cis-Linalool oxide (fur.)	5.16	1069	0.01	6.58	1402	0.01
4-Nonanone	5.22	1072	1.10	5.00	1289	1.11
Camphenilone	5.31	1078	0.02	6.49	1396	0.01
Terpinolene	5.41*	1085	0.06	4.29	1238	0.04
trans-Linalool oxide (fur.)	5.41*	1085	[0.06]	6.95*	1430	[0.09]
4-Nonanol	5.52	1092	0.02			
Rosefuran	5.58	1096	0.12	6.04	1363	0.10
Perillene	5.59	1096	0.10	6.24	1377	0.06
Linalool	5.66*	1101	1.02	8.12	1516	0.98
cis-Chrysanthemal?	5.66*	1101	[1.02]	6.08	1366	0.03
(*Z*)-6-Methyl-3,5-heptadien-2-one	5.73	1106	0.03	8.27	1528	0.06
trans-para-Mentha-2,8-dien-1-ol	5.92	1118	0.03	9.04	1587	0.03
Unknown [m/z 81, 70 (98), 67 (63), 82 (53),	6.16	1133	0.16	6.92	1427	0.19

41 (46), 69 (46), 109 (43)...]						
exo-Isocitral	6.30	1142	0.01	7.54	1472	0.08
trans-Chrysanthemal	6.34	1145	0.37	7.28	1453	0.31
Citronellal	6.47	1154	1.48	7.04*	1436	1.47
Borneol	6.59	1162	0.17	9.84*	1652	0.39
Isoneral	6.65*	1165	0.90	7.89	1499	0.73
α-Phellandren-8-ol	6.65*	1165	[0.90]	10.26*	1686	41.30
Rosefuran oxide	6.79	1174	0.06	8.63*†	1556	0.24
Terpinen-4-ol	6.80*	1175	0.24	8.63*†	1556	[0.24]
Unknown [m/z 84, 83 (74), 137 (56), 41 (47), 93 (43), 108 (40)... 152 (2)]	6.80*	1175	[0.24]	9.67	1638	0.09
Isogeranial	6.94*	1184	1.33	8.25	1526	1.16
Unknown [m/z 69, 41 (65), 109 (36), 67 (16), 84 (11), 43 (10), 55 (9)...]	6.94*	1184	[1.33]			
α-Terpineol	7.01*†	1189	0.22	9.84*	1652	[0.39]
Myrtenal	7.01*†	1189	[0.22]	8.80	1569	0.01
trans-Isopiperitenol	7.15	1198	0.02	10.45*	1702	1.35
Unknown [m/z 84, 41 (83), 83 (79), 91 (76), 93 (67), 119 (64), 137 (63), 109 (54), 108 (54)... 152 (4)]	7.23	1204	0.04			
Decanal	7.29	1207	0.19	7.33	1457	0.14
cis-Isopiperitenol	7.44	1218	0.02	10.40	1697	0.01
2,3-Epoxyneral?	7.53	1224	0.05			
Nerol	7.67	1234	0.03	11.12	1758	0.23
Citronellol	7.71	1236	0.19	10.79	1730	0.16
Neral	7.90	1250	31.47	9.61*	1633	31.26
Geraniol	8.21	1271	5.91	11.72	1809	6.00
Geranial	8.40*	1284	41.42	10.26*	1686	[41.30]
Unknown [m/z 43, 69 (77), 41 (70), 109 (54)... 152 (6)]	8.40*	1284	[41.42]	13.05	1928	0.08
Geranyl formate	8.66	1302	0.07	9.95	1661	0.12
Unknown [m/z 82, 59 (44), 41 (43), 95 (31), 43 (29), 81 (24)...]	9.06	1332	0.03	12.74	1899	0.11
Neric acid	9.20	1336	0.09	16.57*	2272	0.36
α-Cubebene	9.38	1348	0.01	6.78	1416	0.01
Citronellyl acetate	9.48	1355	0.06	9.61*	1633	[31.26]
Cyclosativene I	9.55	1360	0.07	6.95*	1430	[0.09]
Cyclosativene II	9.59	1363	0.09	7.04*	1436	[1.47]
Neryl acetate	9.65	1367	0.09	10.26*	1686	[41.30]
Geranic acid	9.68	1369	0.18	16.88*	2305	0.23
α-Copaene	9.72	1372	0.09	7.14	1444	0.04
β-Bourbonene	9.83	1380	0.03	7.48	1468	0.03
Geranyl acetate	9.93*	1387	2.66	10.62	1715	2.67

β-Cubebene	9.93*	1387	[2.66]	7.81	1493	0.04
β-Elemene	9.97	1390	0.07	8.43*	1540	1.19
Longifolene	10.03	1394	0.03	8.01	1508	0.03
β-Caryophyllene	10.30	1414	1.19	8.43*	1540	[1.19]
β-Copaene	10.44	1424	0.03	8.37	1536	0.04
trans-α-Bergamotene	10.60	1435	0.01	8.55	1549	0.01
α-Humulene	10.73†	1445	0.47	9.32	1610	0.14
(*E*)-Isoeugenol	10.75†	1447	[0.47]	16.57*	2272	[0.36]
cis-Muurola-4(15),5-diene	10.89	1457	0.04	9.37	1614	0.02
trans-Cadina-1(6),4-diene	11.05	1469	0.05	9.25	1604	0.06
Germacrene D	11.13	1475	0.15	9.82	1650	0.10
γ-Amorphene	11.26	1485	0.02	9.84*	1652	[0.39]
epi-Cubebol	11.33	1490	0.10	12.03	1837	0.10
α-Muurolene	11.40	1495	0.04	10.11	1674	0.04
δ-Amorphene	11.50	1503	0.02	10.08	1671	0.02
γ-Cadinene	11.59*	1509	1.26	10.45*	1702	[1.35]
Cubebol	11.59*	1509	[1.26]	12.60	1886	0.21
δ-Cadinene	11.72	1520	0.24	10.45*	1702	[1.35]
10-epi-Cubebol?	11.79	1526	0.03	13.81	1998	0.04
(*E*)-γ-Bisabolene	11.84	1530	0.15	10.52	1707	0.10
Neryl butyrate	11.88	1533	0.04			
α-Elemol	12.04	1545	0.04	14.09	2025	0.03
Germacrene B	12.09	1549	0.03	11.27	1770	0.03
Geranyl butyrate	12.25	1562	0.11	12.20	1851	0.13
Caryophyllene oxide	12.40	1574	0.46	12.81	1906	0.41
Humulene epoxide II	12.73	1599	0.04	13.40	1960	0.04
Selin-6-en-4α-ol isomer	12.84	1608	0.02	14.86	2099	0.01
1-epi-Cubenol	13.00	1621	0.02	13.87	2004	0.02
Cubenol	13.17	1636	0.03	13.72	1989	0.04
β-Eudesmol	13.23	1641	0.01	15.51	2164	0.02
α-Eudesmol	13.29	1645	0.01	15.38	2151	0.01
(2*Z*,6*Z*)-Farnesol	13.53	1665	0.02	16.12	2226	0.01
Farnesal isomer	14.01	1705	0.03			
(2*E*,6*E*)-Farnesal	14.40	1739	0.01	15.88	2201	0.01
Neophytadiene	15.53	1839	0.01	12.68	1894	0.01
meta-Camphorene	16.73	1950	0.01	15.43	2156	0.06
Unknown [m/z 93, 69 (95), 135 (76), 107 (53), 41 (53), 109 (50)... 235 (10)...]	17.97	2072	0.04			
Dicitral	18.29	2104	0.03	16.88*	2305	[0.23]
Phytol isomer	18.59	2134	0.05			
Unknown [m/z 94, 43 (85), 93 (81), 69 (76), 137 (76), 95 (60), 134 (51)...]	18.92	2169	0.02			
Unknown [m/z 94, 43 (56), 123 (55), 69 (53), 95 (42), 79 (39)...]	19.43	2223	tr			

Unknown [m/z 93, 69 (79), 43 (70), 137 (53), 41 (41), 119 (37)... 289 (33)...]	19.58	2240	0.01	
Unknown [m/z 123, 94 (100), 43 (86), 69 (75), 95 (47), 41 (47), 93 (45)...]	19.76	2258	0.01	
**Total identified**	**98.06%**			**96.83%**
**Total reported**	**98.37%**			**97.30%**

*: Two or more compounds are coeluting on this column
[xx]: Duplicate percentage due to coelutions, not taken into account in the consolidated total
†: Peaks apexes were resolved, but peaks overlapped and were summed for analysis
tr: The compound has been detected below 0.005% of total signal.

Note: no correction factor was applied
R.T.: Retention time (minutes)
R.I.: Retention index

mountain rose herbs®
.com

An Herbs, Health, and Harmony Company.
Since 1987

CERTIFICATE OF ANALYSIS

Mailing: PO Box 50220 / Eugene, Oregon 97405
Phone: 800-879-3337 / Fax 510-217-4012
E-mail: qc@mountainroseherbs.com
www.mountainroseherbs.com

Product Name: Lime Peel Essential Oil
Botanical Name: *Citrus aurantifolia*
Origin: Mexico
Manufacture Date: March 2020
Part Used: Fruit Peel
Lot Number: EO2827
Extraction: Cold pressed
Grade: Certified Organic
Additives: None
Notes: None

Test	Results
Appearance	Greenish brown
Odor	Tart, sweet citrus
Density @20°C	0.858*
Refractive Index @20°C	39.9*
Optical Rotation @20°C	1.475

*By Vendor Report #200309

Rachelle Theobald (signature) 4/20/20

Christine Rice / Rachelle Theobald — Date
Quality Control Department

This information is presented in good faith and was compiled through testing methods in our laboratory, and with the assistance of our suppliers, harvesters, and processors information. We make no warranty, either expressed or implied in the complete accuracy of the information listed herein. The data in this analysis is offered solely for your verification and consideration. It is the responsibility of the buyer to provide themselves with up to date analyses for any botanicals purchased through Mountain Rose Herbs.

RK

mountain rose herbs .com

CERTIFICATE OF ANALYSIS

Mailing: PO Box 50220 / Eugene, Oregon 97405
Phone: 800-879-3337 / Fax 510-217-4012
E-mail: qc@mountainroseherbs.com
www.mountainroseherbs.com

Product Name: Myrrh Essential Oil
Botanical Name: *Commiphora myrrha*
Origin: France
Production Date: May 2020
Part Used: Gum
Lot Number: EO2921
Extraction: Distillation
Grade: Certified Organic
Additives: None

Test	Specifications	Results	Method
Identity	Passed	Passed	Organoleptic

Rachelle Theobald — 12/2/20

Steven Yeager / Rachelle Theobald / Geri Green **Date**
Quality Control Department

This information is presented in good faith and was compiled through testing methods in our laboratory, contracted laboratories, and with the assistance of our suppliers, harvesters, and processors information. We make no warranty, either expressed or implied in the complete accuracy of the information listed herein. The data in this analysis is offered solely for your verification and consideration. It is the responsibility of the buyer to provide themselves with up to date analyses for any botanicals purchased through Mountain Rose Herbs.

smt

mountain rose herbs®
.com

CERTIFICATE OF ANALYSIS

Mailing: PO Box 50220 / Eugene, Oregon 97405
Phone: 800-879-3337 / Fax 510-217-4012
E-mail: qc@mountainroseherbs.com
www.mountainroseherbs.com

Product Name: Peppermint Essential Oil
Botanical Name: *Mentha piperita*
Origin: Hungary
Production Date: November 2020
Part Used: Aerial Portion
Lot Number: EO2970
Extraction: Distillation
Grade: Certified Organic
Additives: None

Test	Specifications	Results	Method
Identity	Passed	Passed	Organoleptic

Rachelle Theobald　　　　4/2/21

Steven Yeager / Rachelle Theobald / Geri Green　　　**Date**
Quality Control Department

This information is presented in good faith and was compiled through testing methods in our laboratory, contracted laboratories, and with the assistance of our suppliers, harvesters, and processors information. We make no warranty, either expressed or implied in the complete accuracy of the information listed herein. The data in this analysis is offered solely for your verification and consideration. It is the responsibility of the buyer to provide themselves with up to date analyses for any botanicals purchased through Mountain Rose Herbs.

smt

mountain rose herbs.com

CERTIFICATE OF ANALYSIS

Mailing: PO Box 50220 / Eugene, Oregon 97405
Phone: 800-879-3337 / Fax 510-217-4012
E-mail: qc@mountainroseherbs.com
www.mountainroseherbs.com

Product Name: Spearmint Essential Oil
Botanical Name: *Mentha spicata*
Origin: India
Production Date: July 2020
Part Used: Flowering Plant
Lot Number: EO3000
Extraction: Distillation
Grade: Certified Organic
Additives: None

Test	Specifications	Results	Method
Identity	Passed	Passed	Organoleptic

Rachelle Theobald   4/28/21

Steven Yeager / Rachelle Theobald / Geri Green   **Date**
Quality Control Department

This information is presented in good faith and was compiled through testing methods in our laboratory, contracted laboratories, and with the assistance of our suppliers, harvesters, and processors information. We make no warranty, either expressed or implied in the complete accuracy of the information listed herein. The data in this analysis is offered solely for your verification and consideration. It is the responsibility of the buyer to provide themselves with up to date analyses for any botanicals purchased through Mountain Rose Herbs.

α-Pinene	0.83
β-Pinene	1.04
Sabinene	0.39
1-p-Menthene	0.12
β-Myrcene	1.40
α-Terpinene	0.16
Limonene	19.33
1,8 Cineole	1.70
1,3,8-p-Menthatriene	0.03
Cis-Ocimene	0.05
γ-Terpinene	0.24
3-Octanone	0.10
para-Cymene	0.40
trans-Sabinene Hydrate	0.14
Menthone	0.41
Isomenthone	0.44
alpha-Copaene	0.12
β-Bourbonene	0.98
Neoisomenthol	0.76
Terpinene–4-Ol	0.69
β-Caryophyllene	1.69
Cis-Dihydrocarvone	1.56
Menthol	0.53
trans-β-Farnesene	0.17
α-Terpineol	0.41
Germacrene D	0.34
Piperitone	0.75
Carvone	58.15
delta-Cadinene	0.23
cis-Carvyl acetate	0.11
trans-Carveol	0.29
cis-Carveol	0.13
trans-Jasmone	0.09

Spearmint Essential Oil- EO3000

mountain rose herbs ®
.com

An Herbs, Health, and Harmony Company.
Since 1987

CERTIFICATE OF ANALYSIS

Mailing: PO Box 50220 / Eugene, Oregon 97405
Phone: 800-879-3337 / Fax 510-217-4012
E-mail: qc@mountainroseherbs.com
www.mountainroseherbs.com

Product Name: Tea Tree Essential Oil
Botanical Name: *Melaleuca alternifolia*
Origin: China
Manufacture Date: April 2020
Part Used: Leaf and Twig
Lot Number: EO2836
Extraction: Distillation
Grade: Certified Organic
Additives: None
Notes: None

Test	Results
Appearance	Translucent, almost colorless
Odor	Warm, fresh, spicy-camphoraceous
Specific Gravity @25°C	0.8954*
Refractive Index @20°C	1.478*

*By Vendor Report #2004000584

Rachelle Theobald — 6/5/20

Christine Rice / Rachelle Theobald — **Date**
Quality Control Department

This information is presented in good faith and was compiled through testing methods in our laboratory, and with the assistance of our suppliers, harvesters, and processors information. We make no warranty, either expressed or implied in the complete accuracy of the information listed herein. The data in this analysis is offered solely for your verification and consideration. It is the responsibility of the buyer to provide themselves with up to date analyses for any botanicals purchased through Mountain Rose Herbs.

rk

Date : May 29, 2020

Certificate of Analysis – GC Profiling

Sample identification

Internal code : 20E20-MRH01
Customer identification : Tea Tree - South Africa - EO2836
Type : Essential oil
Source : *Melaleuca alternifolia* ct. Terpinen-4-ol
Customer : Mountain Rose Herbs

Analysis

Method: PC-MAT-007 - Analysis of the composition of an essential oil or other volatile liquide by FAST GC-FID (in French); identifications validated by GC-MS.
Analyst : Fanny Charlier, B. Sc.
Analysis date : May 21, 2020

Checked and approved by :

Alexis St-Gelais, M. Sc., chimiste 2013-174

Notes: This report may not be published, including online, without the written consent from Laboratoire PhytoChemia. This report is digitally signed, it is only considered valid if the digital signature is intact. The results only describe the samples that were submitted to the assays.

PHYSICOCHEMICAL DATA

Physical aspect: Faintly yellow liquid
Refractive index: 1.4789 ± 0.0003 (20 °C; method PC-MAT-016)

CONCLUSION

No adulterant, contaminant or diluent has been detected using this method.

628 Boulevard du Saguenay, Saguenay (Qc) G7J 1H4 | www.phytochemia.com

ANALYSIS SUMMARY – CONSOLIDATED CONTENTS

New readers of similar reports are encouraged to read table footnotes at least once.

Identification	%	Class
Isobutyral	0.01	Aliphatic aldehyde
2-Methylbutyral	0.01	Aliphatic aldehyde
(3*Z*)-Hexenol	0.02	Aliphatic alcohol
α-Thujene	0.88	Monoterpene
α-Pinene	2.30	Monoterpene
Camphene	0.02	Monoterpene
α-Fenchene	0.01	Monoterpene
β-Pinene	0.66	Monoterpene
Sabinene	0.54	Monoterpene
3-Methyl-3-cyclohexenone	0.02	Aliphatic ketone
Myrcene	0.83	Monoterpene
Pseudolimonene	0.02	Monoterpene
α-Phellandrene	0.44	Monoterpene
(3*Z*)-Hexenyl acetate	0.02	Aliphatic ester
α-Terpinene	9.08	Monoterpene
Carvomenthene	0.03	Aliphatic alcohol
para-Cymene	1.87	Monoterpene
β-Phellandrene	0.02	Monoterpene
Limonene	0.65	Monoterpene
1,8-Cineole	2.52	Monoterpenic ether
(*Z*)-β-Ocimene	0.02	Monoterpene
(*E*)-β-Ocimene	0.02	Monoterpene
γ-Terpinene	18.40	Monoterpene
cis-Sabinene hydrate	0.08	Monoterpenic alcohol
Terpinolene	3.17	Monoterpene
para-Cymenene	0.05	Monoterpene
trans-Sabinene hydrate	0.19	Monoterpenic alcohol
Linalool	0.08	Monoterpenic alcohol
endo-Fenchol	0.01	Monoterpenic alcohol
cis-para-Menth-2-en-1-ol	0.33	Monoterpenic alcohol
4-Hydroxy-4-methylcyclohex-2-enone	0.02	Aliphatic alcohol
trans-Pinocarveol	0.04	Monoterpenic alcohol
trans-para-Menth-2-en-1-ol	0.24	Monoterpenic alcohol
Unknown	0.03	Unknown
Borneol	0.02	Monoterpenic alcohol
δ-Terpineol	0.02	Monoterpenic alcohol
Terpinen-4-ol	40.82	Monoterpenic alcohol
Dill ether	0.02	Monoterpenic ether
para-Cymen-8-ol	0.05	Monoterpenic alcohol
α-Terpineol	2.68	Monoterpenic alcohol
cis-Piperitol	0.09	Monoterpenic alcohol
Unknown	0.01	Oxygenated monoterpene
trans-Piperitol	0.13	Monoterpenic alcohol
exo-2-Hydroxycineole	0.02	Monoterpenic alcohol
Nerol	0.03	Monoterpenic alcohol

628 Boulevard du Saguenay, Saguenay (Qc) G7J 1H4 | www.phytochemia.com

Unknown	0.02	Oxygenated monoterpene
Piperitone	0.03	Monoterpenic ketone
cis-Carvenone oxide?	0.01	Monoterpenic ketone
trans-Ascaridole glycol	0.04	Monoterpenic alcohol
cis-Ascaridole glycol	0.02	Monoterpenic alcohol
Carvacrol	0.01	Monoterpenic alcohol
Unknown	0.02	Monoterpenic alcohol
Bicycloelemene	0.02	Sesquiterpene
α-Cubebene	0.05	Sesquiterpene
Unknown	0.02	Unknown
Isoledene	0.08	Sesquiterpene
α-Copaene	0.11	Sesquiterpene
7-Cubebene	0.07	Sesquiterpene
7-Cubebene epimer?	0.03	Aliphatic alcohol
β-Elemene	0.05	Sesquiterpene
α-Gurjunene	0.40	Sesquiterpene
Methyleugenol	0.08	Phenylpropanoid
β-Maaliene	0.03	Sesquiterpene
β-Caryophyllene	0.35	Sesquiterpene
γ-Maaliene	0.08	Sesquiterpene
β-Gurjunene	0.02	Sesquiterpene
α-Maaliene	0.08	Sesquiterpene
Aromadendrene	1.28	Sesquiterpene
Selina-5,11-diene	0.19	Sesquiterpene
Cadina-3,5-diene isomer I?	0.19	Sesquiterpene
trans-Muurola-3,5-diene	0.11	Sesquiterpene
α-Humulene	0.12	Sesquiterpene
allo-Aromadendrene	0.59	Sesquiterpene
Valerena-4,7(11)-diene	0.04	Sesquiterpene
γ-Gurjunene	0.06	Sesquiterpene
trans-Cadina-1(6),4-diene	0.34	Sesquiterpene
γ-Muurolene	0.03	Sesquiterpene
β-Selinene	0.11	Sesquiterpene
allo-Aromadendr-9-ene	0.19	Sesquiterpene
δ-Selinene	0.15	Sesquiterpene
Bicyclogermacrene	0.82	Sesquiterpene
α-Selinene	0.12	Sesquiterpene
Viridiflorene	1.07	Sesquiterpene
α-Muurolene	0.18	Sesquiterpene
γ-Cadinene	0.05	Sesquiterpene
trans-Calamenene	0.10	Sesquiterpene
δ-Cadinene	1.18	Sesquiterpene
Zonarene	0.23	Sesquiterpene
trans-Cadina-1,4-diene	0.21	Sesquiterpene
α-Calacorene	0.03	Sesquiterpene
Epiglobulol	0.13	Sesquiterpenic alcohol
Maaliol	0.06	Sesquiterpenic alcohol
Unknown	0.05	Oxygenated sesquiterpene
Spathulenol	0.12	Sesquiterpenic alcohol
Globulol	0.64	Sesquiterpenic alcohol
Gleenol	0.03	Sesquiterpenic alcohol
Viridiflorol	0.27	Sesquiterpenic alcohol

Cubeban-11-ol	0.23	Sesquiterpenic alcohol
Eudesm-5-en-11-ol analog	0.23	Sesquiterpenic alcohol
Rosifoliol	0.24	Sesquiterpenic alcohol
1-epi-Cubenol	0.27	Sesquiterpenic alcohol
Cubenol	0.15	Sesquiterpenic alcohol
α-Muurolol	0.03	Sesquiterpenic alcohol
**Consolidated total**	**97.95%**	

Note: no correction factor was applied

About "consolidated" data: The table above presents the breakdown of the sample volatile constituents after applying an algorithm to collapse data acquired from the multi-columns system of PhytoChemia into a single set of consolidated contents. In case of discrepancies between columns, the algorithm is set to prioritize data from the most standard DB-5 column, and smallest values so as to avoid overestimating individual content. This process is semi-automatic. Advanced users are invited to consult the “Full analysis data” table after the chromatograms in this report to access the full untreated data and perform their own calculations if needed.

Unknowns: Unknown compounds’ mass spectral data is presented in the “Full analysis data” table. The occurrence of unknown compounds is to be expected in many samples, and does not denote particular problems unless noted otherwise in the conclusion.

628 Boulevard du Saguenay, Saguenay (Qc) G7J 1H4 | www.phytochemia.com

This page was intentionally left blank. The following pages present the complete data of the analysis.

628 Boulevard du Saguenay, Saguenay (Qc) G7J 1H4 | www.phytochemia.com

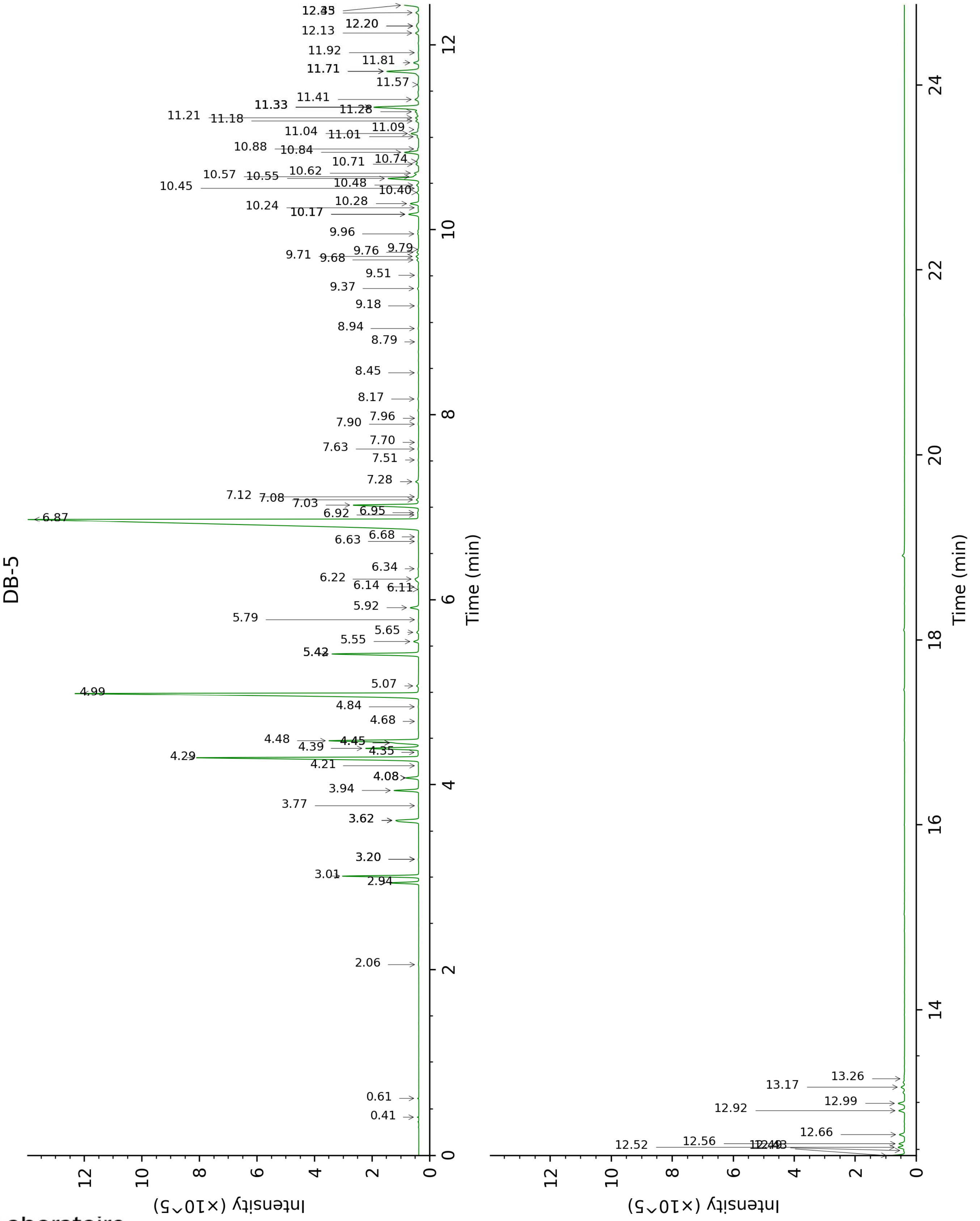
DB-5
Time (min)
Intensity (×10^5)
12.20
12.13
11.92
11.81
11.71
11.57
11.41
11.33
11.28
11.21
11.18
11.09
11.04
11.01
10.88
10.84
10.74
10.71
10.62
10.57
10.55
10.48
10.45
10.40
10.28
10.24
10.17
9.96
9.79
9.76
9.71
9.68
9.51
9.37
9.18
8.94
8.79
8.45
8.17
7.96
7.90
7.70
7.63
7.51
7.28
7.12
7.08
7.03
6.95
6.92
6.87
6.68
6.63
6.34
6.22
6.14
6.11
5.92
5.79
5.65
5.55
5.42
5.07
4.99
4.84
4.68
4.48
4.45
4.39
4.35
4.29
4.21
4.08
3.94
3.77
3.62
3.20
3.01
2.94
2.06
0.61
0.41
Time (min)
Intensity (×10^5)
13.26
13.17
12.99
12.92
12.66
12.56
12.52

DB-WAX

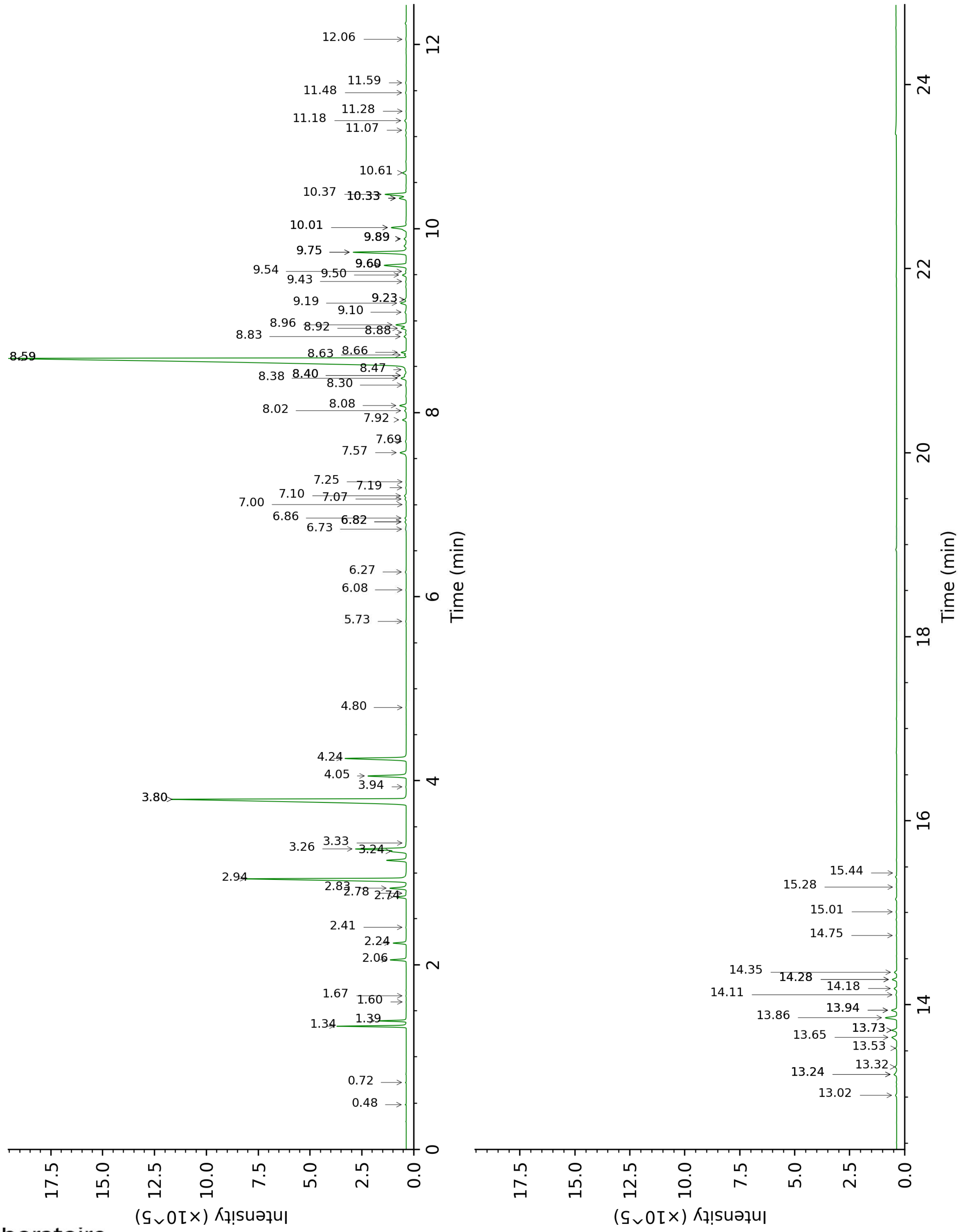
12.06
11.59
11.48
11.28
11.18
11.07
10.61
10.37
10.33
10.01
9.89
9.75
9.60
9.54
9.50
9.43
9.23
9.19
9.10
8.96
8.92
8.88
8.83
8.66
8.63
8.59
8.47
8.40
8.38
8.30
8.08
8.02
7.92
7.69
7.57
7.25
7.19
7.10
7.07
7.00
6.86
6.82
6.73
6.27
6.08
5.73
4.80
4.24
4.05
3.94
3.80
3.33
3.26
3.24
2.94
2.83
2.78
2.74
2.41
2.24
2.06
1.67
1.60
1.39
1.34
0.72
0.48
Time (min)
Intensity (×10^5)
15.44
15.28
15.01
14.75
14.35
14.28
14.18
14.11
13.94
13.86
13.73
13.65
13.53
13.32
13.24
13.02
Time (min)
Intensity (×10^5)

FULL ANALYSIS DATA

Identification	Column DB-5			Column DB-WAX		
	R.T	R.I	%	R.T	R.I	%
Isobutyral	0.41	530	0.01	0.48	779	0.02
2-Methylbutyral	0.61	652	0.01	0.72	878	0.01
(3*Z*)-Hexenol	2.06	858	0.02	5.73	1344	0.03
α-Thujene	2.94	925	0.88	1.39	998	0.88
α-Pinene	3.01	930	2.30	1.34	989	2.27
Camphene	3.20*	942	0.02	1.67	1024	0.02
α-Fenchene	3.20*	942	[0.02]	1.60	1018	0.01
β-Pinene	3.62*	970	1.20	2.06	1063	0.66
Sabinene	3.62*	970	[1.20]	2.24	1081	0.54
3-Methyl-3-cyclohexenone	3.78	980	0.02	6.08	1368	0.02
Myrcene	3.94	991	0.83	2.83	1131	0.83
Pseudolimonene	4.08*	1000	0.46	2.78	1126	0.02
α-Phellandrene	4.08*	1000	[0.46]	2.74	1123	0.44
(3*Z*)-Hexenyl acetate	4.21	1009	0.02	4.80	1279	0.01
α-Terpinene	4.29	1014	9.08	2.94	1139	9.05
Carvomenthene	4.35	1018	0.03	2.41	1098	0.02
para-Cymene	4.39	1020	1.87	4.05	1225	1.89
β-Phellandrene	4.45*†	1024	4.11	3.33	1170	0.02
Limonene	4.45*†	1024	[4.11]	3.24	1162	0.65
1,8-Cineole	4.48†	1026	[4.11]	3.26	1164	2.52
(*Z*)-β-Ocimene	4.68	1039	0.02	3.80*	1206	18.36
(*E*)-β-Ocimene	4.84	1049	0.02	3.94	1216	0.03
γ-Terpinene	4.99	1058	18.40	3.80*	1206	[18.36]
cis-Sabinene hydrate	5.07	1063	0.08	6.86	1426	0.07
Terpinolene	5.42*	1085	3.20	4.24	1239	3.17
para-Cymenene	5.42*	1085	[3.20]	6.27	1382	0.05
trans-Sabinene hydrate	5.55	1094	0.19	7.92	1505	0.19
Linalool	5.65	1100	0.08	8.02	1513	0.09
endo-Fenchol	5.79	1108	0.01	8.40*	1542	0.12
cis-para-Menth-2-en-1-ol	5.92	1117	0.33	8.08	1517	0.36
4-Hydroxy-4-methylcyclohex-2-enone	6.11	1130	0.02	14.11	2032	0.05
trans-Pinocarveol	6.14	1132	0.04	9.23*†	1607	[0.39]
trans-para-Menth-2-en-1-ol	6.22	1137	0.24	8.92	1582	0.25
Unknown [m/z 109, 124 (45), 119 (41), 43 (35), 91 (28), 95 (25)...]	6.34	1144	0.03	6.82*	1422	0.08
Borneol	6.63	1163	0.02	9.75*	1648	2.81
δ-Terpineol	6.68	1167	0.02	9.43	1623	0.04
Terpinen-4-ol	6.87	1179	40.82	8.59*	1557	42.17

Dill ether	6.92	1182	0.02	7.25	1455	0.02
para-Cymen-8-ol	6.94	1184	0.05	11.48	1793	0.05
α-Terpineol	7.03	1189	2.68	9.75*	1648	[2.81]
cis-Piperitol	7.08	1193	0.09	9.60*	1637	1.30
Unknown [m/z 121, 43 (99), 91 (85), 77 (73), 93 (41), 136 (33)... 166 (3)]	7.12	1195	0.01			
trans-Piperitol	7.28	1206	0.13	10.33*	1696	0.41
exo-2-Hydroxycineole	7.51	1222	0.02	11.59	1802	0.05
Nerol	7.63	1230	0.03	11.07	1758	0.04
Unknown [m/z 137, 152 (28), 43 (25), 91 (24), 109 (23), 119 (19)]	7.70	1235	0.02	11.28	1776	0.02
Piperitone	7.90	1249	0.03	9.89*†	1660	0.26
cis-Carvenone oxide?	7.96	1253	0.01			
trans-Ascaridole glycol	8.17	1267	0.04	14.18	2039	0.14
cis-Ascaridole glycol	8.45	1287	0.02	14.75	2094	0.02
Carvacrol	8.79	1305	0.01	15.44	2162	0.01
Unknown [m/z 97, 112 (92), 83 (62), 43 (44), 41 (25)... 170? (4)]	8.94	1316	0.02	15.01	2120	0.04
Bicycloelemene	9.18	1333	0.02	7.00	1436	0.02
α-Cubebene	9.37	1346	0.05	6.73	1416	0.07
Unknown [m/z 43, 95 (62), 107 (45), 110 (41), 55 (28), 67 (25)...]	9.51	1356	0.02	13.94*	2016	0.29
Isoledene	9.68	1368	0.08	6.82*	1422	[0.08]
α-Copaene	9.71	1370	0.11	7.10	1444	0.11
7-Cubebene	9.76	1374	0.07	7.06	1441	0.07
7-Cubebene epimer?	9.79	1376	0.03	7.19	1450	0.03
β-Elemene	9.96	1388	0.05	8.40*	1542	[0.12]
α-Gurjunene	10.17*	1403	0.42	7.57	1478	0.40
Methyleugenol	10.17*	1403	[0.42]	13.32	1958	0.08
β-Maaliene	10.24	1408	0.03	7.69	1487	0.05
β-Caryophyllene	10.28	1411	0.35	8.38	1540	0.33
γ-Maaliene	10.40	1420	0.08	8.47	1547	0.07
β-Gurjunene	10.45	1423	0.02	8.30	1534	0.04
α-Maaliene	10.48	1426	0.08	8.63	1560	0.08
Aromadendrene	10.56	1431	1.28	8.59*	1557	[42.17]
Selina-5,11-diene	10.57	1433	0.19	8.66	1562	0.22
Cadina-3,5-diene isomer I?	10.62	1436	0.19			

trans-Muurola-3,5-diene	10.71	1443	0.11	8.83	1575	0.11
α-Humulene	10.74	1445	0.12	9.23*†	1607	[0.39]
allo-Aromadendrene	10.84	1453	0.59	8.96	1585	0.58
Valerena-4,7(11)-diene	10.88	1455	0.04	8.88	1579	0.04
γ-Gurjunene	11.01	1465	0.06	9.10	1596	0.10
trans-Cadina-1(6),4-diene	11.04	1468	0.34	9.19†	1604	0.39
γ-Muurolene	11.09	1471	0.03	9.54	1632	0.04
β-Selinene	11.18	1478	0.11	9.89*†	1660	[0.26]
allo-Aromadendr-9-ene	11.21	1480	0.19	9.50	1628	0.21
δ-Selinene	11.28	1485	0.15	9.60*	1637	[1.30]
Bicyclogermacrene	11.33*	1489	2.11	10.01*	1670	1.00
α-Selinene	11.33*	1489	[2.11]	9.89*†	1660	[0.26]
Viridiflorene	11.33*	1489	[2.11]	9.60*	1637	[1.30]
α-Muurolene	11.41	1495	0.18	10.01*	1670	[1.00]
γ-Cadinene	11.57	1507	0.05	10.33*	1696	[0.41]
trans-Calamenene	11.72*	1519	1.53	11.18	1767	0.10
δ-Cadinene	11.72*	1519	[1.53]	10.37	1700	1.18
Zonarene	11.72*	1519	[1.53]	10.33*	1696	[0.41]
trans-Cadina-1,4-diene	11.81	1526	0.21	10.61	1720	0.21
α-Calacorene	11.92	1534	0.03	12.06	1844	0.03
Epiglobulol	12.13	1551	0.13	13.24*	1951	0.19
Maaliol	12.20*	1557	0.19	13.02	1930	0.06
Unknown [m/z 161, 109 (98), 82 (93), 43 (72), 105 (68), 93 (59), 69 (56), 119 (55)... 222 (7)]	12.20*	1557	[0.19]	13.24*	1951	[0.19]
Spathulenol	12.35	1568	0.12	14.35	2056	0.10
Globulol	12.43	1575	0.64	13.86	2008	0.62
Gleenol	12.49	1579	0.03	13.53	1977	0.04
Viridiflorol	12.52	1582	0.27	13.94*	2016	[0.29]
Cubeban-11-ol	12.56	1585	0.23	13.73*†	1996	[0.60]
Eudesm-5-en-11-ol analog	12.66	1593	0.23	14.28*	2048	0.23
Rosifoliol	12.92	1614	0.24	14.28*	2048	[0.23]
1-epi-Cubenol	12.99	1620	0.27	13.73*†	1996	[0.60]
Cubenol	13.17	1634	0.15	13.65†	1988	0.60
α-Muurolol	13.26	1642	0.03	15.28	2146	0.01
**Total identified**		**98.89%**			**97.71%**	
**Total reported**		**98.98%**			**97.76%**	

*: Two or more compounds are coeluting on this column
[xx]: Duplicate percentage due to coelutions, not taken into account in the consolidated total
†: Peaks apexes were resolved, but peaks overlapped and were summed for analysis

Note: no correction factor was applied
R.T.: Retention time (minutes)

R.I.: Retention index

628 Boulevard du Saguenay, Saguenay (Qc) G7J 1H4 | www.phytochemia.com
