# Supplementary material for: Antifungal Activity of Select Essential Oils against Candida auris and Their Interactions with Antifungal Drugs
Source: Pathogens. 2022 Jul 22;11(8):821. doi: 10.3390/pathogens11080821 (PMC9331469; doi:10.3390/pathogens11080821)
Supplement: Supplementary file 1 [file pathogens-11-00821-s001.zip › S4/Spearmint EO GCMS- EO3000.pdf]

α-Pinene	0.83
β-Pinene	1.04
Sabinene	0.39
1-p-Menthene	0.12
β-Myrcene	1.40
α-Terpinene	0.16
Limonene	19.33
1,8 Cineole	1.70
1,3,8-p-Menthatriene	0.03
Cis-Ocimene	0.05
γ-Terpinene	0.24
3-Octanone	0.10
para-Cymene	0.40
trans-Sabinene Hydrate	0.14
Menthone	0.41
Isomenthone	0.44
alpha-Copaene	0.12
β-Bourbonene	0.98
Neoisomenthol	0.76
Terpinene-4-Ol	0.69
β-Caryophyllene	1.69
Cis-Dihydrocarvone	1.56
Menthol	0.53
trans-β-Farnesene	0.17
α-Terpineol	0.41
Germacrene D	0.34
Piperitone	0.75
Carvone	58.15
delta-Cadinene	0.23
cis-Carvyl acetate	0.11
trans-Carveol	0.29
cis-Carveol	0.13
trans-Jasmone	0.09

Spearmint Essential Oil- EO3000
