# Supplementary material for: Antifungal Activity of Select Essential Oils against Candida auris and Their Interactions with Antifungal Drugs
Source: Pathogens. 2022 Jul 22;11(8):821. doi: 10.3390/pathogens11080821 (PMC9331469; doi:10.3390/pathogens11080821)
Supplement: Supplementary file 1 [file pathogens-11-00821-s001.zip › S4/Tea Tree GCMS- EO2836.pdf]

Date : May 29, 2020

Certificate of Analysis – GC Profiling

Sample identification

Internal code : 20E20-MRH01
Customer identification : Tea Tree - South Africa - EO2836
Type : Essential oil
Source : *Melaleuca alternifolia* ct. Terpinen-4-ol
Customer : Mountain Rose Herbs

Analysis

Method: PC-MAT-007 - Analysis of the composition of an essential oil or other volatile liquide by FAST GC-FID (in French); identifications validated by GC-MS.
Analyst : Fanny Charlier, B. Sc.
Analysis date : May 21, 2020

Checked and approved by :

Alexis St-Gelais, M. Sc., chimiste 2013-174

Notes: This report may not be published, including online, without the written consent from Laboratoire PhytoChemia. This report is digitally signed, it is only considered valid if the digital signature is intact. The results only describe the samples that were submitted to the assays.

Laboratoire PhytoChemia
628 Boulevard du Saguenay Ouest, Saguenay (Qc) G7J 1H4 | www.phytochemia.com

PHYSICOCHEMICAL DATA

Physical aspect: Faintly yellow liquid
Refractive index: 1.4789 ± 0.0003 (20 °C; method PC-MAT-016)

CONCLUSION

No adulterant, contaminant or diluent has been detected using this method.

628 Boulevard du Saguenay, Saguenay (Qc) G7J 1H4 | www.phytochemia.com

Analysis summary – Consolidated contents

New readers of similar reports are encouraged to read table footnotes at least once.

Identification	%	Class
Isobutyral	0.01	Aliphatic aldehyde
2-Methylbutyral	0.01	Aliphatic aldehyde
(3*Z*)-Hexenol	0.02	Aliphatic alcohol
α-Thujene	0.88	Monoterpene
α-Pinene	2.30	Monoterpene
Camphene	0.02	Monoterpene
α-Fenchene	0.01	Monoterpene
β-Pinene	0.66	Monoterpene
Sabinene	0.54	Monoterpene
3-Methyl-3-cyclohexenone	0.02	Aliphatic ketone
Myrcene	0.83	Monoterpene
Pseudolimonene	0.02	Monoterpene
α-Phellandrene	0.44	Monoterpene
(3*Z*)-Hexenyl acetate	0.02	Aliphatic ester
α-Terpinene	9.08	Monoterpene
Carvomenthene	0.03	Aliphatic alcohol
para-Cymene	1.87	Monoterpene
β-Phellandrene	0.02	Monoterpene
Limonene	0.65	Monoterpene
1,8-Cineole	2.52	Monoterpenic ether
(*Z*)-β-Ocimene	0.02	Monoterpene
(*E*)-β-Ocimene	0.02	Monoterpene
γ-Terpinene	18.40	Monoterpene
cis-Sabinene hydrate	0.08	Monoterpenic alcohol
Terpinolene	3.17	Monoterpene
para-Cymenene	0.05	Monoterpene
trans-Sabinene hydrate	0.19	Monoterpenic alcohol
Linalool	0.08	Monoterpenic alcohol
endo-Fenchol	0.01	Monoterpenic alcohol
cis-para-Menth-2-en-1-ol	0.33	Monoterpenic alcohol
4-Hydroxy-4-methylcyclohex-2-enone	0.02	Aliphatic alcohol
trans-Pinocarveol	0.04	Monoterpenic alcohol
trans-para-Menth-2-en-1-ol	0.24	Monoterpenic alcohol
Unknown	0.03	Unknown
Borneol	0.02	Monoterpenic alcohol
δ-Terpineol	0.02	Monoterpenic alcohol
Terpinen-4-ol	40.82	Monoterpenic alcohol
Dill ether	0.02	Monoterpenic ether
para-Cymen-8-ol	0.05	Monoterpenic alcohol
α-Terpineol	2.68	Monoterpenic alcohol
cis-Piperitol	0.09	Monoterpenic alcohol
Unknown	0.01	Oxygenated monoterpene
trans-Piperitol	0.13	Monoterpenic alcohol
exo-2-Hydroxycineole	0.02	Monoterpenic alcohol
Nerol	0.03	Monoterpenic alcohol

Unknown	0.02	Oxygenated monoterpene
Piperitone	0.03	Monoterpenic ketone
cis-Carvenone oxide?	0.01	Monoterpenic ketone
trans-Ascaridole glycol	0.04	Monoterpenic alcohol
cis-Ascaridole glycol	0.02	Monoterpenic alcohol
Carvacrol	0.01	Monoterpenic alcohol
Unknown	0.02	Monoterpenic alcohol
Bicycloelemene	0.02	Sesquiterpene
α-Cubebene	0.05	Sesquiterpene
Unknown	0.02	Unknown
Isoledene	0.08	Sesquiterpene
α-Copaene	0.11	Sesquiterpene
7-Cubebene	0.07	Sesquiterpene
7-Cubebene epimer?	0.03	Aliphatic alcohol
β-Elemene	0.05	Sesquiterpene
α-Gurjunene	0.40	Sesquiterpene
Methyleugenol	0.08	Phenylpropanoid
β-Maaliene	0.03	Sesquiterpene
β-Caryophyllene	0.35	Sesquiterpene
γ-Maaliene	0.08	Sesquiterpene
β-Gurjunene	0.02	Sesquiterpene
α-Maaliene	0.08	Sesquiterpene
Aromadendrene	1.28	Sesquiterpene
Selina-5,11-diene	0.19	Sesquiterpene
Cadina-3,5-diene isomer I?	0.19	Sesquiterpene
trans-Muurola-3,5-diene	0.11	Sesquiterpene
α-Humulene	0.12	Sesquiterpene
allo-Aromadendrene	0.59	Sesquiterpene
Valerena-4,7(11)-diene	0.04	Sesquiterpene
γ-Gurjunene	0.06	Sesquiterpene
trans-Cadina-1(6),4-diene	0.34	Sesquiterpene
γ-Muurolene	0.03	Sesquiterpene
β-Selinene	0.11	Sesquiterpene
allo-Aromadendr-9-ene	0.19	Sesquiterpene
δ-Selinene	0.15	Sesquiterpene
Bicyclogermacrene	0.82	Sesquiterpene
α-Selinene	0.12	Sesquiterpene
Viridiflorene	1.07	Sesquiterpene
α-Muurolene	0.18	Sesquiterpene
γ-Cadinene	0.05	Sesquiterpene
trans-Calamenene	0.10	Sesquiterpene
δ-Cadinene	1.18	Sesquiterpene
Zonarene	0.23	Sesquiterpene
trans-Cadina-1,4-diene	0.21	Sesquiterpene
α-Calacorene	0.03	Sesquiterpene
Epiglobulol	0.13	Sesquiterpenic alcohol
Maaliol	0.06	Sesquiterpenic alcohol
Unknown	0.05	Oxygenated sesquiterpene
Spathulenol	0.12	Sesquiterpenic alcohol
Globulol	0.64	Sesquiterpenic alcohol
Gleenol	0.03	Sesquiterpenic alcohol
Viridiflorol	0.27	Sesquiterpenic alcohol

Cubeban-11-ol	0.23	Sesquiterpenic alcohol
Eudesm-5-en-11-ol analog	0.23	Sesquiterpenic alcohol
Rosifoliol	0.24	Sesquiterpenic alcohol
1-epi-Cubenol	0.27	Sesquiterpenic alcohol
Cubenol	0.15	Sesquiterpenic alcohol
α-Muurolol	0.03	Sesquiterpenic alcohol
**Consolidated total**	**97.95%**	

Note: no correction factor was applied

About "consolidated" data: The table above presents the breakdown of the sample volatile constituents after applying an algorithm to collapse data acquired from the multi-columns system of PhytoChemia into a single set of consolidated contents. In case of discrepancies between columns, the algorithm is set to prioritize data from the most standard DB-5 column, and smallest values so as to avoid overestimating individual content. This process is semi-automatic. Advanced users are invited to consult the “Full analysis data” table after the chromatograms in this report to access the full untreated data and perform their own calculations if needed.

Unknowns: Unknown compounds’ mass spectral data is presented in the “Full analysis data” table. The occurrence of unknown compounds is to be expected in many samples, and does not denote particular problems unless noted otherwise in the conclusion.

628 Boulevard du Saguenay, Saguenay (Qc) G7J 1H4 | www.phytochemia.com

This page was intentionally left blank. The following pages present the complete data of the analysis.

628 Boulevard du Saguenay, Saguenay (Qc) G7J 1H4 | www.phytochemia.com

DB-5

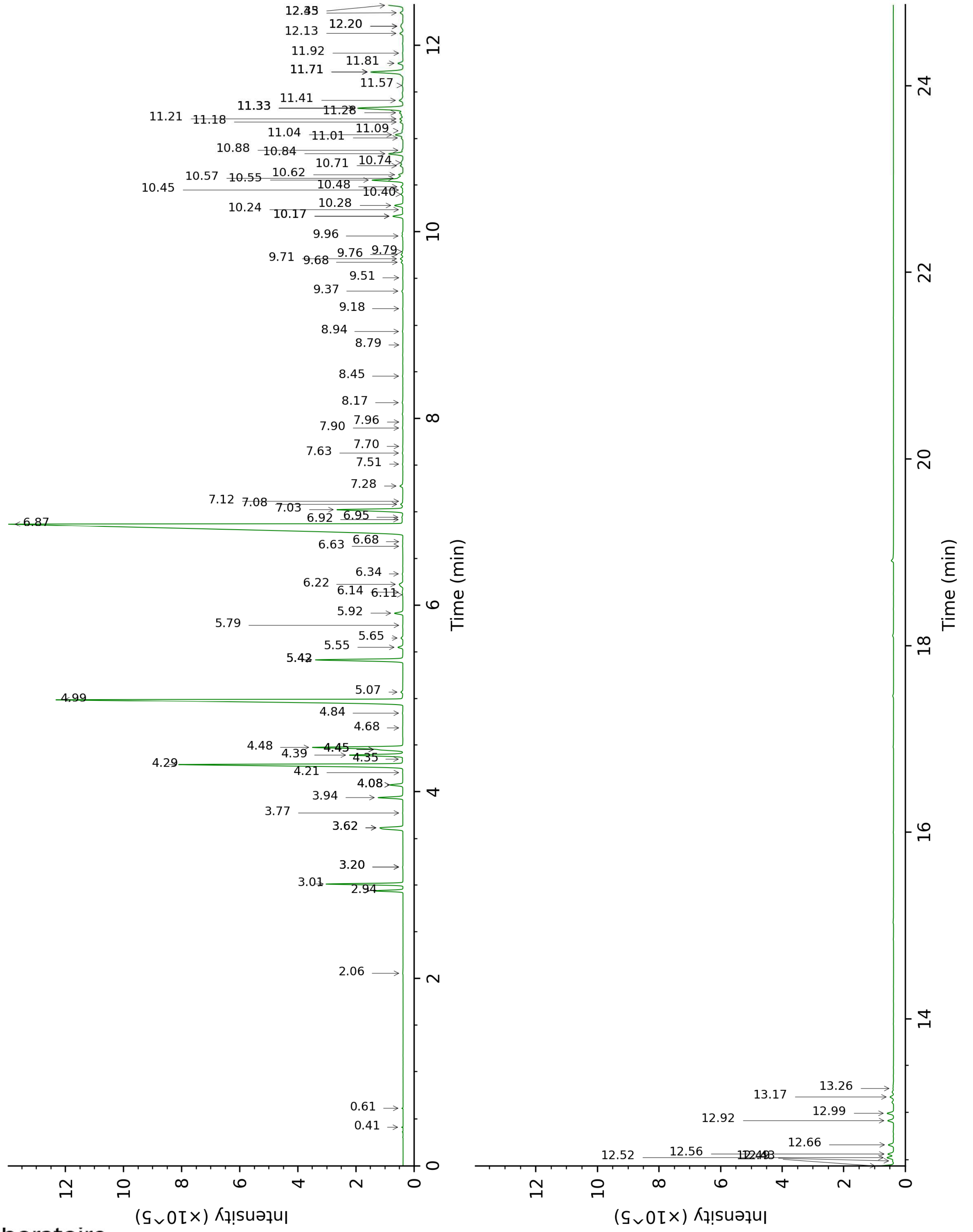

DB-WAX

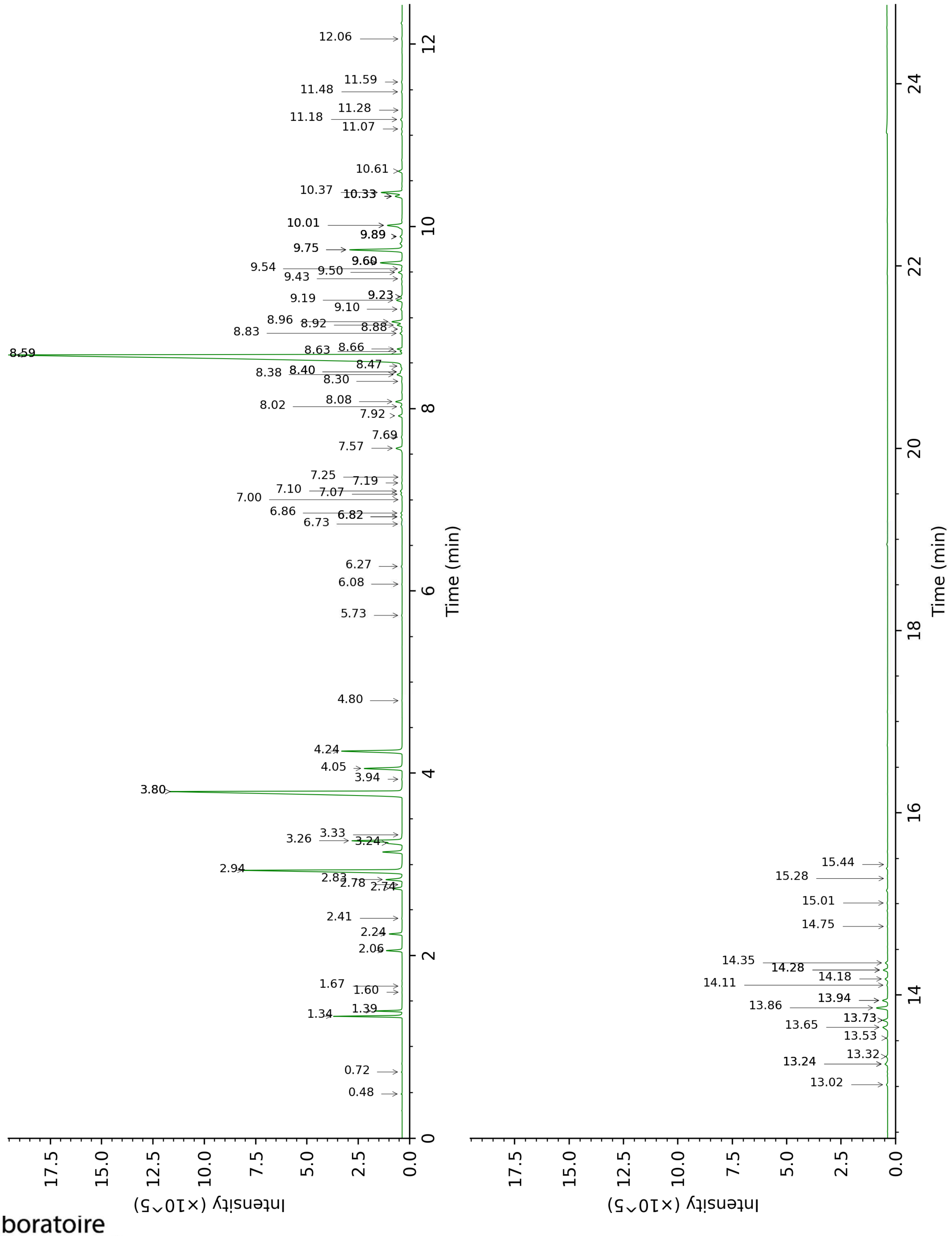

628 Boulevard du Saguenay, Saguenay (Qc) G7J 1H4 | www.phytochemia.com

FULL ANALYSIS DATA

Identification	Column DB-5			Column DB-WAX		
	R.T	R.I	%	R.T	R.I	%
Isobutyral	0.41	530	0.01	0.48	779	0.02
2-Methylbutyral	0.61	652	0.01	0.72	878	0.01
(3*Z*)-Hexenol	2.06	858	0.02	5.73	1344	0.03
α-Thujene	2.94	925	0.88	1.39	998	0.88
α-Pinene	3.01	930	2.30	1.34	989	2.27
Camphene	3.20*	942	0.02	1.67	1024	0.02
α-Fenchene	3.20*	942	[0.02]	1.60	1018	0.01
β-Pinene	3.62*	970	1.20	2.06	1063	0.66
Sabinene	3.62*	970	[1.20]	2.24	1081	0.54
3-Methyl-3-cyclohexenone	3.78	980	0.02	6.08	1368	0.02
Myrcene	3.94	991	0.83	2.83	1131	0.83
Pseudolimonene	4.08*	1000	0.46	2.78	1126	0.02
α-Phellandrene	4.08*	1000	[0.46]	2.74	1123	0.44
(3*Z*)-Hexenyl acetate	4.21	1009	0.02	4.80	1279	0.01
α-Terpinene	4.29	1014	9.08	2.94	1139	9.05
Carvomenthene	4.35	1018	0.03	2.41	1098	0.02
para-Cymene	4.39	1020	1.87	4.05	1225	1.89
β-Phellandrene	4.45*†	1024	4.11	3.33	1170	0.02
Limonene	4.45*†	1024	[4.11]	3.24	1162	0.65
1,8-Cineole	4.48†	1026	[4.11]	3.26	1164	2.52
(*Z*)-β-Ocimene	4.68	1039	0.02	3.80*	1206	18.36
(*E*)-β-Ocimene	4.84	1049	0.02	3.94	1216	0.03
γ-Terpinene	4.99	1058	18.40	3.80*	1206	[18.36]
cis-Sabinene hydrate	5.07	1063	0.08	6.86	1426	0.07
Terpinolene	5.42*	1085	3.20	4.24	1239	3.17
para-Cymenene	5.42*	1085	[3.20]	6.27	1382	0.05
trans-Sabinene hydrate	5.55	1094	0.19	7.92	1505	0.19
Linalool	5.65	1100	0.08	8.02	1513	0.09
endo-Fenchol	5.79	1108	0.01	8.40*	1542	0.12
cis-para-Menth-2-en-1-ol	5.92	1117	0.33	8.08	1517	0.36
4-Hydroxy-4-methylcyclohex-2-enone	6.11	1130	0.02	14.11	2032	0.05
trans-Pinocarveol	6.14	1132	0.04	9.23*†	1607	[0.39]
trans-para-Menth-2-en-1-ol	6.22	1137	0.24	8.92	1582	0.25
Unknown [m/z 109, 124 (45), 119 (41), 43 (35), 91 (28), 95 (25)...]	6.34	1144	0.03	6.82*	1422	0.08
Borneol	6.63	1163	0.02	9.75*	1648	2.81
δ-Terpineol	6.68	1167	0.02	9.43	1623	0.04
Terpinen-4-ol	6.87	1179	40.82	8.59*	1557	42.17

Dill ether	6.92	1182	0.02	7.25	1455	0.02
para-Cymen-8-ol	6.94	1184	0.05	11.48	1793	0.05
α-Terpineol	7.03	1189	2.68	9.75*	1648	[2.81]
cis-Piperitol	7.08	1193	0.09	9.60*	1637	1.30
Unknown [m/z 121, 43 (99), 91 (85), 77 (73), 93 (41), 136 (33)... 166 (3)]	7.12	1195	0.01			
trans-Piperitol	7.28	1206	0.13	10.33*	1696	0.41
exo-2-Hydroxycineole	7.51	1222	0.02	11.59	1802	0.05
Nerol	7.63	1230	0.03	11.07	1758	0.04
Unknown [m/z 137, 152 (28), 43 (25), 91 (24), 109 (23), 119 (19)]	7.70	1235	0.02	11.28	1776	0.02
Piperitone	7.90	1249	0.03	9.89*†	1660	0.26
cis-Carvenone oxide?	7.96	1253	0.01			
trans-Ascaridole glycol	8.17	1267	0.04	14.18	2039	0.14
cis-Ascaridole glycol	8.45	1287	0.02	14.75	2094	0.02
Carvacrol	8.79	1305	0.01	15.44	2162	0.01
Unknown [m/z 97, 112 (92), 83 (62), 43 (44), 41 (25)... 170? (4)]	8.94	1316	0.02	15.01	2120	0.04
Bicycloelemene	9.18	1333	0.02	7.00	1436	0.02
α-Cubebene	9.37	1346	0.05	6.73	1416	0.07
Unknown [m/z 43, 95 (62), 107 (45), 110 (41), 55 (28), 67 (25)...]	9.51	1356	0.02	13.94*	2016	0.29
Isoledene	9.68	1368	0.08	6.82*	1422	[0.08]
α-Copaene	9.71	1370	0.11	7.10	1444	0.11
7-Cubebene	9.76	1374	0.07	7.06	1441	0.07
7-Cubebene epimer?	9.79	1376	0.03	7.19	1450	0.03
β-Elemene	9.96	1388	0.05	8.40*	1542	[0.12]
α-Gurjunene	10.17*	1403	0.42	7.57	1478	0.40
Methyleugenol	10.17*	1403	[0.42]	13.32	1958	0.08
β-Maaliene	10.24	1408	0.03	7.69	1487	0.05
β-Caryophyllene	10.28	1411	0.35	8.38	1540	0.33
γ-Maaliene	10.40	1420	0.08	8.47	1547	0.07
β-Gurjunene	10.45	1423	0.02	8.30	1534	0.04
α-Maaliene	10.48	1426	0.08	8.63	1560	0.08
Aromadendrene	10.56	1431	1.28	8.59*	1557	[42.17]
Selina-5,11-diene	10.57	1433	0.19	8.66	1562	0.22
Cadina-3,5-diene isomer I?	10.62	1436	0.19			

trans-Muurola-3,5-diene	10.71	1443	0.11	8.83	1575	0.11
α-Humulene	10.74	1445	0.12	9.23*†	1607	[0.39]
allo-Aromadendrene	10.84	1453	0.59	8.96	1585	0.58
Valerena-4,7(11)-diene	10.88	1455	0.04	8.88	1579	0.04
γ-Gurjunene	11.01	1465	0.06	9.10	1596	0.10
trans-Cadina-1(6),4-diene	11.04	1468	0.34	9.19†	1604	0.39
γ-Muurolene	11.09	1471	0.03	9.54	1632	0.04
β-Selinene	11.18	1478	0.11	9.89*†	1660	[0.26]
allo-Aromadendr-9-ene	11.21	1480	0.19	9.50	1628	0.21
δ-Selinene	11.28	1485	0.15	9.60*	1637	[1.30]
Bicyclogermacrene	11.33*	1489	2.11	10.01*	1670	1.00
α-Selinene	11.33*	1489	[2.11]	9.89*†	1660	[0.26]
Viridiflorene	11.33*	1489	[2.11]	9.60*	1637	[1.30]
α-Muurolene	11.41	1495	0.18	10.01*	1670	[1.00]
γ-Cadinene	11.57	1507	0.05	10.33*	1696	[0.41]
trans-Calamenene	11.72*	1519	1.53	11.18	1767	0.10
δ-Cadinene	11.72*	1519	[1.53]	10.37	1700	1.18
Zonarene	11.72*	1519	[1.53]	10.33*	1696	[0.41]
trans-Cadina-1,4-diene	11.81	1526	0.21	10.61	1720	0.21
α-Calacorene	11.92	1534	0.03	12.06	1844	0.03
Epiglobulol	12.13	1551	0.13	13.24*	1951	0.19
Maaliol	12.20*	1557	0.19	13.02	1930	0.06
Unknown [m/z 161, 109 (98), 82 (93), 43 (72), 105 (68), 93 (59), 69 (56), 119 (55)... 222 (7)]	12.20*	1557	[0.19]	13.24*	1951	[0.19]
Spathulenol	12.35	1568	0.12	14.35	2056	0.10
Globulol	12.43	1575	0.64	13.86	2008	0.62
Gleenol	12.49	1579	0.03	13.53	1977	0.04
Viridiflorol	12.52	1582	0.27	13.94*	2016	[0.29]
Cubeban-11-ol	12.56	1585	0.23	13.73*†	1996	[0.60]
Eudesm-5-en-11-ol analog	12.66	1593	0.23	14.28*	2048	0.23
Rosifoliol	12.92	1614	0.24	14.28*	2048	[0.23]
1-epi-Cubenol	12.99	1620	0.27	13.73*†	1996	[0.60]
Cubenol	13.17	1634	0.15	13.65†	1988	0.60
α-Muurolol	13.26	1642	0.03	15.28	2146	0.01
**Total identified**		**98.89%**			**97.71%**	
**Total reported**		**98.98%**			**97.76%**	

*: Two or more compounds are coeluting on this column
[xx]: Duplicate percentage due to coelutions, not taken into account in the consolidated total
†: Peaks apexes were resolved, but peaks overlapped and were summed for analysis

Note: no correction factor was applied
R.T.: Retention time (minutes)

R.I.: Retention index

628 Boulevard du Saguenay, Saguenay (Qc) G7J 1H4 | www.phytochemia.com
